# Novel Nanomolar Allosteric Modulators of AMPA Receptor of Bis(pyrimidine) Series: Synthesis, Biotesting and SAR Analysis

**DOI:** 10.3390/molecules27238252

**Published:** 2022-11-26

**Authors:** Kseniya N. Sedenkova, Denis V. Zverev, Anna A. Nazarova, Mstislav I. Lavrov, Eugene V. Radchenko, Yuri K. Grishin, Alexey V. Gabrel’yan, Vladimir L. Zamoyski, Vladimir V. Grigoriev, Elena B. Averina, Vladimir A. Palyulin

**Affiliations:** 1 Department of Chemistry, Lomonosov Moscow State University, Leninskie Gory 1-3, 119991 Moscow, Russia; 2 Institute of Physiologically Active Compounds at Federal Research Center of Problems of Chemical Physics and Medicinal Chemistry, Russian Academy of Sciences, Severny proezd 1, Chernogolovka, 142432 Moscow, Russia

**Keywords:** pyrimidines, S_N_Ar reactions, bis(pyrimidine), bivalent ligand, ionotropic glutamate receptors, AMPA receptor, allosteric modulators, PAM

## Abstract

Positive allosteric modulators (PAMs) of AMPA receptors represent attractive candidates for the development of drugs for the treatment of cognitive and neurodegenerative disorders. Dimeric molecules have been reported to have an especially potent modulating effect, due to the U-shaped form of the AMPA receptor’s allosteric binding site. In the present work, novel bis(pyrimidines) were studied as AMPA receptor modulators. A convenient and flexible preparative approach to bis(pyrimidines) containing a hydroquinone linker was elaborated, and a series of derivatives with varied substituents was obtained. The compounds were examined in the patch clamp experiments for their influence on the kainate-induced currents, and 10 of them were found to have potentiating properties. The best potency was found for 2-methyl-4-(4-((2-methyl-5,6,7,8-tetrahydroquinazolin-4-yl)oxy)phenoxy)-6,7,8,9-tetrahydro-5*H*-cyclohepta[*d*]pyrimidine, which potentiated the kainate-induced currents by up to 77% in all tested concentrations (10^−12^–10^−6^ M). The results were rationalized via the modeling of modulator complexes with the dimeric ligand binding domain of the GluA2 AMPA receptor, using molecular docking and molecular dynamics simulation. The prediction of ADMET, physicochemical, and PAINS properties of the studied bis(pyrimidines) confirmed that PAMs of this type may act as the potential lead compounds for the development of neuroprotective drugs.

## 1. Introduction

The glutamatergic system is the main excitatory mediator system in the mammalian brain. It plays an important role in the functioning of the central nervous system (CNS) and the pathogenesis of many neurological and neurodegenerative diseases. The diversity of its functions is supported by a wide range of receptors belonging to two families: ionotropic glutamate receptors (iGluRs), which represent ligand-gated ion channels, and metabotropic glutamate receptors (mGluRs), which are G protein-coupled receptors [1,2,3,4]. One of the subtypes of iGluRs are AMPA receptors (AMPARs), selectively activated by α-amino-3-hydroxy-5-methyl-4-isoxazolepropionic acid (AMPA), which are present in the CNS in the largest amount, and are characterized by the fastest signal transmission. Among the ligands acting on AMPA receptors and causing a therapeutic effect, positive allosteric modulators (PAMs) are especially interesting [5,6]. They represent a synthetic class of small-molecule drugs that penetrate the blood–brain barrier and enhance fast excitatory synaptic responses mediated by glutamate receptors [7,8]. Their binding site has been identified and shown to be positioned appropriately to slow the deactivation and desensitization of the receptors [9,10,11]. Allosteric modulators can stimulate respiration in rodents after an opioid overdose [12]. Furthermore, these ligands can enhance the expression of synaptic long-term potentiation (LTP) [6,13], the neuronal expression of brain-derived neurotrophic factor (BDNF) [14,15,16], and also exhibit antidepressant effects [17,18,19]. Finally, it is important to note that positive allosteric modulators can improve the processes of learning and memory formation while having a neuroprotective effect [20,21,22], which makes them attractive candidates for the development of drugs for the treatment of cognitive and neurodegenerative disorders. On the other hand, negative allosteric modulators (NAMs) of the AMPA receptors possess anticonvulsant activity and can be used as antiepileptic drugs [23,24,25,26].

The PAM binding site of the AMPA receptors is known to have a U-shaped form that enables effective binding of dimeric molecules and makes such structures attractive for the search of positive allosteric modulators [22,27,28,29,30,31]. Earlier, we elaborated the PAM pharmacophore and QSAR models [32,33,34], as well as molecular modeling approaches, for the search of potential PAM chemotypes, and developed a number of novel series of dimeric and large monomeric AMPA receptor PAMs based on different scaffolds, which demonstrated potency in the nanomolar or picomolar concentration ranges in patch clamp experiments [28,29,30,31,35,36,37,38].

**Figure 1:**
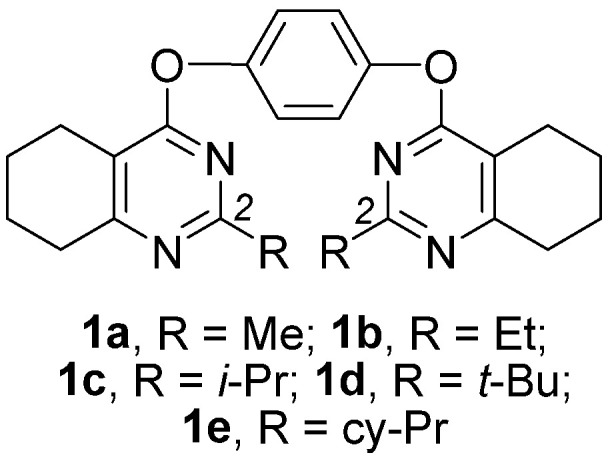
Allosteric modulators of AMPA receptor of bis(pyrimidine) series.

In particular, we found a novel type of bivalent allosteric modulators of AMPA receptor **1a**–**e**, containing tetrahydroquinazoline moieties (Figure 1) [30]. It was shown that the modulating properties of compounds **1a**–**e** drastically depended on the substituents in position *2* of tetrahydroquinazoline: while compounds **1a**,**b**,**d** demonstrated activity as positive modulators, compounds **1c**,**e** were found to act as negative modulators of AMPAR. 

In this connection, in the course of the present work we aimed to obtain a broader series of bis(pyrimidines) with varying substituents, and to investigate the relation between bis(pyrimidine) structure and modulating activity. 

## 2. Results and Discussion

### 2.1. Chemistry

In the preceding work [30], the preparative approaches to compounds **1a**–**e** based on S_N_Ar reactions involving hydroquinone as the nucleophile were described. Nevertheless, the target compounds were obtained in low-to-moderate preparative yields, and the procedures of their purification were rather complicated. Moreover, only symmetric bis(pyrimidines) could be obtained via the described method.

**Scheme 1:**
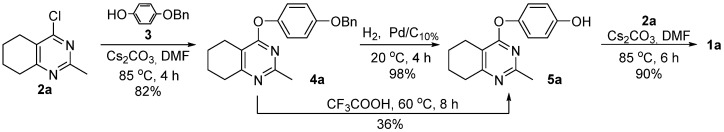
Synthesis of bis(pyrimidine) **1a**.

That is why an alternative synthetic approach to bis(pyrimidines) was elaborated, using the example of compound **1a**. The proposed scheme of synthesis of compound **1a** included (1) nucleophilic substitution of chlorine in 4-chloropyrimidine **2a** upon the treatment with monobenzylated hydroquinone **3**; (2) removal of the Bn protecting group; and (3) S_N_Ar reaction between the obtained compound **5a** and one more equivalent of 4-chloropyrimidine **2a** (Scheme 1).

Monobenzylated hydroquinone **3** in the presence of Cs_2_CO_3_ [39] was used as the nucleophile in the first step of the proposed synthetic scheme (Scheme 1). After the optimization of the reagent ratio (see Supplementary Materials), the reaction between heterocycle **2a** and monobenzylated hydroquinone **3** afforded product **4a**, bearing a Bn-protected hydroxyl group, in high yield.

Two conventional methods of removal of the protective group were probed: reflux in trifluoroacetic acid (TFA) [40] and hydrogenolysis [41] (Scheme 1). The treatment of compound **4a** with TFA did not lead to complete conversion of the starting compound—the conversion did not exceed 80% when the reaction was carried out for 8–18 h. Moreover, a number of unidentified products were present in the reaction mixture. As a result, phenol **5a** was isolated via column chromatography (SiO_2_, CHCl_3_-MeOH 40:1) in low yield (36%). On the other hand, hydrogenolysis of compound **4a** in the presence of Pd/C_10%_ proceeded smoothly in high yield, and no additional purification of product **5a** was required (Scheme 1).

The obtained heterocycle **5a**, bearing a free hydroxyl group, was successfully involved in the S_N_Ar reaction with 4-chloropyrimidine **2a** in the presence of Cs_2_CO_3_, to afford target bis(pyrimidine) **1a** in high isolated yield (Scheme 1). The optimization of the reagent ratio was also carried out (see Supplementary Materials).

**Scheme 2:**
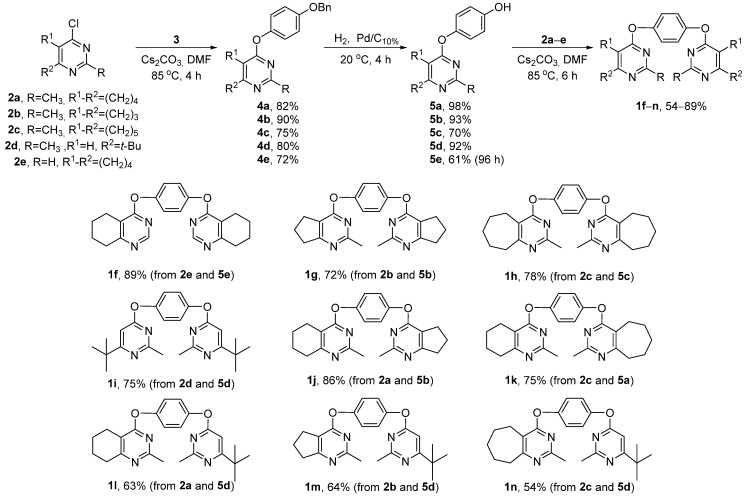
Synthesis of bis(pyrimidines) **1f**–**n**.

The elaborated preparative approach was applied to obtain a series of novel bis(pyrimidines) **1f**–**n** of symmetric and non-symmetric structure (Scheme 2). The reactions proceeded smoothly and afforded target bis(pyrimidines) **1f**–**n** in high yields. In the case of Bn-protected compound **4e**, containing no substituent at position *2*, it was necessary to significantly increase the time of hydrogenolysis, up to 96 h. This is probably connected to the coordination between the unhindered pyrimidine-moiety and Pd, leading to inactivation of the catalyst.

**Scheme 3:**
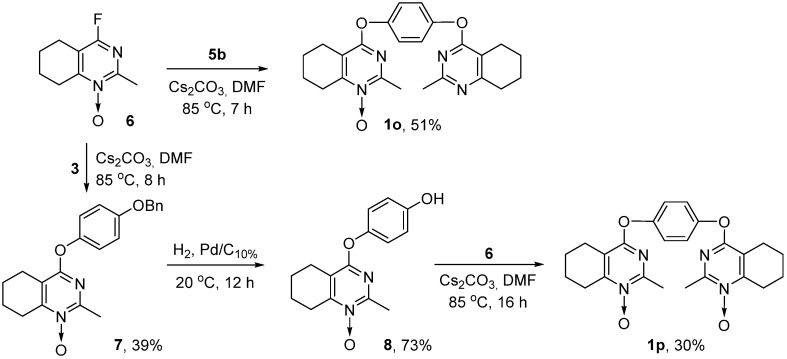
Synthesis of bis(pyrimidines) **1o**,**p**.

A similar synthetic scheme was carried out to obtain compounds **1o**,**p**, containing one and two pyrimidine *N*-oxide moieties, respectively (Scheme 3). 4-Fluoropyrimidine *N*-oxide **6**, readily available via heterocyclisation of 7-bromo-7-fluorobicyclo[4.1.0]heptane [42,43], was used as a starting compound. It should be noted that pyrimidine *N*-oxides **6**,**8** turned out to be less reactive and more labile in the S_N_Ar reaction conditions, compared with the corresponding pyrimidines, which made us increase the reaction time and caused a decrease in the preparative yields (Scheme 3).

### 2.2. Electrophysiological Evaluation

Compounds **1a**–**p** were examined in patch clamp experiments for their influence on the kainate-induced currents (Table 1), recorded for the freshly isolated Purkinje cells extracted from the rat cerebellum, as described earlier [28,29,37]. For most of the compounds, the potentiation of the AMPA receptor currents was observed in a wide concentration range (10^−12^–10^−6^ M), and had a bell-shaped concentration dependence with maximum potentiation (of up to 77%) at 10^−9^ M. On the other hand, compounds **1c** and **1j** demonstrated negative potentiation of the AMPA receptor in the same concentration range, with a decrease in current of approximately 30% at 10^−9^ M. The reference positive modulator of the AMPA receptor, cyclothiazide (CTZ), did not demonstrate any activity in concentrations lower than 10^−6^ M.

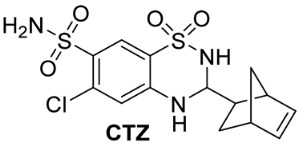



**Table 1:** The changes in the kainate-induced Purkinje cell currents upon treatment with compounds **1a**–**p**.

Compound	Number of Neurons *n*	Currents (%) for Various Concentrations of Compounds (M, Control = 100%)						
		10^−12^	10^−11^	10^−10^	10^−9^	10^−8^	10^−7^	10^−6^
**1a** [30]	7	108 ± 5	132 ± 5	143 ± 9	170 ± 11	123 ± 8	85 ± 6	78 ± 4
**1b** [30]	5	100 ± 2	117 ± 6	126 ± 8	155 ± 5	128 ± 7	100 ± 8	–
**1c** [30]	4	100 ± 2	84 ± 5	72 ± 6	82 ± 7	92 ± 4	98 ± 5	–
**1d** [30]	5	–	100 ± 2	108 ± 4	120 ± 4	125 ± 5	133 ± 6	145 ± 7
**1e** [30]	5	–	100 ± 2	100 ± 2	95 ± 4	96 ± 3	97 ± 2	96 ± 5
**1f**	4	101 ± 9	118 ± 10	147 ± 12	166 ± 12	157 ± 11	144 ± 12	122 ± 8
**1g**	3	100 ± 3	100 ± 3	108 ± 3	118 ± 4	129 ± 4	115 ± 4	101 ± 3
**1h**	5	100 ± 4	141 ± 9	149 ± 10	153 ± 9	129 ± 6	110 ± 5	100 ± 4
**1i**	5	103 ± 3	129 ± 9	141 ± 9	151 ± 12	134 ± 9	109 ± 4	102 ± 3
**1j**	6	94 ± 2	82 ± 3	76 ± 4	71 ± 4	64 ± 6	57 ± 6	52 ± 7
**1k**	5	105 ± 2	138 ± 5	149 ± 6	177 ± 6	163 ± 6	155 ± 7	117 ± 4
**1l**	4	111 ± 9	117 ± 9	124 ± 7	129 ± 6	138 ± 8	132 ± 8	124 ± 7
**1m**	4	120 ± 9	133 ± 9	129 ± 9	119 ± 9	117 ± 9	115 ± 9	102 ± 9
**1n**	3	100 ± 3	121 ± 4	129 ± 5	138 ± 6	136 ± 5	136 ± 4	112 ± 3
**1o**	5	115 ± 3	138 ± 9	147 ± 10	161 ± 9	160 ± 10	158 ± 11	116 ± 4
**1p**	4	117 ± 9	126 ± 11	128 ± 10	133 ± 11	125 ± 10	120 ± 11	102 ± 3
**CTZ**	8	-	-	-	-	-	100 ± 3	145 ± 11

**Figure 2:**
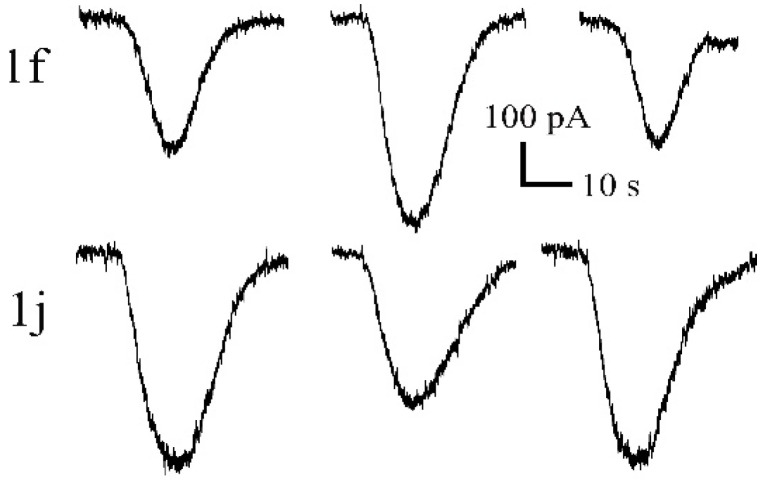
Kainate-induced currents at 10^−9^ M concentration for compounds **1f** and **1j** (left—control, center—after application of the compound, right—after wash-out).

As can be seen, in series **1f**–**p,** all the compounds except **1j** revealed the properties of a positive modulator, while **1j** acted as a negative modulator of the AMPA receptor (see Table 1, Figure 2 and Figure S1). The removal of a substituent in position *2* of the pyrimidine ring (**1f**) led to a decrease in potentiation in the picomolar concentration range, while in concentrations above 10^−8^ M the compound **1f** was more active than **1a**. The replacement of the six-membered rings of tetrahydroquinazoline moieties in compound **1a** by the five-membered rings (**1g**) led to a significant decrease in activity in almost the entire concentration range, while the compound with two seven-membered rings (**1h**) remained a potent PAM in concentrations of 10^−9^ M or lower; the same effect was observed after the replacement of the cycloalkane moieties by *tert*-butyl groups (**1i**). The best results, surpassing the ones previously obtained for **1a**, were found for compound **1k,** which activated kainate-induced currents by up to 77% and, in contrast to **1a**, did not act as a negative modulator in concentrations of 10^−7^–10^−6^ M.

### 2.3. Molecular Modeling

**Figure 3:**
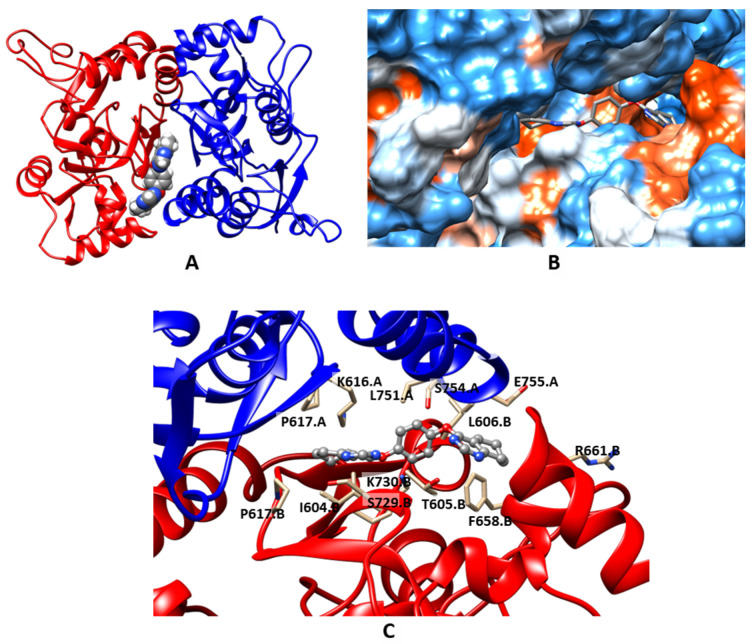
Binding mode of the PAM **1f**, refined using molecular dynamics simulation (150 ns). (**A**) General view of the dimeric ligand binding domain of AMPA receptor (GluA2) and location of the binding site. (**B**) Binding pockets on the protein molecular surface colored by local hydrophobicity (brown for hydrophobic and blue for hydrophilic). (**C**) Detailed view of the binding site. The ligand is represented by a grey ball-and-stick model; the amino acid residues located within 3 Å of it are represented by beige stick-models.

**Figure 4:**
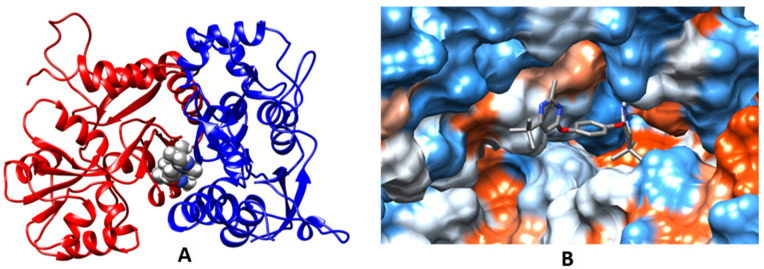

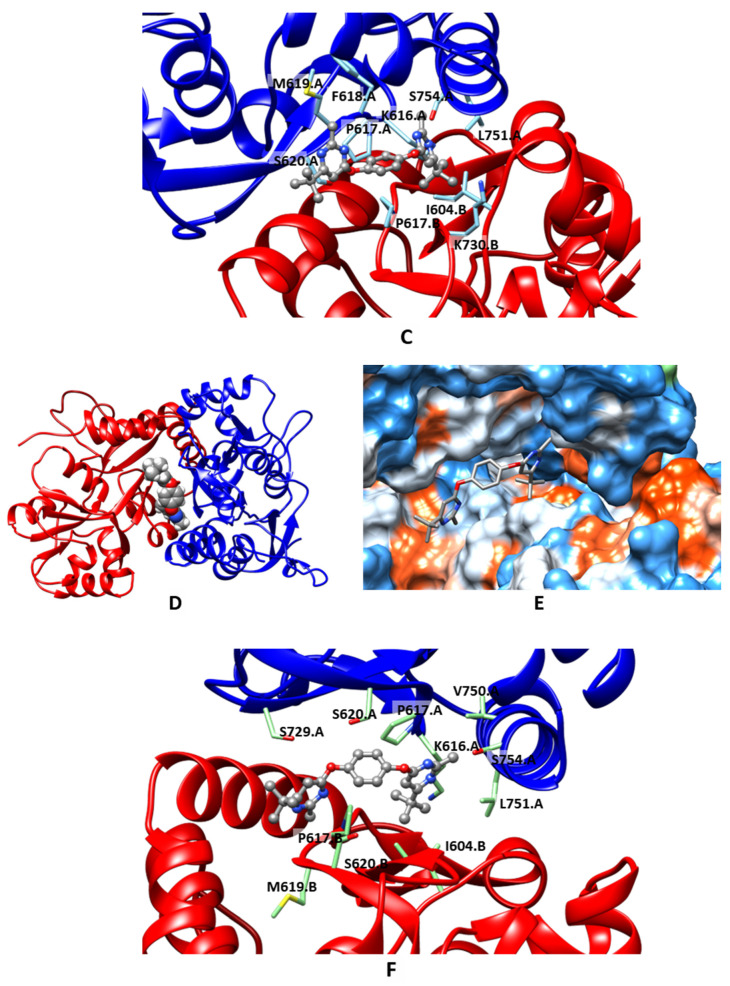
Lateral (**A**–**C**) and central (**D**–**F**) binding modes of the PAM **1i**, refined using molecular dynamics simulation (150 and 100 ns, respectively). (**A**,**D**) General view of the dimeric ligand binding domain of AMPA receptor (GluA2) and location of the binding site. (**B**,**E**) Binding pockets on the protein molecular surface colored by local hydrophobicity (brown for hydrophobic and blue for hydrophilic). (**C**,**F**) Detailed view of the binding site. The ligand is represented by a grey ball-and-stick model; the amino acid residues located within 3 Å of it are represented by cyan or mint stick-models.

**Figure 5:**
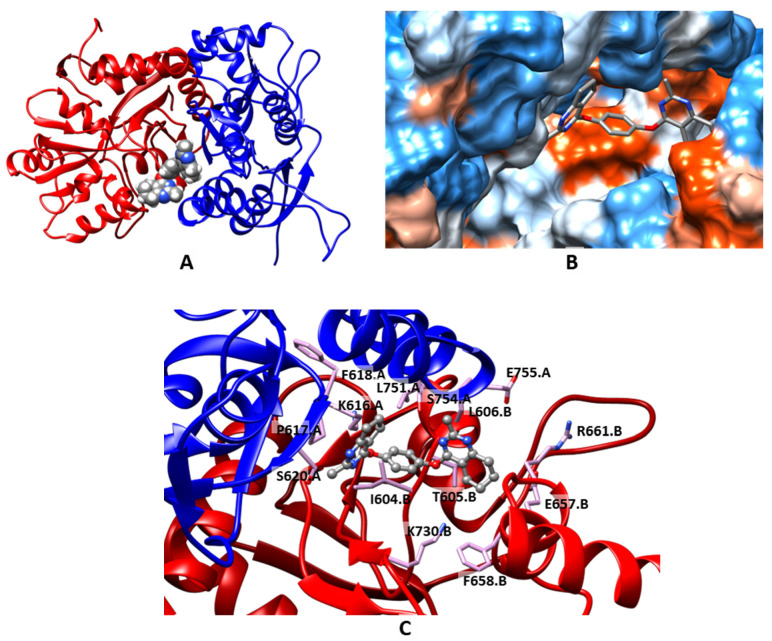
Binding mode of the NAM **1j**, refined using molecular dynamics simulation (150 ns). (**A**) General view of the dimeric ligand binding domain of AMPA receptor (GluA2) and location of the binding site. (**B**) Binding pockets in the protein molecular surface colored by local hydrophobicity (brown for hydrophobic and blue for hydrophilic). (**C**) Detailed view of the binding site. The ligand is represented by a grey ball-and-stick model, the amino acid residues located within 3 Å of it are represented by pink stick models.

**Figure 6:**
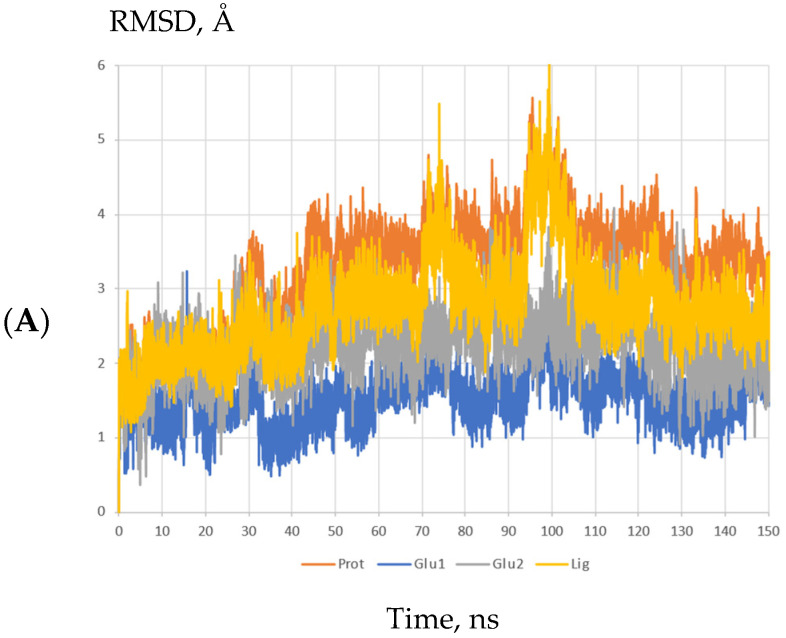

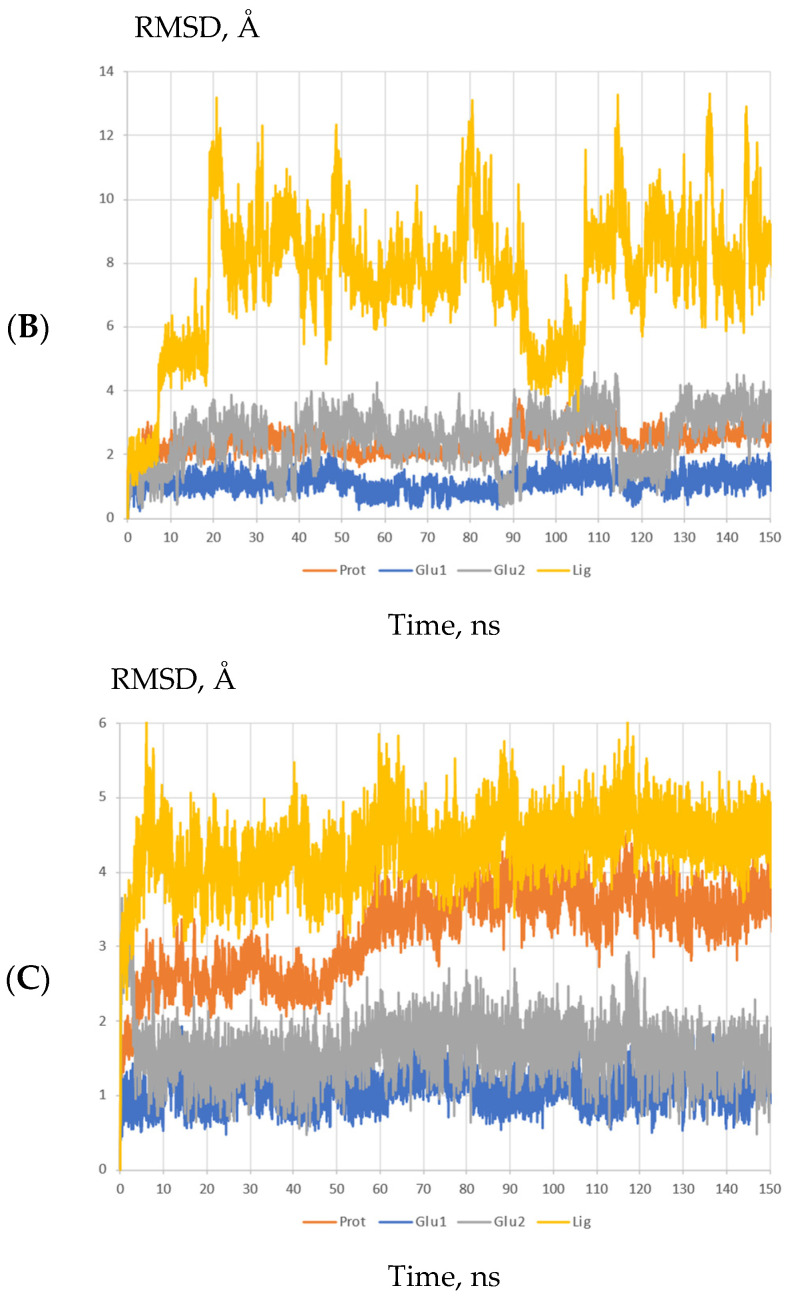
RMSD of the protein, glutamate, and ligand ((**A**)—**1f**, (**B**)—**1i**, (**C**)—**1j**) heavy atoms during molecular dynamics simulation of the modulator complex with the dimeric ligand binding domain of the GluA2 AMPA receptor.

In order to elucidate the probable mechanism of action of the allosteric modulators **1**, their interactions with the dimeric ligand binding domain (LBD) of the GluA2 AMPA receptor were modeled using a molecular docking and molecular dynamics simulation for the representative positive modulators **1f**, **1i** and the negative modulator **1j**. The compounds’ binding in the PAM binding site, at the interface between the ligand binding domains, was stable over the entire course of the simulation (150 ns). In a similar way to the other larger dimeric modulators [27,28,37,38], the modulator molecules attained an unsymmetric “lateral” position, occupying one of the side subpockets as well as part of the central subpocket of the symmetrical PAM binding site (Figure 3A,B, Figure 4A,B and Figure 5A,B). The binding was primarily stabilized by steric fit and hydrophobic interactions (Figure 3B,C, Figure 4B,C and Figure 5B,C) and a number of hydrogen bonds for compound **1i**. The plots of the root-mean-square deviations (RMSD) for the protein, glutamate, and ligand heavy atoms (Figure 6), plots of the protein and ligand solvent-accessible surface area (SASA), protein mass-weighted radius of gyration, and residue root-mean-square fluctuations (Figures S2–S4), as well as the visual inspection of the trajectories, confirm that system stability was retained over the entire course of the production simulations (150 ns), although the ligand positions were slightly adjusted, compared to the docking pose. Interestingly, compound **1i** is bound more loosely, and periodically shifts between this more frequently observed, “lateral” binding mode and the more short-lived “central” binding mode, wherein the modulator molecule is located in the central subpocket, similar to the “classic” PAMs (Figure 4D–F and Figure 6B). The binding free energies, estimated over the stable portion (last 20 ns) of the trajectories using the MM/GBSA approach, were −41.6 ± 0.3 kcal/mol for compound **1f**, −26.2 ± 0.4 kcal/mol for compound **1i** (probably due to looser binding), and –30.9 ± 0.3 kcal/mol for compound **1j**. Overall, these results indicate that compounds **1f** and **1i** can indeed act as positive AMPA receptor modulators that bind in the validated PAM binding site. Compared to the PAMs **1f** and **1i**, compound **1j** is more exposed to the solvent, while the separation between the LBD subunits is increased. This could explain the lack (or lower efficiency) of the positive modulator activity. On the other hand, similar to the tricyclic modulators [38], the negative modulator action of compound **1j** could potentially be mediated by competing interactions with the NAM binding sites at the interface between the LBD and the transmembrane domain (TMD). 

### 2.4. Prediction of ADMET, Physicochemical, and PAINS Profiles

Several ADMET and physicochemical properties for compounds **1a**–**p** were calculated (Table 2). They demonstrated high predicted values for intestinal absorption, enabling their oral administration. The predicted lipophilicities and aqueous solubilities were also appropriate for potential drug-like compounds, according to the commonly accepted rule of thumb. Due to the moderate predicted blood–brain barrier permeability, acceptable CNS bioavailability could be anticipated. Both parameters of the cardiac toxicity risk (hERG p*K_i_* and pIC_50_) (4.3–7.4 log units) were in the lower or medium parts of their possible ranges (3–9 log units), indicating a likely absence of hERG liabilities. The integral quantitative estimate of drug-likeness (QED) was greater than 0.4, confirming the favorable likely properties. The pan-assay interference compounds (PAINS) filter check did not identify any alerts. 

Overall, the predicted ADMET, physicochemical, and PAINS properties of the allosteric modulators **1a**–**p** were quite acceptable for the potential lead compounds at the early drug development stages, although additional checks and structure optimization would likely be required.

## 3. Materials and Methods

### 3.1. Chemistry

#### 3.1.1. General Remarks

^1^H and ^13^C NMR spectra were recorded on a 400 MHz spectrometer Agilent 400-MR (Agilent Technologies, Santa Clara, CA, USA), 400.0 and 100.6 MHz for ^1^H and ^13^C, respectively, at r.t. (room temperature) in CDCl_3_ if not stated othewise; chemical shifts δ were measured with reference to the solvent (CDCl_3_, δ_H_ = 7.26 ppm, δ_C_ = 77.16 ppm). When necessary, assignments of signals in NMR spectra were made using 2D techniques. Accurate mass measurements (HRMS) were obtained on a Bruker micrOTOF II (Bruker Daltonics, Billerica, MA, USA) with electrospray ionization (ESI). Analytical thin-layer chromatography was carried out with silica gel plates supported on aluminum (Macherey-Nagel, ALUGRAM^®^ Xtra SIL G/UV_254_); the detection was carried out using a UV lamp (254 nm). Column chromatography was performed on silica gel (Macherey-Nagel, Silica 60, 0.015–0.04 mm), Rf (retardation factors) and solvent systems are given for each compound. 4-Chloropyrimidines **2a** [44], **2b** [45], **2d** [46], **2e** [47], 4-fluoropyrimidine *N*-oxide **6** [43] and 2-methyl-3,5,6,7,8,9-hexahydro-4*H*-cyclohepta[*d*]pyrimidin-4-one [48] were obtained via the described methods. All other starting materials were commercially available. All reagents except commercial products of satisfactory quality were purified according to the literature procedures, prior to use.

#### 3.1.2. Synthesis of 4-Chloro-2-methyl-6,7,8,9-tetrahydro-5*H*-cyclohepta[*d*]pyrimidine (**2c**)

POCl_3_ (4.7 mL, 7.65 g, 50.0 mmol) was added dropwise to 2-methyl-3,5,6,7,8,9-hexahydro-4*H*-cyclohepta[*d*]pyrimidin-4-one (1.78 g, 10.0 mmol), stirring at 0 °C under argon. The reaction mixture was refluxed for 5 h, allowed to cool down to r.t., poured into the saturated icy solution of NaHCO_3_ (50 mL) and extracted with DCM (3 × 30 mL). Combined organic layers were washed with water (3 × 30 mL) and dried over MgSO_4_; the solvent was evaporated under reduced pressure. The product was isolated via preparative column chromatography (SiO_2_). 

Yield 94% (197 mg). Yellowish oil, Rf = 0.7 (light petrol-EtOAc 4:1).

^1^H NMR (CDCl_3_, δ, ppm): 1.46–1.65 (m, 4H, 2CH_2_), 1.71–1.85 (m, 2H, CH_2_), 2.51 (s, 3H, CH_3_), 2.76–2.95 (m, 4H, 2CH_2_);

^13^C NMR (CDCl_3_, δ, ppm): 25.2 (CH_3_), 25.6 (CH_2_), 26.3 (CH_2_), 28.9 (CH_2_), 31.9 (CH_2_), 38.8 (CH_2_), 130.3 (C(4a)), 159.1 (C(4)), 165.0 (C(2)), 173.3 (C(9a)). 

HRMS (ESI^+^, *m*/*z*): calcd. for C_10_H_13_ClN_2_ [M + H]^+^ 197.0840, 199.0811; found 197.0844, 199.0816. 

#### 3.1.3. Synthesis of 4-(4-(Benzyloxy)phenoxy)pyrimidines **4a**–**e**,**7** (General Method)

The mixture of corresponding 4-halogenopyrimidine **2a**–**e** or **6** (1.0 mmol) and Cs_2_CO_3_ (652 mg, 2.0 mmol) in absolute DMF (10 mL) was stirred for 10 min at r.t. under argon. 4-(Benzyloxy)phenol (400 mg, 2.0 mmol) was added. The reaction mixture was stirred at 85 °C for 4–8 h, allowed to cool down to r.t., quenched with an equal volume of water and extracted with EtOAc (3 × 10 mL). Combined organic layers were washed with brine (3 × 10 mL) and dried over MgSO_4_; the solvent was evaporated under reduced pressure. The products were isolated via preparative column chromatography (SiO_2_).

##### 4-(4-(Benzyloxy)phenoxy)-2-methyl-5,6,7,8-tetrahydroquinazoline (**4a**)

Yield 82% (283 mg). White solid, m.p. (melting point) 165–166 °C, Rf = 0.3 (light petrol-EtOAc 2:1).

^1^H NMR (CDCl_3_, δ, ppm): 1.77˗1.96 (m, 4H, 2CH_2_), 2.46 (s, 3H, CH_3_), 2.69–2.74 (m, 2H, CH_2_), 2.78–2.83 (m, 2H, CH_2_), 5.05 (s, 2H, CH_2_O), 6.95–7.03 (m, 2H, 2CH, Ar), 7.04–7.11 (m, 2H, 2CH, Ar), 7.29–7.49 (m, 5H, 5CH, Ph);

^13^C NMR (CDCl_3_, δ, ppm): 21.7 (CH_2_), 22.0 (CH_2_), 22.3 (CH_2_), 25.6 (CH_3_), 31.8 (CH_2_), 70.4 (CH_2_O), 113.8 (C(4a), THQ), 115.3 (2CH, Ar), 122.5 (2CH, Ar), 127.5 (2CH, Ph), 128.0 (CH, Ph), 128.6 (2CH, Ph), 136.9 (C, Ph), 146.6 (C, Ar), 155.8 (C, Ar), 164.1 (C, THQ), 166.2 (C, THQ), 167.1 (C, THQ). 

HRMS (ESI^+^, *m*/*z*): calcd. for C_22_H_22_N_2_O_2_ [M + H]^+^ 347.1754, found 347.1751. 

##### 4-(4-(Benzyloxy)phenoxy)-2-methyl-6,7-dihydro-5*H*-cyclopenta[*d*]pyrimidine (**4b**)

Yield 90% (299 mg). White solid, m.p. 107–110 °C, Rf = 0.2 (light petrol-EtOAc 2:1).

^1^H NMR (CDCl_3_, δ, ppm): 2.05–2.19 (m, 2H, CH_2_), 2.52 (s, 3H, CH_3_), 2.84–2.90 (m, 2H, CH_2_), 2.93–2.98 (m, 2H, CH_2_), 5.05 (2H, s, CH_2_O), 6.92–7.02 (m, 2H, 2CH, Ar), 7.03–7.14 (m, 2H, 2CH, Ar), 7.28–7.50 (m, 5H, 5CH, Ph);

^13^C NMR (CDCl_3_, δ, ppm): 22.0 (CH_2_), 25.6 (CH_3_), 26.9 (CH_2_), 34.3 (CH_2_), 70.5 (CH_2_O), 115.4 (2CH, Ar), 117.0 (C(4a), cy-pent-Pyr), 122.4 (2CH, Ar), 127.6 (2CH, Ph), 128.1 (CH, Ph), 128.6 (2CH, Ph), 137.0 (C, Ph), 146.5 (C, Ar), 156.0 (C, Ar), 165.4 (C(4), cy-pent-Pyr), 166.8 (C(2), cy-pent-Pyr), 176.6 (C(7a), cy-pent-Pyr).

HRMS (ESI^+^, *m*/*z*): calcd. for C_21_H_20_N_2_O_2_ [M + H]^+^ 333.1598, found 333.1596.

##### 4-(4-(Benzyloxy)phenoxy)-2-methyl-6,7,8,9-tetrahydro-5*H*-cyclohepta[*d*]pyrimidine (**4c**)

Yield 75% (270 mg). Beige solid, m.p. 121–123 °C, Rf = 0.8 (CHCl_3_-MeOH 50:1).

^1^H NMR (CDCl_3_, δ, ppm): 1.59–1.77 (m, 4H, C(6)H_2,_ C(8)H_2_), 1.82–1.96 (m, 2H, C(7)H_2_), 2.46 (s, 3H, CH_3_), 2.88–2.93 (m, 2H, C(5)H_2_), 2.94–3.01 (m, 2H, C(9)H_2_), 5.04 (s, 2H, CH_2_O), 6.92–7.01 (m, 2H, 2CH, Ar), 7.01–7.10 (m, 2H, 2CH, Ar), 7.29–7.49 (m, 5H, 5CH, Ph);

^13^C NMR (CDCl_3_, δ, ppm): 24.3 (C(5)H_2_), 25.5 (CH_3_), 25.8 (C(8)H_2_), 26.9 (C(6)H_2_), 32.4 (C(7)H_2_), 38.4 (C(9)H_2_), 70.3 (CH_2_O), 115.3 (2CH, Ar), 118.5 (C(4a), cy-hept-Pyr), 122.3 (2CH, Ar), 127.5 (2CH, Ph), 127.9 (CH, Ph), 128.5 (2CH, Ph), 136.9 (C, Ph), 147.0 (C, Ar), 155.7 (C, Ar), 164.1 (C(2), cy-hept-Pyr), 166.3 (C(4), cy-hept-Pyr), 172.6 (C(9a), cy-hept-Pyr).

HRMS (ESI^+^, *m*/*z*): calcd. for C_23_H_24_N_2_O_2_ [M + H]^+^ 361.1911, found 361.1903.

##### 4-(4-(Benzyloxy)phenoxy)-6-(*tert*-butyl)-2-methylpyrimidine (**4d**)

Yield 80% (278 mg). White solid, m.p. 101–102 °C, Rf = 0.6 (light petrol-EtOAc 5:1).

^1^H NMR (CDCl_3_, δ, ppm): 1.33 (s, 9H, 3CH_3_, *t*-Bu), 2.59 (s, 3H, CH_3_), 5.07 (s, 2H, CH_2_O), 6.58 (s, 1H, CH, Pyr), 6.97–7.05 (m, 2H, 2CH, Ar), 7.05–7.14 (m, 2H, 2CH, Ar), 7.32–7.37 (m, 1H, Ph), 7.38–7.43 (m, 2H, Ph), 7.44–7.48 (m, 2H, Ph);

^13^C NMR (CDCl_3_, δ, ppm): 26.1 (CH_3_), 29.3 (3CH_3_), 37.4 (C, *t*-Bu), 70.4 (CH_2_O), 99.0 (CH, Pyr), 115.7 (2CH, Ar), 122.4 (2CH, Ar), 127.5 (2CH, Ph), 128.1 (CH, Ph), 128.6 (2CH, Ph), 136.9 (C, Ph), 146.4 (C, Ar), 156.1 (C, Ar), 167.6 (C, Pyr), 170.4 (C, Pyr), 180.0 (C, Pyr).

HRMS (ESI^+^, *m*/*z*): calcd. for C_22_H_24_N_2_O_2_ [M + H]^+^ 349.1911, found 349.1910. 

##### 4-(4-(Benzyloxy)phenoxy)-5,6,7,8-tetrahydroquinazoline (**4e**)

Yield 72% (239 mg). White solid, m.p. 87–91 °C, Rf = 0.6 (CHCl_3_-MeOH 10:1).

^1^H NMR (CDCl_3_, δ, ppm): 1.82–1.98 (m, 4H, 2CH_2_, cy-Hex), 2.74–2.80 (m, 2H, CH_2_, cy-Hex), 2.82–2.87 (m, 2H, CH_2_, cy-Hex), 5.07 (s, 2H, CH_2_O), 7.96–7.10 (m, 4H, 4CH, Ar), 7.31–7.36 (m, 1H, Ph), 7.37–7.42 (m, 2H, Ph), 7.42–7.46 (m, 2H, Ph), 8.48 (s, 1H, CH, THQ); 

^13^C NMR (CDCl_3_, δ, ppm): 21.84 (CH_2_), 21.90 (CH_2_), 22.1 (CH_2_), 31.8 (CH_2_), 70.4 (CH_2_O), 115.6 (2CH, Ar), 117.3 (C(4a), THQ), 122.6 (2CH, Ar), 127.5 (2CH, Ph), 128.0 (CH, Ph), 128.6 (2CH, Ph), 136.9 (C, Ph), 146.2 (C, Ar), 154.5 (CH, THQ), 156.2 (C, Ar), 166.5 (C, THQ), 167.4 (C, THQ). 

HRMS (ESI^+^, *m*/*z*): calcd. for C_21_H_20_N_2_O_2_ [M + H]^+^ 333.1598, found 333.1596.

##### 4-(4-(Benzyloxy)phenoxy)-2-methyl-5,6,7,8-tetrahydroquinazoline 1-Oxide (**7**)

Yield 39% (141 mg). Yellowish solid, m.p. 133–134 °C, Rf = 0.3 (DCM-MeOH 20:1). 

^1^H NMR (CDCl_3_, δ, ppm): 1.74–1.86 (m, 2H, CH_2_), 1.85–1.98 (m, 2H, CH_2_), 2.55 (s, 3H, CH_3_), 2.72–2.78 (m, 2H, CH_2_), 2.93–2.99 (m, 2H, CH_2_), 5.07 (s, 2H, CH_2_O), 6.93–7.10 (m, 4H, 4CH, Ar), 7.31–7.36 (m, 1H, Ph), 7.37–7.42 (m, 2H, Ph), 7.42–7.46 (m, 2H, Ph); 

^13^C NMR (CDCl_3_, δ, ppm): 20.0 (CH_2_), 20.7 (CH_2_), 21.1 (CH_2_), 22.2 (CH_2_), 25.1 (CH_3_), 70.5 (CH_2_O), 115.6 (2CH, Ar), 117.4 (C(4a), THQ), 122.4 (2CH, Ar), 127.6 (2CH, Ph), 128.2 (CH, Ph), 128.7 (2CH, Ph), 136.9 (C, Ph), 146.4 (C, Ar), 154.9 (C, Ar), 156.2 (C, THQ), 156.4 (C, THQ), 157.4 (C, THQ).

HRMS (ESI^+^, *m*/*z*): calcd. for C_22_H_22_N_2_O_3_ [M + H]^+^ 363.1703, found 363.1697. 

#### 3.1.4. Synthesis of 4-((Pyrimidin-4-yl)oxy)phenols **5a**–**e**,**8** (General Method)

To a degassed (argon, 20 min) solution of a corresponding 4-(4-(benzyloxy)phenoxy)pyrimidine **4a**–**e** or **7** (1 mmol) in EtOAc (4 mL) and methanol (20 mL), Pd/C_10%_ (60 mg) was added. The reaction mixture was vigorously stirred under H_2_ (current 8.3 L/h) for 4–96 h; the mixture of solvents was added as they evaporated. Completion of the reaction was monitored via thin-layer chromatography. The catalyst was removed via filtration; the solvent was evaporated under reduced pressure. The products were isolated via preparative column chromatography (SiO_2_) (**5e**,**8**) or used without additional purification (**5a**–**d**).

##### 4-((2-Methyl-5,6,7,8-tetrahydroquinazolin-4-yl)oxy)phenol (**5a**) 

Yield 98% (251 mg). White solid, m.p. 177–179 °C.

^1^H NMR (CDCl_3_, δ, ppm): 1.73˗1.96 (m, 4H, 2CH_2_), 2.47 (s, 3H, CH_3_), 2.69–2.75 (m, 2H, CH_2_), 2.78–2.94 (m, 2H, CH_2_), 6.69–6.85 (m, 2H, 2CH), 6.85–6.98 (m, 2H, 2CH);

^13^C NMR (CDCl_3_, δ, ppm): 21.8 (CH_2_), 22.0 (CH_2_), 22.2 (CH_2_), 25.1 (CH_3_), 31.4 (CH_2_), 114.5 (C(4a), THQ), 116.4 (2CH), 122.5 (2CH), 145.6 (C, Ar), 154.1 (C, Ar), 164.2 (C, THQ), 166.2 (C, THQ), 167.6 (C, THQ).

HRMS (ESI^+^, *m*/*z*): calcd. for C_15_H_16_N_2_O_2_ [M + H]^+^ 257.1285, found 257.1285. 

##### 4-((2-Methyl-6,7-dihydro-5*H*-cyclopenta[*d*]pyrimidin-4-yl)oxy)phenol (**5b**) 

Yield 93% (225 mg). White solid, m.p. 168–173 °C.

^1^H NMR (CDCl_3_, δ, ppm): 2.06–2.22 (m, 2H, CH_2_), 2.54 (s, 3H, CH_3_), 2.85–2.91 (m, 2H, CH_2_), 2.94–3.00 (m, 2H, CH_2_), 6.74–6.84 (m, 2H, 2CH), 6.92–7.01 (m, 2H, 2CH);

^13^C NMR (CDCl_3_, δ, ppm): 22.1 (CH_2_), 25.3 (CH_3_), 27.0 (CH_2_), 34.2 (CH_2_), 116.4 (2CH), 117.7 (C(4a), cy-pent-Pyr), 122.5 (2CH), 145.8 (C, Ar), 153.9 (C, Ar), 165.8 (C, cy-pent-Pyr), 166.8 (C, cy-pent-Pyr), 176.5 (C, cy-pent-Pyr).

HRMS (ESI^+^, *m*/*z*): calcd. for C_14_H_14_N_2_O_2_ [M + H]^+^ 243.1128, found 243.1128.

##### 4-((2-Methyl-6,7,8,9-tetrahydro-5*H*-cyclohepta[*d*]pyrimidin-4-yl)oxy)phenol (**5c**)

Yield 70% (189 mg). White solid, m.p. 156–158 °C.

^1^H NMR (CDCl_3_, δ, ppm): 1.63–1.74 (m, 4H, C(6)H_2_, C(8)H_2_), 1.86–1.94 (m, 2H, C(7)H_2_), 2.47 (s, 3H, CH_3_), 2.88–2.93 (m, 2H, C(5)H_2_), 2.94–2.99 (m, 2H, C(9)H_2_), 6.71–6.79 (m, 2H, 2CH), 6.86–6.97 (m, 2H, 2CH);

^13^C NMR (CDCl_3_, δ, ppm): 24.4 (C(5)H_2_), 25.2 (CH_3_), 25.9 (C(8)H_2_), 27.0 (C(6)H_2_), 32.5 (C(7)H_2_), 38.1 (C(9)H_2_), 116.5 (2CH), 119.3 (C(4a), cy-hept-Pyr), 122.4 (2CH), 146.2 (C, Ar), 153.7 (C, Ar), 164.3 (C(2), cy-hept-Pyr), 166.8 (C(4), cy-hept-Pyr), 172.7 (C(9a), cy-hept-Pyr).

HRMS (ESI^+^, *m*/*z*): calcd. for C_16_H_18_N_2_O_2_ [M + H]^+^ 271.1441, found 271.1442.

##### 4-((6-(*tert*-Butyl)-2-methylpyrimidin-4-yl)oxy)phenol (**5d**) 

Yield 92% (237 mg). Beige solid, m.p. 115–119 °C with decomposition.

^1^H NMR (CDCl_3_, δ, ppm): 1.29 (s, 9H, 3CH_3_, *t*-Bu), 2.58 (s, 3H, CH_3_), 6.57 (s, 1H, CH, Pyr), 6.78–6.86 (m, 2H, 2CH, Ar), 6.92–7.01 (m, 2H, 2CH, Ar);

^13^C NMR (CDCl_3_+CD_3_OD, δ, ppm): 25.4 (CH_3_), 29.1 (3CH_3_, *t*-Bu), 37.4 (C, *t*-Bu), 98.7 (CH, Pyr), 116.3 (2CH, Ar), 122.1 (2CH, Ar), 145.0 (C, Ar), 154.5 (C, Ar), 167.6 (C, Pyr), 170.8 (C, Pyr), 180.4 (C, Pyr).

HRMS (ESI^+^, *m*/*z*): calcd. for C_15_H_18_N_2_O_2_ [M + H]^+^ 259.1441, found 259.1450.

##### 4-((5,6,7,8-Tetrahydroquinazolin-4-yl)oxy)phenol (**5e**)

Yield 61% (148 mg). White solid, m.p. 193–195 °C, Rf = 0.6 (CHCl_3_-MeOH 10:1).

^1^H NMR (CDCl_3_, δ, ppm): 1.76–1.98 (m, 4H, 2CH_2_), 2.74–2.79 (m, 2H, CH_2_), 2.84–2.89 (m, 2H, CH_2_), 6.71–6.87 (m, 2H, 2CH, Ar), 6.89–7.01 (m, 2H, 2CH, Ar), 7.86 (br.s, H, OH), 8.50 (s, 1H, CH, Pyr); 

^13^C NMR (CDCl_3_, δ, ppm): 21.9 (CH_2_), 22.0 (CH_2_), 22.1 (CH_2_), 31.6 (CH_2_), 116.7 (2CH, Ar), 117.8 (C(4a), THQ), 122.7 (2CH, Ar), 145.4 (C, Ar), 154.2 (C, Ar), 154.6 (CH, THQ), 166.5 (C, THQ), 167.8 (C, THQ).

HRMS (ESI^+^, *m*/*z*): calcd. for C_14_H_14_N_2_O_2_ [M + H]^+^ 243.1128, found 243.1130.

##### 4-[(2-Methyl-1-oxido-5,6,7,8-tetrahydroquinazolin-4-yl)oxy]phenol (**8**) [30]

Yield 73% (199 mg). White solid, m.p. 245–248 °C with decomposition, Rf = 0.1 (DCM-MeOH 20:1).

^1^H NMR (CDCl_3_+CD_3_OD, δ, ppm): 1.71˗1.81 (m, 2H, CH_2_), 1.81˗1.93 (m, 2H, CH_2_), 2.48 (s, 3H, CH_3_), 2.66–2.71 (m, 2H, CH_2_), 2.84–2.89 (m, 2H, CH_2_), 6.75–6.82 (m, 2H, 2CH), 6.85–6.90 (m, 2H, 2CH).

#### 3.1.5. Synthesis of Bis(pyrimidines) **1a**–**p** (General Method)

The mixture of corresponding 4-((pyrimidin-4-yl)oxy)phenol **5a**–**e** or **8** (1.0 mmol) and Cs_2_CO_3_ (652 mg, 2.0 mmol) in absolute DMF (10 mL) was stirred for 10 min at r.t., under argon. 4-Halogenopyrimidine **2a**–**e** or **6** (2.0 mmol) was added. The reaction mixture was stirred at 85 °C for 6–16 h, allowed to cool down to r.t., quenched with an equal volume of water and extracted with EtOAc (3 × 10 mL). Combined organic layers were washed with brine (3 × 10 mL) and dried over MgSO_4_; the solvent was evaporated under reduced pressure. The products were isolated via preparative column chromatography (SiO_2_).

##### 1,4-Bis((2-methyl-5,6,7,8-tetrahydroquinazolin-4-yl)oxy)benzene (**1a**) [30]

Yield 90% (362 mg). White solid, m.p. 133–135 °C, Rf = 0.6 (CHCl_3_-MeOH 10:1). 

^1^H NMR (CDCl_3_, δ, ppm): 1.78–1.95 (m, 8H, 2CH_2_, 2CH_2_), 2.45 (s, 6H, 2CH_3_), 2.70–2.75 (m, 4H, 2CH_2_), 2.77–2.83 (m, 4H, 2CH_2_), 7.15 (s, 4H, 4CH, Ar). 

##### 1,4-Bis((5,6,7,8-tetrahydroquinazolin-4-yl)oxy)benzene (**1f**)

Yield 89% (333 mg). White solid, m.p. 178–182 °C, Rf = 0.6 (CHCl_3_-MeOH 10:1). 

^1^H NMR (CDCl_3_, δ, ppm): 1.81–1.96 (m, 8H, 2C(6)H_2_, 2C(7)H_2_), 2.76–2.81 (m, 4H, 2C(5)H_2_), 2.83–2.88 (m, 4H, 2C(8)H_2_), 7.19 (s, 4H, 4CH, Ar), 8.48 (s, 2H, 2CH, THQ);

^13^C NMR (CDCl_3_, δ, ppm): 21.95 (2CH_2_), 22.01 (2CH_2_), 22.2 (2CH_2_), 32.0 (2C(8)H_2_), 117.6 (2C(4a), THQ), 122.9 (4CH, Ar), 149.9 (2C, Ar), 154.9 (2CH, THQ), 167.0 (2C(4), THQ), 167.2 (2C(8a), THQ).

HRMS (ESI^+^, *m*/*z*): calcd. for C_22_H_22_N_4_O_2_ [M + H]^+^ 375.1816, found 375.1812.

##### 1,4-Bis((2-methyl-6,7-dihydro-5*H*-cyclopenta[*d*]pyrimidin-4-yl)oxy)benzene (**1g**)

Yield 72% (270 mg). White solid, m.p. 137–141 °C, Rf = 0.3 (light petrol-EtOAc-MeOH 3:1:0.5).

^1^H NMR (CDCl_3_, δ, ppm): 2.10–2.21 (m, 4H, 2CH_2_), 2.53 (s, 6H, 2CH_3_), 2.89–2.94 (m, 4H, 2CH_2_), 2.95–3.00 (m, 4H, 2CH_2_), 7.18 (s, 4H, 4CH);

^13^C NMR (CDCl_3_, δ, ppm): 22.1 (2CH_2_), 25.6 (2CH_3_), 26.9 (2CH_2_), 34.5 (2CH_2_), 117.4 (2C(4a), cy-pent-Pyr), 122.5 (4CH, Ar), 149.8 (2C, Ar), 165.3 (2C, cy-pent-Pyr), 166.9 (2C, cy-pent-Pyr), 177.1 (2C, cy-pent-Pyr).

HRMS (ESI^+^, *m*/*z*): calcd. for C_22_H_22_N_4_O_2_ [M + H]^+^ 375.1816, found 375.1815.

##### 1,4-Bis((2-methyl-6,7,8,9-tetrahydro-5*H*-cyclohepta[*d*]pyrimidin-4-yl)oxy)benzene (**1h**)

Yield 78% (335 mg). White solid, m.p. 189–190 °C, Rf = 0.2 (light petrol-EtOAc 4:1).

^1^H NMR (CDCl_3_, δ, ppm): 1.53–1.76 (m, 8H, 2C(6)H_2_, 2C(8)H_2_), 1.80–1.94 (m, 4H, 2C(7)H_2_), 2.43 (s, 6H, 2CH_3_), 2.79–3.01 (m, 8H, 2C(5)H_2_, 2C(9)H_2_), 7.10 (br.s, 4H, 4CH);

^13^C NMR (CDCl_3_, δ, ppm): 24.4 (2C(5)H_2_), 25.5 (2CH_3_), 25.8 (2C(8)H_2_), 27.0 (2C(6)H_2_), 32.4 (2C(7)H_2_), 38.6 (2C(9)H_2_), 118.8 (2C(4a), cy-hept-Pyr), 122.1 (4CH, Ar), 150.0 (2C, Ar), 164.3 (2C(2), cy-hept-Pyr), 166.1 (2C(4), cy-hept-Pyr), 173.0 (2C(9a), cy-hept-Pyr).

HRMS (ESI^+^, *m*/*z*): calcd. for C_26_H_30_N_4_O_2_ [M + H]^+^ 431.2442, found 431.2435.

##### 1,4-Bis((6-(*tert*-butyl)-2-methylpyrimidin-4-yl)oxy)benzene (**1i**) 

Yield 75% (300 mg). White solid, m.p. 201–203 °C, Rf = 0.2 (CHCl_3_-MeOH 50:1).

^1^H NMR (CDCl_3_, δ, ppm): 1.31 (s, 18H, 6CH_3_, *t*-Bu), 2.56 (s, 6H, 2CH_3_), 6.62 (s, 2H, CH, Pyr), 7.19 (s, 4H, 4CH, Ar);

^13^C NMR (CDCl_3_, δ, ppm): 26.0 (2CH_3_), 29.3 (6CH_3_, *t*-Bu), 37.4 (2C, *t*-Bu), 99.5 (2CH, Pyr), 122.6 (4CH, Ar), 149.8 (2C, Ar), 167.3 (2C(2), Pyr), 170.0 (2C(4), Pyr), 180.3 (2C(6), Pyr).

HRMS (ESI^+^, *m*/*z*): calcd. for C_24_H_30_N_4_O_2_ [M + H]^+^ 407.2442, found 407.2436.

##### 2-Methyl-4-(4-((2-methyl-6,7-dihydro-5*H*-cyclopenta[*d*]pyrimidin-4-yl)oxy)phenoxy)-5,6,7,8-tetrahydroquinazoline (**1j**)

Yield 86% (333 mg). White solid, m.p. 223–225 °C, Rf = 0.1 (light petrol-EtOAc 2:1).

^1^H NMR (CDCl_3_, δ, ppm): 1.79˗1.94 (m, 4H, C(6)H_2_, C(7)H_2_, THQ), 2.06–2.22 (m, 2H, C(6)H_2_, cy-pent-Pyr), 2.45 (s, 3H, CH_3_, THQ), 2.52 (s, 3H, CH_3_, cy-pent-Pyr), 2.70–2.75 (m, 2H, C(5)H_2_, THQ), 2.78–2.83 (m, 2H, C(8)H_2_, THQ), 2.88–2.93 (m, 2H, C(5)H_2_, cy-pent-Pyr), 2.95–3.00 (m, 2H, C(7)H_2_, cy-pent-Pyr), 7.08–7.24 (m, 4H, 4CH);

^13^C NMR (CDCl_3_, δ, ppm): 21.8 (C(5)H_2_, THQ), 22.09 (C(6)H_2_, cy-pent-Pyr), 22.14 (C(6)H_2_, THQ), 22.4 (C(7)H_2_, THQ), 25.60 (CH_3_), 25.61 (CH_3_), 26.9 (C(5)H_2_, cy-pent-Pyr), 32.0 (C(8)H_2_, THQ), 34.4 (C(7)H_2_, cy-pent-Pyr), 114.1 (C(4a), THQ), 117.3 (C(4a), cy-pent-Pyr), 122.4 (2CH), 122.6 (2CH), 149.7 (C, Ar), 149.9 (C, Ar), 164.2 (C(2), THQ), 165.3 (C(4), cy-pent-Pyr), 166.8 (C(8a), THQ), 166.9 (C(4), THQ), 167.0 (C(2), cy-pent-Pyr), 177.0 (C(7a), cy-pent-Pyr).

HRMS (ESI^+^, *m*/*z*): calcd. for C_23_H_24_N_4_O_2_ [M + H]^+^ 389.1972, found 389.1965.

##### 2-Methyl-4-(4-((2-methyl-5,6,7,8-tetrahydroquinazolin-4-yl)oxy)phenoxy)-6,7,8,9-tetrahydro-5*H*-cyclohepta[*d*]pyrimidine (**1k**)

Yield 75% (312 mg). White solid, m.p. 185–186 °C, Rf = 0.3 (EtOAc).

^1^H NMR (CDCl_3_, δ, ppm): 1.56–1.73 (m, 4H, C(6)H_2_, C(8)H_2_, cy-hept-Pyr), 1.75–1.94 (m, 6H, C(6)H_2_, C(8)H_2_, THQ; C(7)H_2_, cy-hept-Pyr), 2.42 (br.s, 6H, 2CH_3_), 2.66–2.71 (m, 2H, C(5)H_2_, THQ), 2.74–2.79 (m, 2H, C(8)H_2_, THQ), 2.84–2.97 (m, 4H, C(5)H_2_, C(9)H_2_, cy-hept-Pyr), 6.99–7.23 m (4H, 4CH, Ar);

^13^C NMR (CDCl_3_, δ, ppm): 21.7 (C(5)H_2_, THQ), 22.1 (C(6)H_2_, THQ), 22.3 (C(7)H_2_, THQ), 24.4 (CH_2_, C(5)H_2_, cy-hept-Pyr), 25.50 (CH_3_), 25.52 (CH_3_), 25.8 (C(8)H_2_, cy-hept-Pyr), 27.0 (C(6)H_2_, cy-hept-Pyr), 31.9 (C(8)H_2_, THQ), 32.4 (C(7)H_2_, cy-hept-Pyr), 38.6 (C(9)H_2_, cy-hept-Pyr), 113.9 (C(4a), THQ), 118.8 (C(4a), cy-hept-Pyr), 122.2 (2CH, Ar), 122.4 (2CH, Ar), 149.5 (C, Ar), 150.2 (C, Ar), 164.1 (C(2), THQ), 164.3 (C(2), cy-hept-Pyr), 166.1 (C(4), cy-hept-Pyr), 166.6 (C(8a), THQ), 166.9 (C(4), THQ), 173.0 (C(9a), cy-hept-Pyr). 

HRMS (ESI^+^, *m*/*z*): calcd. for C_25_H_28_N_4_O_2_ [M + H]^+^ 417.2285, found 417.2277.

##### 4-(4-((6-(*tert*-Butyl)-2-methylpyrimidin-4-yl)oxy)phenoxy)-2-methyl-5,6,7,8-tetrahydroquinazoline (**1l**)

Yield 63% (254 mg). White solid, m.p. 153–154 °C, Rf = 0.1 (light petrol-EtOAc 4:1).

^1^H NMR (CDCl_3_, δ, ppm): 1.29 (s, 9H, 3CH_3_, *t*-Bu), 1.78–1.94 (m, 4H, C(6)H_2_, C(7)H_2_), 2.45 (s, 3H, CH_3_, THQ), 2.55 (s, 3H, CH_3_*,* Pyr), 2.70–2.75 (m, 2H, C(5)H_2_), 2.77–2.82 (m, 2H, C(8)H_2_), 6.60 (s, 1H, CH, Pyr), 7.14–7.17 (m, 4H, 4CH, Ar).

^13^C NMR (CDCl_3_, δ, ppm): 21.8 (C(5)H_2_), 22.1 (CH_2_), 22.4 (CH_2_), 25.6 (CH_3_, THQ), 26.1 (CH_3_*,* Pyr), 29.4 (3CH_3_, *t*-Bu), 32.0 (C(8)H_2_), 37.5 (C, *t*-Bu), 99.5 (CH*,* Pyr), 114.1 (C(4a), THQ), 122.4 (2CH, Ar), 122.9 (2CH, Ar), 149.6 (C, Ar), 150.1 (C, Ar), 164.2 (C(2), THQ), 166.8 (C, THQ), 166.9 (C, THQ), 167.7 (C(2)*,* Pyr), 170.2 (C(4)*,* Pyr), 180.3 (C(6) Pyr).

HRMS (ESI^+^, *m*/*z*): calcd. for C_24_H_28_N_4_O_2_ [M + H]^+^ 405.2285, found 405.2288. 

##### 4-(4-((6-(*tert*-Butyl)-2-methylpyrimidin-4-yl)oxy)phenoxy)-2-methyl-6,7-dihydro-5*H*-cyclopenta[*d*]pyrimidine (**1m**)

Yield 64% (249 mg). White solid, m.p. 162–163 °C, Rf = 0.3 (light petrol-EtOAc 1:1).

^1^H NMR (CDCl_3_, δ, ppm): 1.29 (s, 9H, 3CH_3_, *t*-Bu), 2.07–2.21 (m, 2H, C(6)H_2_), 2.52 (s, 3H, CH_3_, cy-pent-Pyr), 2.55 (s, 3H, CH_3_*,* Pyr), 2.88–2.93 (m, 2H, C(5)H_2_), 2.96–3.01 (m, 2H, C(7)H_2_), 6.60 (s, 1H, CH, Pyr), 7.10–7.23 (m, 4H, 4CH, Ar); 

^13^C NMR (CDCl_3_, δ, ppm): 22.1 (C(6)H_2_), 25.6 (CH_3_, cy-pent-Pyr), 26.1 (CH_3_*,* Pyr), 26.9 (C(5)H_2_), 29.4 (3CH_3_, *t*-Bu), 34.4 (C(7)H_2_), 37.6 (C, *t*-Bu), 99.5 (CH*,* Pyr), 117.3 (C(4a), cy-pent-Pyr), 122.5 (2CH, Ar), 122.7 (2CH, Ar), 149.7 (C, Ar), 149.9 (C, Ar), 165.2 (C(4), cy-pent-Pyr), 166.9 (C(2), cy-pent-Pyr), 167.7 (C(2)*,* Pyr), 170.2 (C(4)*,* Pyr), 177.1 (C(7a), cy-pent-Pyr), 180.4 (C(6), Pyr). 

HRMS (ESI^+^, *m*/*z*): calcd. for C_23_H_26_N_4_O_2_ [M + H]^+^ 391.2129, found 391.2128. 

##### 4-(4-((6-(*tert*-Butyl)-2-methylpyrimidin-4-yl)oxy)phenoxy)-2-methyl-6,7,8,9-tetrahydro-5*H*-cyclohepta[*d*]pyrimidine (**1n**)

Yield 54% (226 mg). White solid, m.p. 149–151 °C, Rf = 0.5 (light petrol-EtOAc 4:1).

^1^H NMR (CDCl_3_, δ, ppm): 1.29 (s, 9H, 3CH_3_, *t*-Bu), 1.61–1.76 (m, 4H, C(6)H_2_, C(8)H_2_), 1.85–1.95 (m, 2H, C(7)H_2_), 2.46 (s, 3H, CH_3_, cy-hept-Pyr), 2.56 (s, 3H, CH_3_*,* Pyr), 2.86–2.93 (m, 2H, C(5)H_2_), 2.93–3.01 (m, 2H, C(9)H_2_), 6.59 (s, 1H, CH, Pyr), 7.15 (br.s, 4H, 4CH, Ar);

^13^C NMR (CDCl_3_, δ, ppm): 24.5 (C(5)H_2_), 25.6 (CH_3_, cy-hept-Pyr), 25.9 (C(8)H_2_), 26.2 (CH_3_, Pyr), 27.1 (C(6)H_2_), 29.4 (3CH_3_, *t*-Bu), 32.5 (C(7)H_2_), 37.6 (C, *t*-Bu), 38.7 (C(9)H_2_), 99.4 (CH*,* Pyr), 118.9 (C(4a), cy-hept-Pyr), 122.3 (2CH, Ar), 122.5 (2CH, Ar), 149.4 (C, Ar), 150.6 (C, Ar), 164.4 (C(2), cy-hept-Pyr), 166.1 (C(4), cy-hept-Pyr), 167.7 (C(2)*,* Pyr), 170.2 (C(4)*,* Pyr), 173.2 (C(9a), cy-hept-Pyr), 180.3 (C(6)*,* Pyr).

HRMS (ESI^+^, *m*/*z*): calcd. for C_25_H_30_N_4_O_2_ [M + H]^+^ 419.2442, found 419.2437. 

##### 2-Methyl-4-(4-((2-methyl-5,6,7,8-tetrahydroquinazolin-4-yl)oxy)phenoxy)-5,6,7,8-tetrahydroquinazoline 1-Oxide (**1o**)

Yield 51% (213 mg). Light-green solid, m.p. 113–116 °C, Rf = 0.3 (light petrol-EtOAc-MeOH 3:1:0.5).

^1^H NMR (CDCl_3_, δ, ppm): 1.72–1.99 (m, 8H, 4CH_2_), 2.46 (s, 3H, CH_3_, THQ), 2.57 (s, 3H, CH_3_, THQ N-O), 2.67–2.86 (m, 6H, 3CH_2_), 2.93–2.98 (m, 2H, CH_2_, THQ N-O), 7.07–7.23 (m, 4H, 4CH); 

^13^C NMR (CDCl_3_, δ, ppm): 19.9 (CH_3_, THQ N-O), 20.8 (C(6)H_2_, THQ N-O), 21.1 (C(7)H_2_, THQ N-O), 21.8 (C(5)H_2_, THQ), 22.1 (C(5)H_2_, THQ N-O), 22.2 (C(6)H_2_, THQ), 22.4 (C(7)H_2_, THQ), 25.2 (C(8)H_2_, THQ N-O), 25.7 (CH_3_, THQ), 32.0 (C(8)H_2_, THQ), 114.1 (C(4a), THQ), 117.6 (C(4a), THQ N-O), 122.2 (2CH), 122.6 (2CH), 149.6 (C, Ar), 150.0 (C, Ar), 154.7 (C(2), THQ N-O), 155.7 (C(4), THQ N-O), 157.5 (C(8a), THQ N-O), 164.2 (C(2), THQ), 166.8 (C(8), THQ), 166.9 (C(4), THQ).

HRMS (ESI^+^, *m*/*z*): calcd. for C_24_H_26_N_4_O_3_ [M + H]^+^ 419.2078, found 419.2069.

##### 4,4′-[1,4-Phenylenebis(oxy)]bis(2-methyl-5,6,7,8-tetrahydroquinazoline) 1,1′-Dioxide (**1p**) [30]

Yield 30% (130 mg). Yellowish solid, m.p. 240–242 °C with decomposition, Rf = 0.7 (DCM-MeOH 20:1).

^1^H NMR (CDCl_3_, δ, ppm): 1.77–1.87 (m, 4H, 2CH_2_), 1.88–1.98 (m, 4H, 2CH_2_), 2.57 (s, 6H, 2CH_3_), 2.75–2.60 (m, 4H, 2CH_2_), 2.95–3.00 (m, 4H, 2CH_2_), 7.16 (s, 4H, 4CH).

### 3.2. Electrophysiological Evaluation

In vitro electrophysiological experiments were carried out using a patch clamp technique with the local fixation of potential, as described earlier [28,29,37]. Freshly isolated single Purkinje neurons from the cerebellum of 12–15-day-old Wistar rats were used as a test system. Transmembrane currents were induced by the activation of the AMPA receptors with a solution of their partial agonist kainic acid, using a fast superfusion of solutions, wherein 30 μL of the agonist buffer (the agonist concentration varied in the range of 10^−6^–10^−4^ M) was added to the constant flow of the neuron-washing buffer. The applications for the control and for each concentration of a compound were performed in triplicate. The transmembrane currents for the individual neurons were recorded using 2.5–5.5 MΩ borosilicate microelectrodes in a whole-cell configuration with an EPC-9 device from HEKA, Germany. The data were processed using the Pulsfit program from HEKA, Germany. Cyclothiazide (CTZ), as a well-known positive allosteric modulator of AMPA receptors, was used as a reference ligand. The experimental results for compounds **1a**–**p** are presented in Table 1.

### 3.3. Molecular Modeling

The structure of the dimeric ligand binding domain of the GluA2 AMPA receptor was obtained from the Protein Data Bank (PDB: 4FAT) [49]. Upon the removal of the ions and small molecules (except for the two receptor-bound glutamate agonist molecules), the protein was allowed to relax during the molecular dynamics simulation for 100 ns (see below for the simulation protocol). The most frequently occurring structure was identified by the clustering of the frames in the stable part of the trajectory (40–100 ns). The ligand structure was converted to 3D and preoptimized in the MMFF94 force field using Avogadro 1.2.0 software (Avogadro Chemistry, https://avogadro.cc/, accessed on 1 October 2022) [50], and then the ligand and protein structures were prepared for molecular docking using AutoDock Tools 1.5.7 (The Scripps Research Institute, La Jolla, CA, USA, https://ccsb.scripps.edu/mgltools/, accessed on 1 October 2022) [51]. The molecular docking to the positive allosteric modulator binding site was performed with AutoDock Vina 1.1.2 software (The Scripps Research Institute, La Jolla, California, USA, https://vina.scripps.edu/, accessed on 1 October 2022) [52] (grid box size 22 Å × 29 Å × 40 Å, exhaustiveness = 16). The pose with the best scoring function value and ligand position was selected, and the complex model was built using the USCF Chimera 1.15 software (University of California San Francisco, San Francisco, USA, https://www.cgl.ucsf.edu/chimera/, accessed on 1 October 2022) [53].

The molecular dynamics simulations were performed using the CHARMM36/CGenFF 4.6 force field [54,55] on the GROMACS 2021.2 software (GROMACS development team, https://www.gromacs.org/, accessed on 1 October 2022) [56]. The initial models of the systems were built using the Ligand Reader & Modeler and Solution Builder modules of the CHARMM-GUI web service [57,58]. The protein molecule was inserted into a rectangular box of water in the TIP3P model; the distance from the protein to the box border was no less than 10 Å. Individual, randomly selected water molecules were replaced with potassium and chlorine ions to ensure the electrical neutrality of the system and the total concentration of KCl of approximately 0.15 M. For each system, the molecular mechanics minimization (up to 5000 steps) was performed on the CPU, followed by equilibration for 125 ps at the temperature of 300 K and a constant volume using the v-rescale thermostat on the NVIDIA GeForce RTX 3080 GPU. The production simulation was performed on the GPU at the constant pressure of 1 bar and the temperature of 300 K, using the v-rescale thermostat and the Parrinello–Rahman barostat. The hydrogen atom movements were constrained using the LINCS algorithm. For the analysis and visualization of the results, the CPPTRAJ software (Daniel R. Roe, Amber development team, http://ambermd.org/, accessed on 1 October 2022) [59] in the AmberTools 22 package [60] and UCSF Chimera were used. The binding free energies were estimated over the stable portion of the trajectories (last 20 ns, 101 frames at 200 ps interval) using the MM/GBSA approach implemented using the gmx_MMPBSA 1.5.5 software (gmx_MMPBSA development team, https://valdes-tresanco-ms.github.io/gmx_MMPBSA/dev/, accessed on 1 October 2022) [61,62]. The internal dielectric constant ε = 4, a salt concentration of 0.15 M, and the interaction entropy model for the conformation entropy contribution were used. The resulting energy values are listed in Table 3.

### 3.4. Prediction of ADMET, Physicochemical, and PAINS Profiles

The lipophilicity (LogP_ow_) and aqueous solubility (pS_aq_) were estimated using the ALogPS 3.0 neural network model implemented on the OCHEM platform [63]. Human intestinal absorption (HIA) [64], blood–brain barrier permeability (LogBB) [65,66], and hERG-mediated cardiac toxicity risk (channel affinity p*K_i_* and inhibitory activity pIC_50_) [67] were estimated using the integrated online service for ADMET properties prediction (ADMET Prediction Service) [68]. This server implements predictive QSAR models based on accurate and representative training sets, fragmental descriptors, and artificial neural networks. The quantitative estimate of drug-likeness (QED) values [69] were calculated and the pan-assay interference compounds (PAINS) alerts were checked using RDKit version 2020.03.4 software [70].

## 4. Conclusions

To summarize, a novel series of subnanomolar positive allosteric modulators of the AMPA receptor was developed. For this purpose, a convenient, flexible, simple, and efficient preparative approach to the previously unknown substituted bis(pyrimidines) with a hydroquinone linker was elaborated. Due to the employment of the benzyl protective group, this three-step approach allows one to obtain both symmetric and non-symmetric bis(pyrimidines), and can be successfully used for targeted synthesis or the creation of combinatorial libraries. Many compounds of this series demonstrated remarkable activity as positive or negative allosteric modulators of the AMPA receptor. The positive allosteric modulators such as **1f**, **1h**, **1i**, **1k**, **1o** caused a potentiation of the kainate-induced AMPA receptor currents of up to 77% in a wide concentration range (10^−12^–10^−6^ M). The molecular modeling confirmed that these compounds could interact with the validated PAM binding site. Their predicted ADMET, physicochemical, and PAINS properties were quite acceptable for potential lead compounds at the early drug development stages. We expect that more detailed analysis of their binding and interactions, coupled with further exploration of this scaffold and subsequent in vitro and in vivo investigations will allow one to develop more potent and safer positive and negative AMPA receptor modulators with a wide range of potential psychopharmacological applications, including nootropic, neuroprotective, and antiepileptic agents.

## Figures and Tables

**Table 2:** Predicted physicochemical and ADMET profiles of compounds **1a**–**p**.

Compound	MW	LogP_ow_	pS_aq_	LogBB	HIA	hERG p*K_i_*	hERG pIC_50_	QED
**1a**	402.50	5.11	6.63	−0.34	84	5.37	4.33	0.60
**1b**	430.55	5.75	6.91	−1.43	84	5.64	4.36	0.50
**1c**	458.61	5.93	7.13	−0.29	93	5.37	4.63	0.40
**1d**	486.66	6.09	7.89	−0.27	100	6.35	4.41	0.39
**1e**	454.57	5.48	7.08	−0.23	93	5.78	4.56	0.43
**1f**	374.44	4.36	5.49	−1.60	84	5.37	4.59	0.66
**1g**	374.44	4.36	5.77	−0.40	84	5.13	4.57	0.67
**1h**	430.55	5.64	7.23	−0.28	84	5.39	4.64	0.47
**1i**	406.53	5.01	6.59	0.22	100	7.37	4.69	0.52
**1j**	388.47	4.75	6.21	−0.37	84	5.25	4.45	0.64
**1k**	416.53	5.38	6.92	−0.31	84	5.38	4.48	0.51
**1l**	404.51	5.15	6.60	−0.05	97	6.39	4.51	0.56
**1m**	390.49	4.80	6.24	−0.08	97	6.26	4.63	0.61
**1n**	418.54	5.42	6.88	−0.02	97	6.39	4.66	0.48
**1o**	418.50	3.52	4.03	−0.44	84	5.23	4.40	0.46
**1p**	434.50	2.37	2.96	−0.53	84	5.09	4.48	0.46

Note: MW—molecular weight, LogP_ow_—octanol-water partition coefficient, pS_aq_—aqueous solubility [−log(M)], LogBB—blood–brain barrier permeability, HIA—human intestinal absorption [%], hERG *pK_i_*—hERG potassium-channel affinity [−log(M)], hERG pIC_50_—hERG potassium channel inhibitory activity [−log(M)], QED—quantitative estimate of drug-likeness.

**Table 3:** Binding free energy for compounds **1f**, **1i**, **1j**, calculated using MM/GBSA approach.

Energy Terms, kcal/mol	Compound 1f	Compound 1i	Compound 1j
Δ*E_int_*	0 ± 0	0 ± 0	0 ± 0
Δ*E_ele_*	−0.76 ± 0.08	−2.4 ± 0.1	−2.0 ± 0.1
Δ*E_vdw_*	−43.2 ± 0.3	−29.65 ± 0.3	−37.2 ± 0.2
Δ*E_MM_* = Δ*E_int_* + Δ*E_ele_* + Δ*E_vdw_*	−44.0 ± 0.3	−32.1 ± 0.4	−39.2 ± 0.2
Δ*G_GB_*	5.6 ± 0.1	7.2 ± 0.1	8.8 ± 0.1
Δ*G_SA_*	−5.79 ± 0.02	−4.51 ± 0.05	−4.71 ± 0.02
Δ*G_sol_* = Δ*G_GB_* + Δ*G_SA_*	−0.19 ± 0.06	2.6 ± 0.1	4.1 ± 0.1
Δ*G_MMGBSA_* = Δ*E_MM_* + Δ*G_sol_*	−44.2 ± 0.3	−29.4 ± 0.4	−35.1 ± 0.2
−*T*Δ*S*	2.6 ± 0.1	3.8 ± 0.2	2.2 ± 0.2
Δ*G_b_* = Δ*G_MMGBSA_* − *T*Δ*S*	−41.6 ± 0.3	−26.2 ± 0.4	−30.9 ± 0.3

Note: Values are listed as Mean ± Standard Error of Mean.

## Data Availability

Not applicable.

## Supplementary Materials

The following supporting information can be downloaded at: https://www.mdpi.com/article/10.3390/molecules27238252/s1, details of optimization of the reaction conditions; copies of NMR spectra, copies of HRMS spectra, kainate-induced AMPA receptor currents plot (Figure S1), parameters of molecular dynamics simulation of the modulator-receptor complexes (Figures S2–S4).

## Sample Availability

Samples of the compounds **1a**–**p** are available from the authors.
