# Supplementary material for: Novel Nanomolar Allosteric Modulators of AMPA Receptor of Bis(pyrimidine) Series: Synthesis, Biotesting and SAR Analysis
Source: Molecules. 2022 Nov 26;27(23):8252. doi: 10.3390/molecules27238252 (PMC9741252; doi:10.3390/molecules27238252)
Supplement: Supplementary file 1 [file molecules-27-08252-s001.zip › molecules-2038912-supplementary.pdf]

Novel Nanomolar Allosteric Modulators of AMPA Receptor of Bis(pyrimidine) Series: Synthesis, Biotesting and SAR Analysis

Kseniya N. Sedenkova, Denis V. Zverev, Anna A. Nazarova, Mstislav I. Lavrov, Eugene V. Radchenko, Yuri K. Grishin, Alexey V. Gabrel'yan, Vladimir L. Zamoyski, Vladimir V. Grigoriev, Elena B. Averina and Vladimir A. Palyulin*

Supplementary information

1. **Optimization of reactions conditions** **2**
2. **Copies of NMR spectra** **3**
3. **Copies of HRMS spectra** **76**
4. **Kainate-induced AMPA receptor currents** **86**
5. **Parameters of molecular dynamics simulation of the modulator-receptor complexes** **87**

1. Optimization of reaction conditions

Table S1. Optimization of the conditions of S_NAr reaction of compounds **2a** and **3**

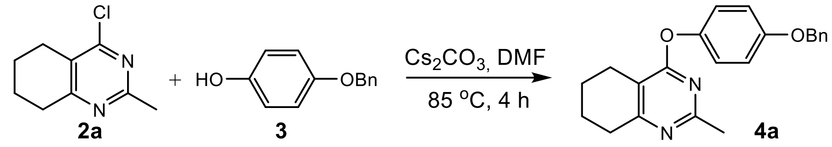

(**2a** : **3** : Cs_2CO_3) ratio	(**2a** : **4a**) ratio in the reaction mixture according to ^{1}H NMR spectra
1 : 1 : 1	1 : 1
1 : 1.5 : 1.5	0.3 : 1
1 : 2 : 2	0 : 1

Table S2. Optimization of the conditions of S_NAr reaction of compound **2a** and **5a**

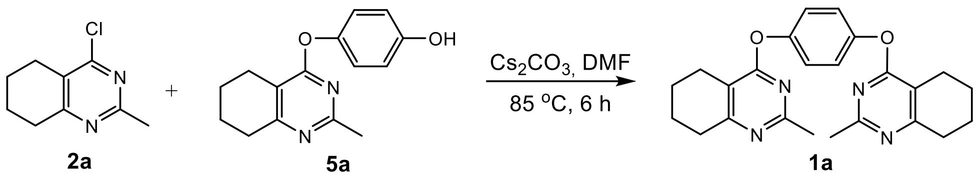

(**2a** : **5a** : Cs_2CO_3) ratio	(**5a** : **1a**) ratio in the reaction mixture according to ^{1}H NMR spectra
1.5 : 1 : 1	1 : 1
2 : 1 : 2	0 : 1

2. Copies of NMR Spectra

^{1}H NMR spectrum ($CDCl_3$) of compound **2c**

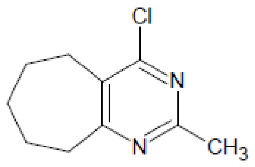

2c

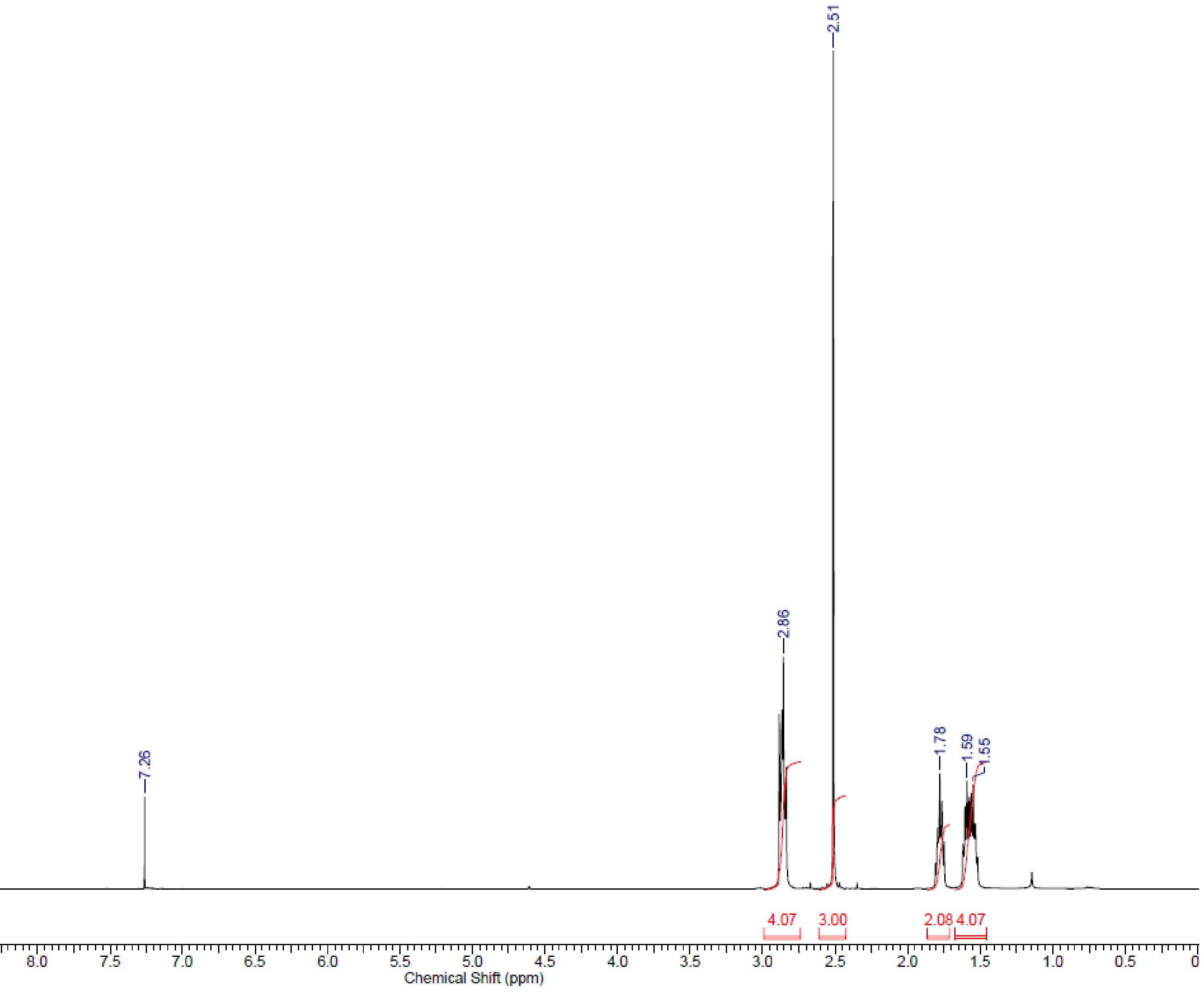

^{13}C NMR spectrum ($CDCl_3$) of compound **2c**

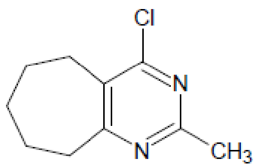

2c

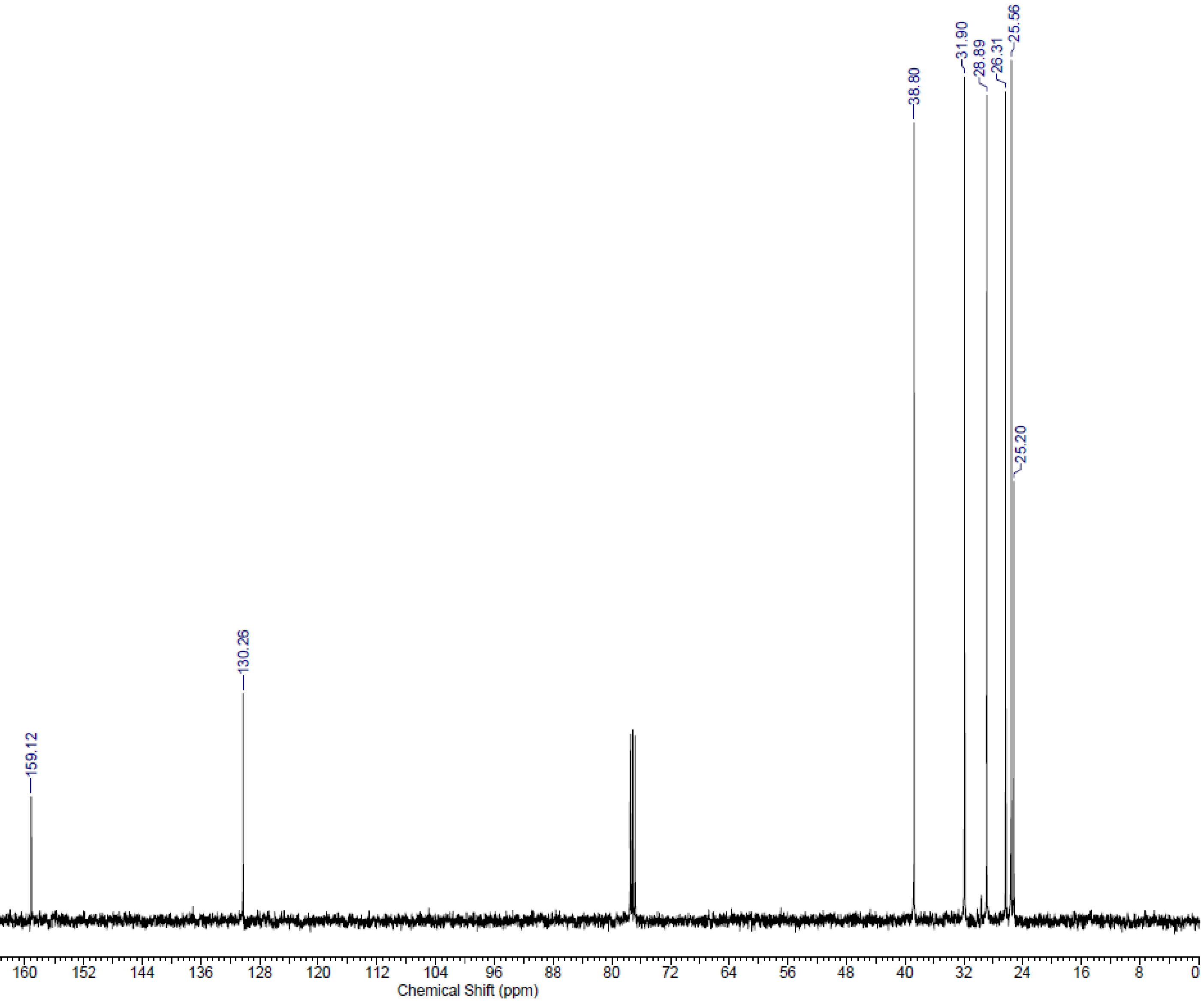

HSQC NMR spectrum ($CDCl_3$) of compound **2c**

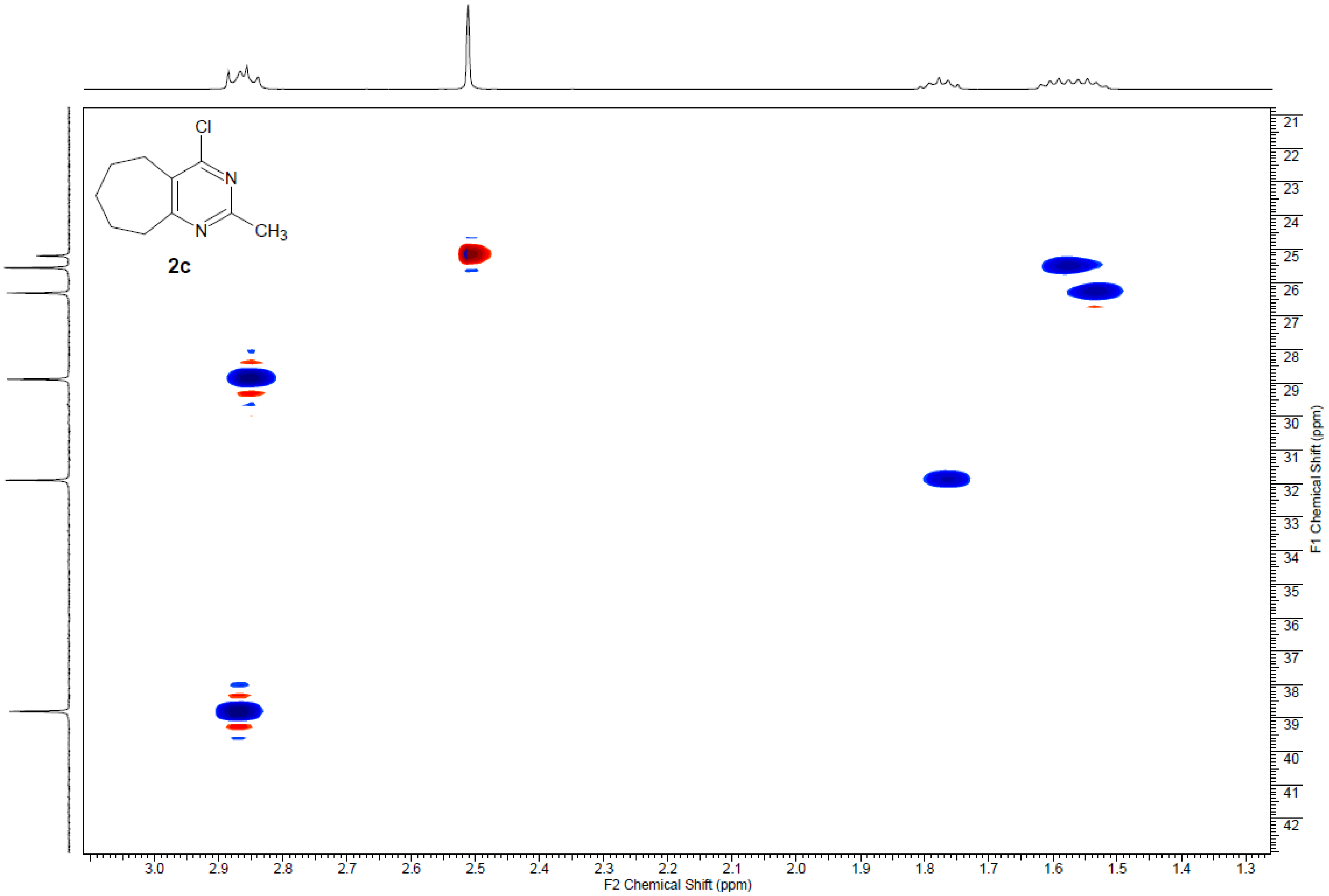

HMBC NMR spectrum ($CDCl_3$) of compound **2c**

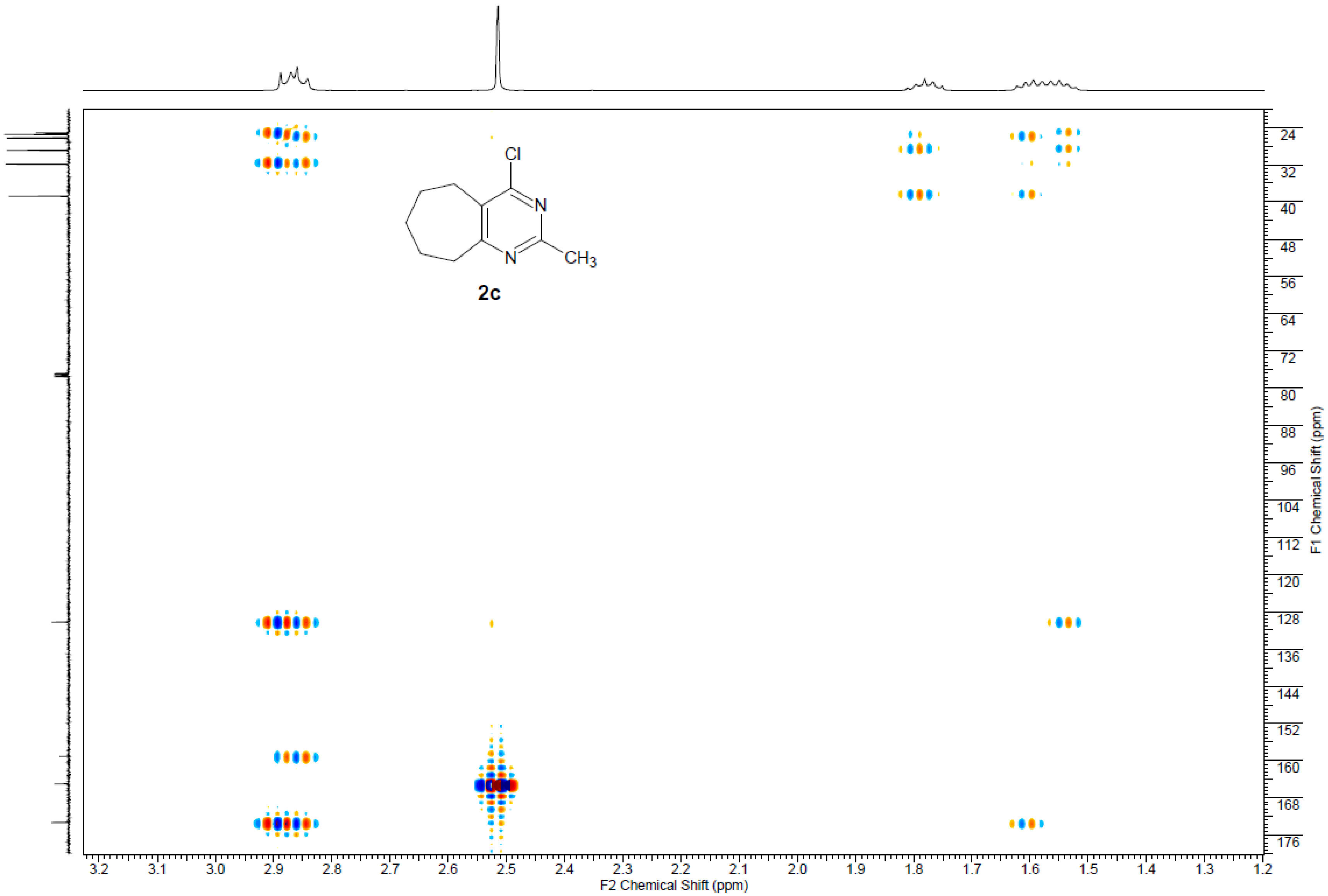

^{1}H NMR spectrum ($CDCl_3$) of compound **4a**

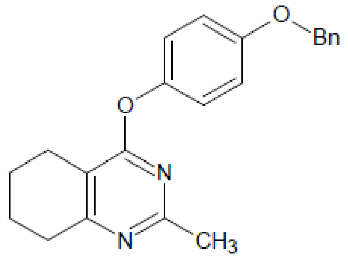

4a

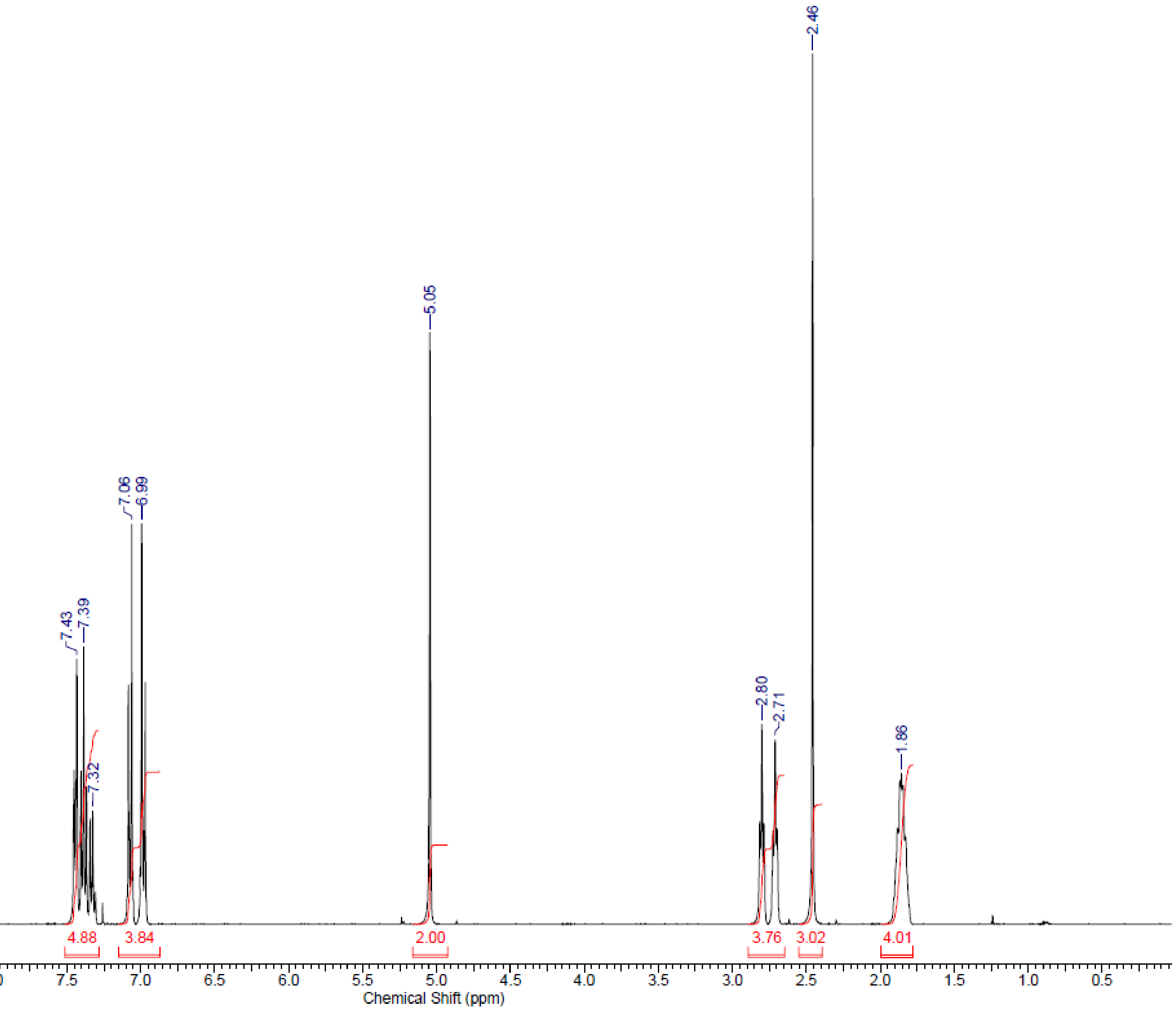

^{13}C NMR spectrum ($CDCl_3$) of compound **4a**

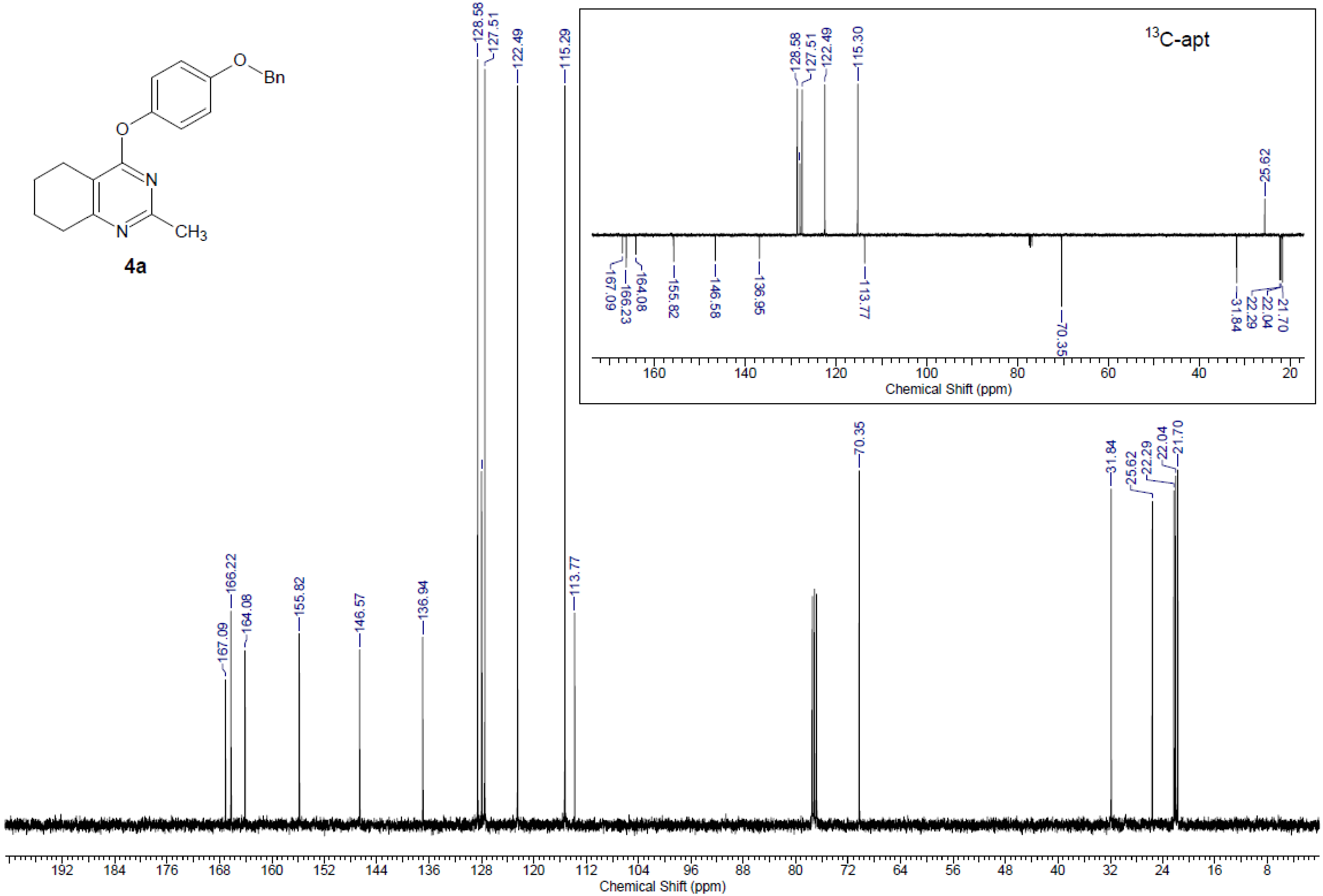

^{1}H NMR spectrum ($CDCl_3$) of compound **4b**

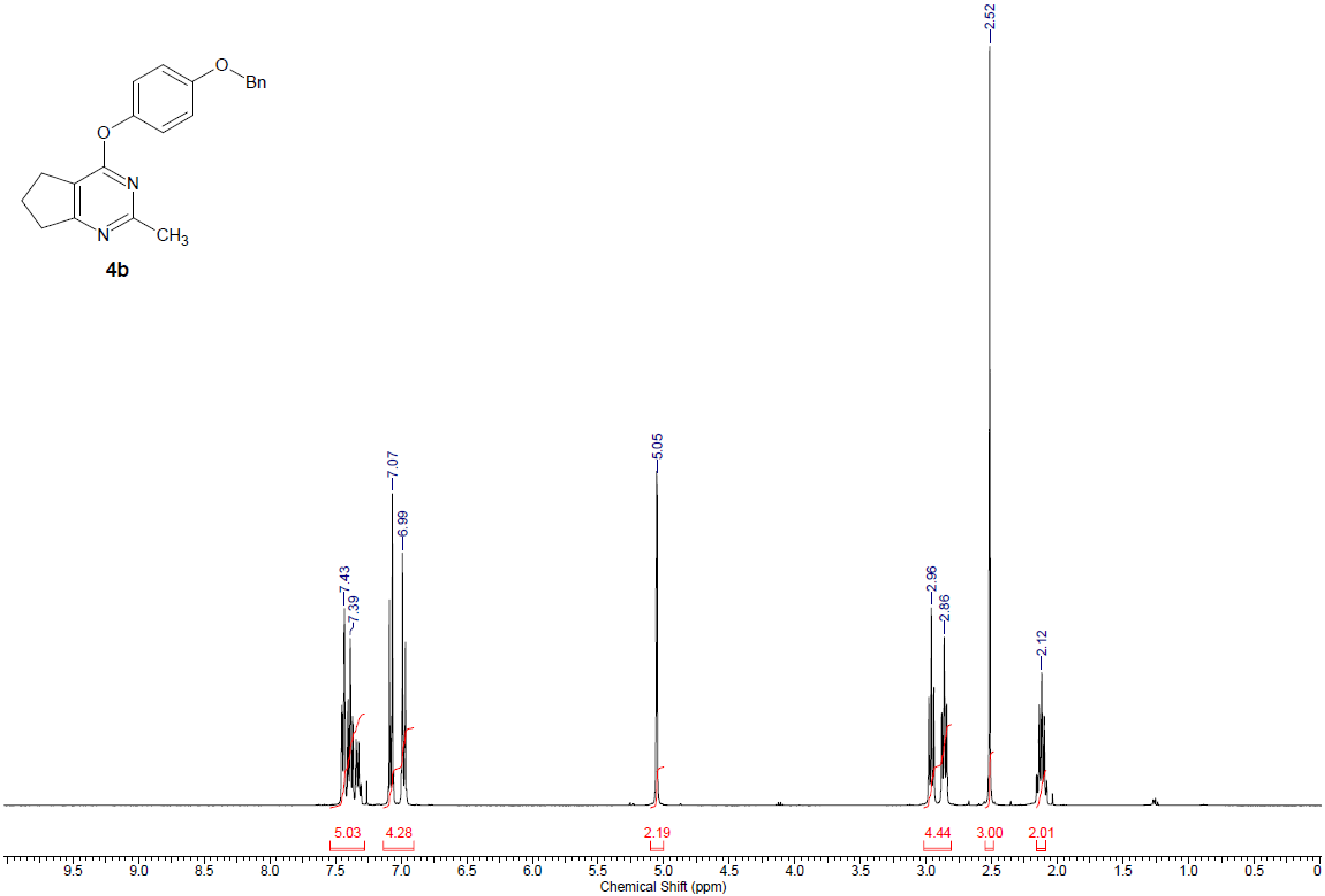

^{13}C NMR spectrum ($CDCl_3$) of compound **4b**

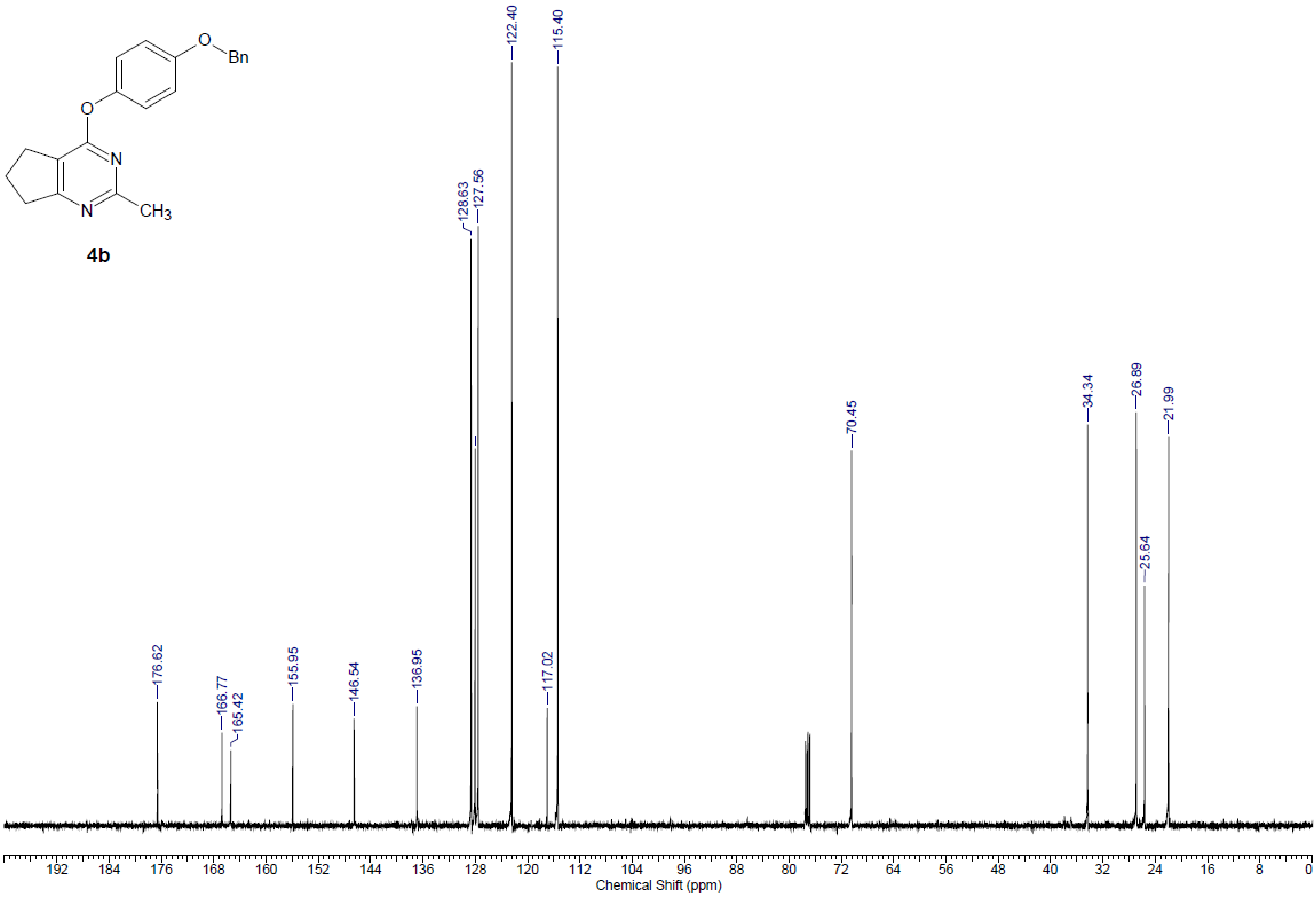

HSQC NMR spectrum ($CDCl_3$) of compound **4b**

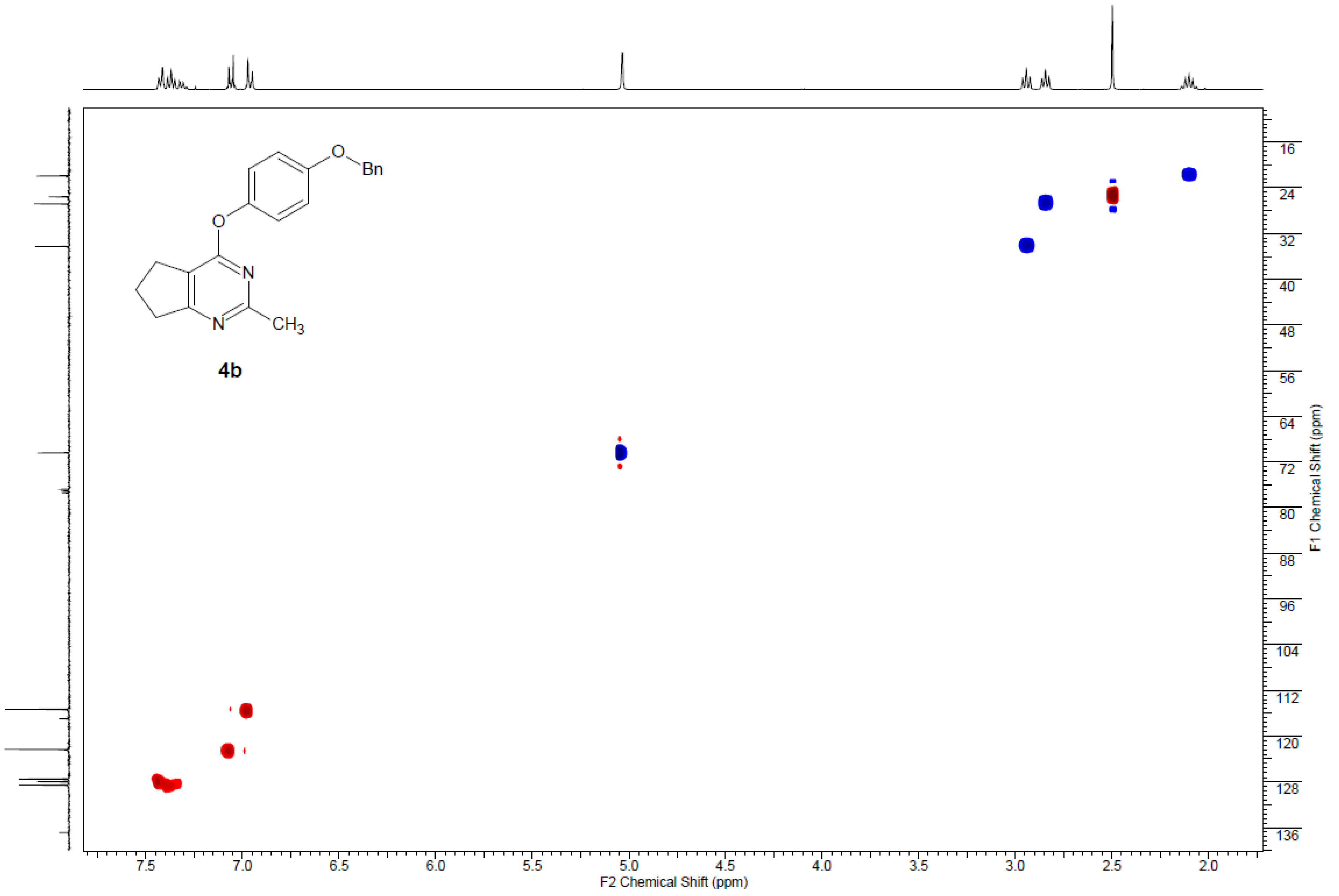

HMBC NMR spectrum ($CDCl_3$) of compound **4b**

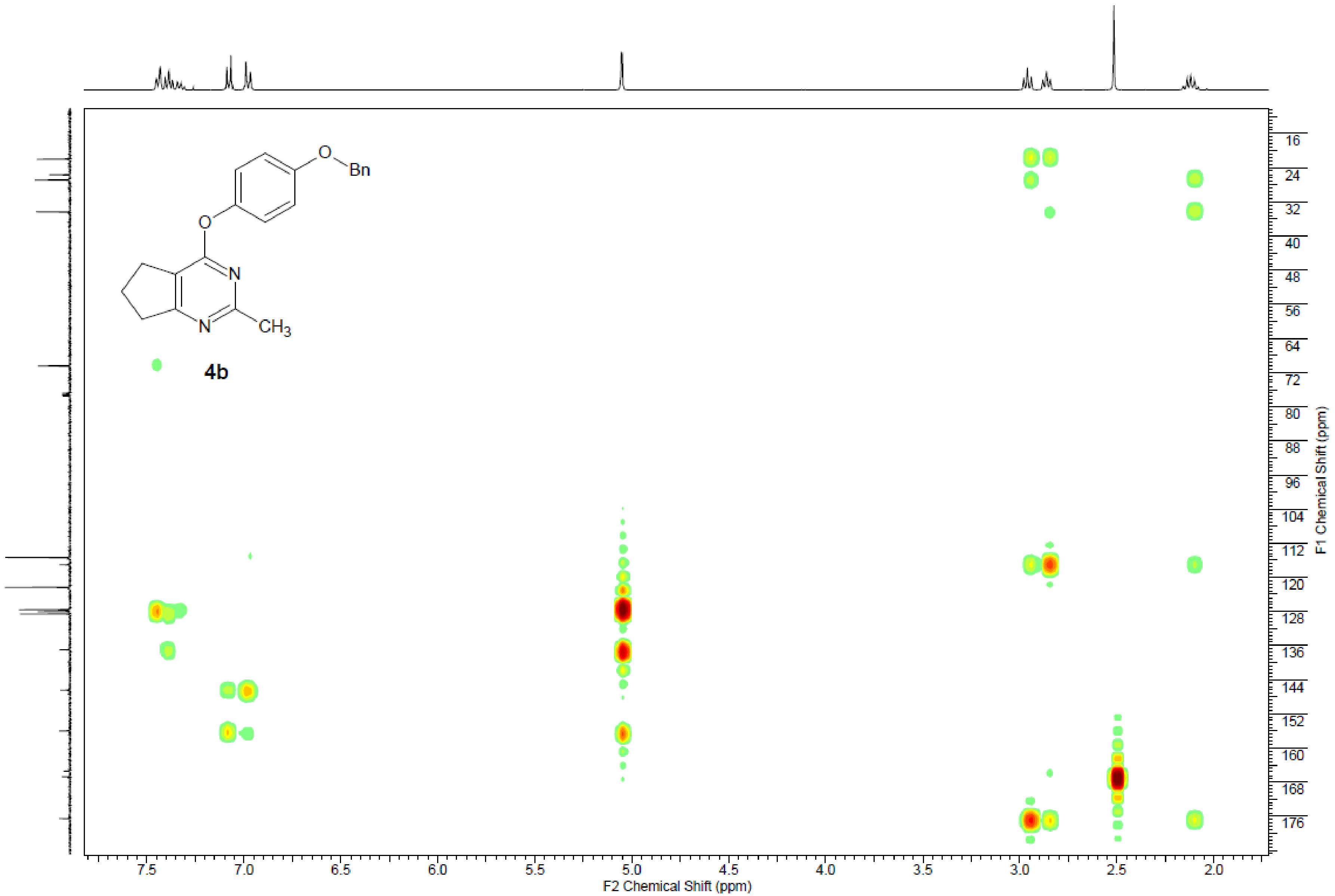

^{1}H NMR spectrum ($CDCl_3$) of compound **4c**

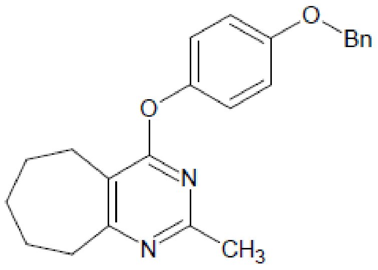

4c

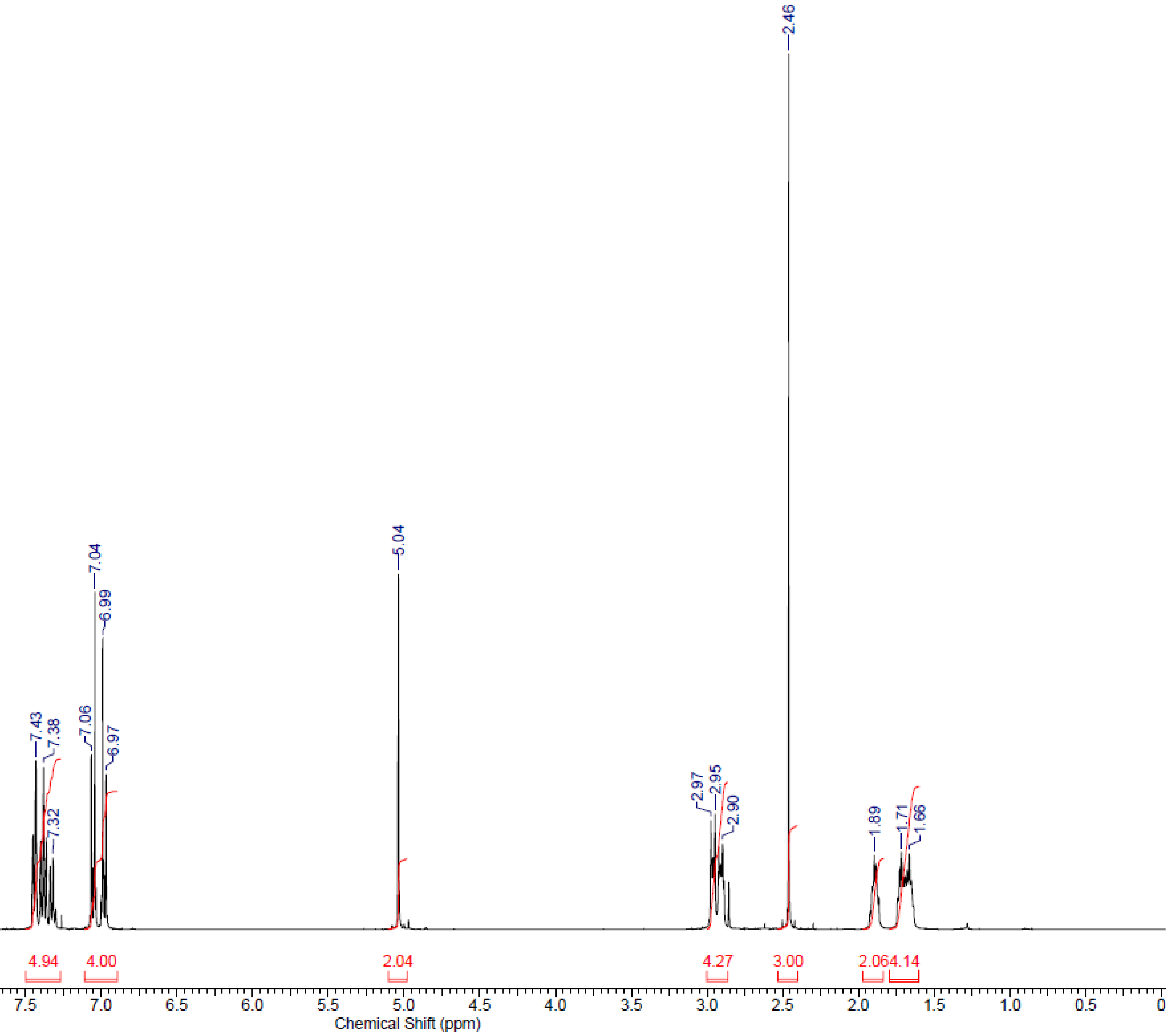

^{13}C NMR spectrum ($CDCl_3$) of compound **4c**

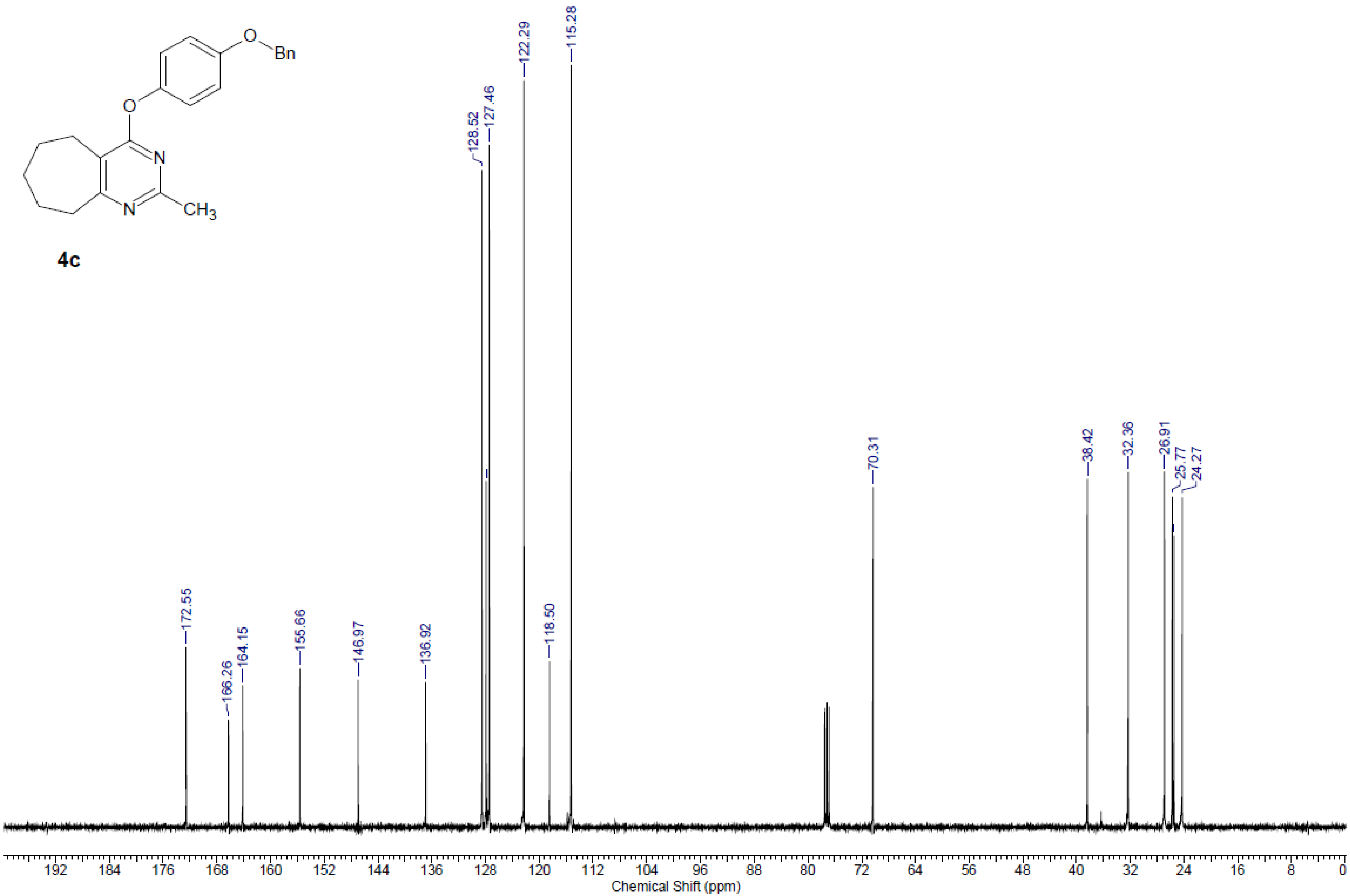

4c

HSQC NMR spectrum ($CDCl_3$) of compound **4c**

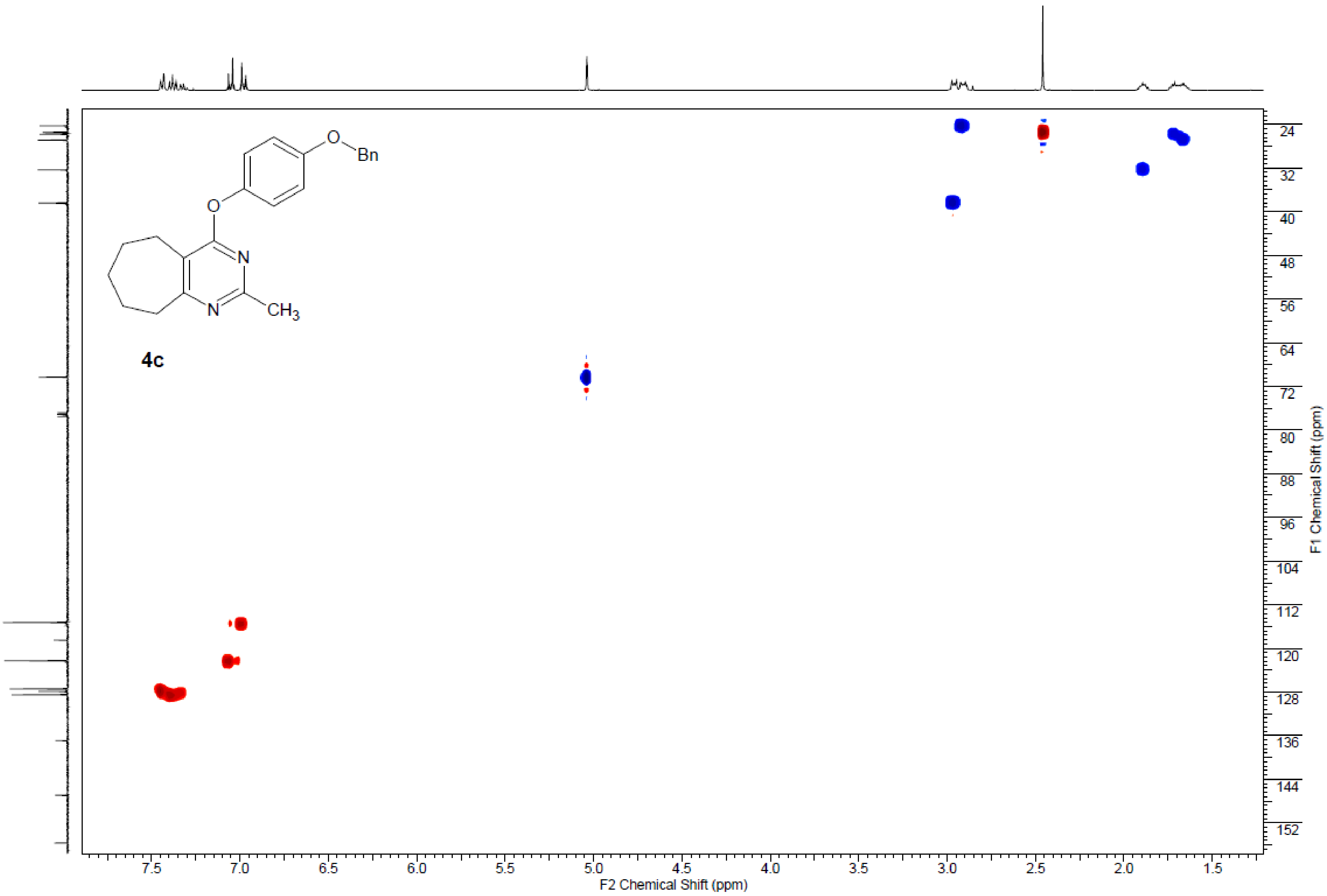

HMBC NMR spectrum ($CDCl_3$) of compound **4c**

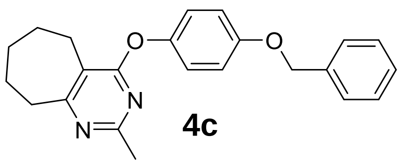

4c

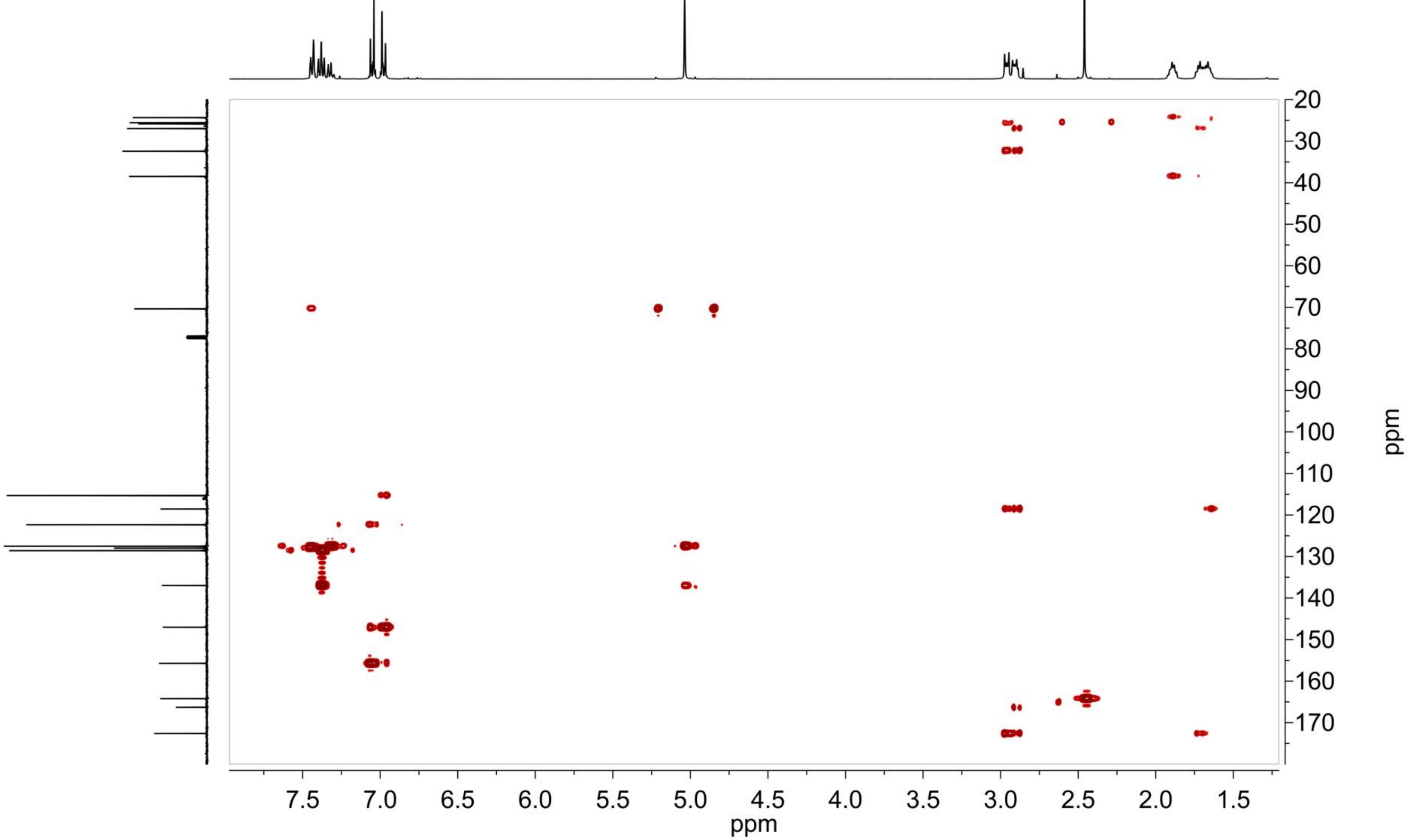

^{1}H NMR spectrum ($CDCl_3$) of compound **4d**

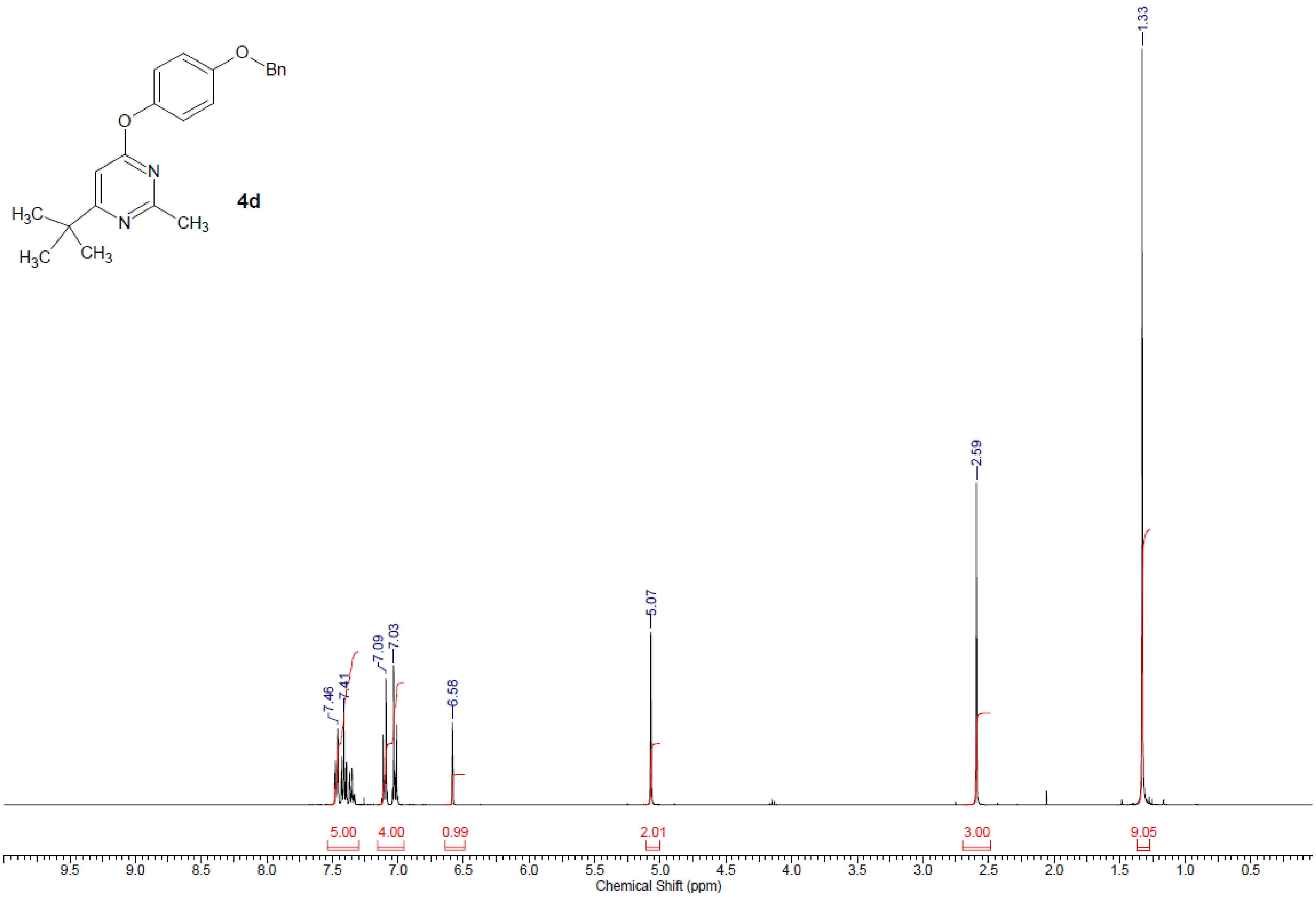

^{13}C NMR spectrum ($CDCl_3$) of compound **4d**

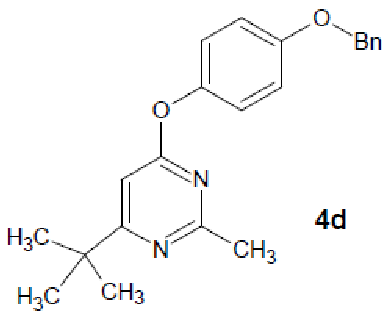

4d

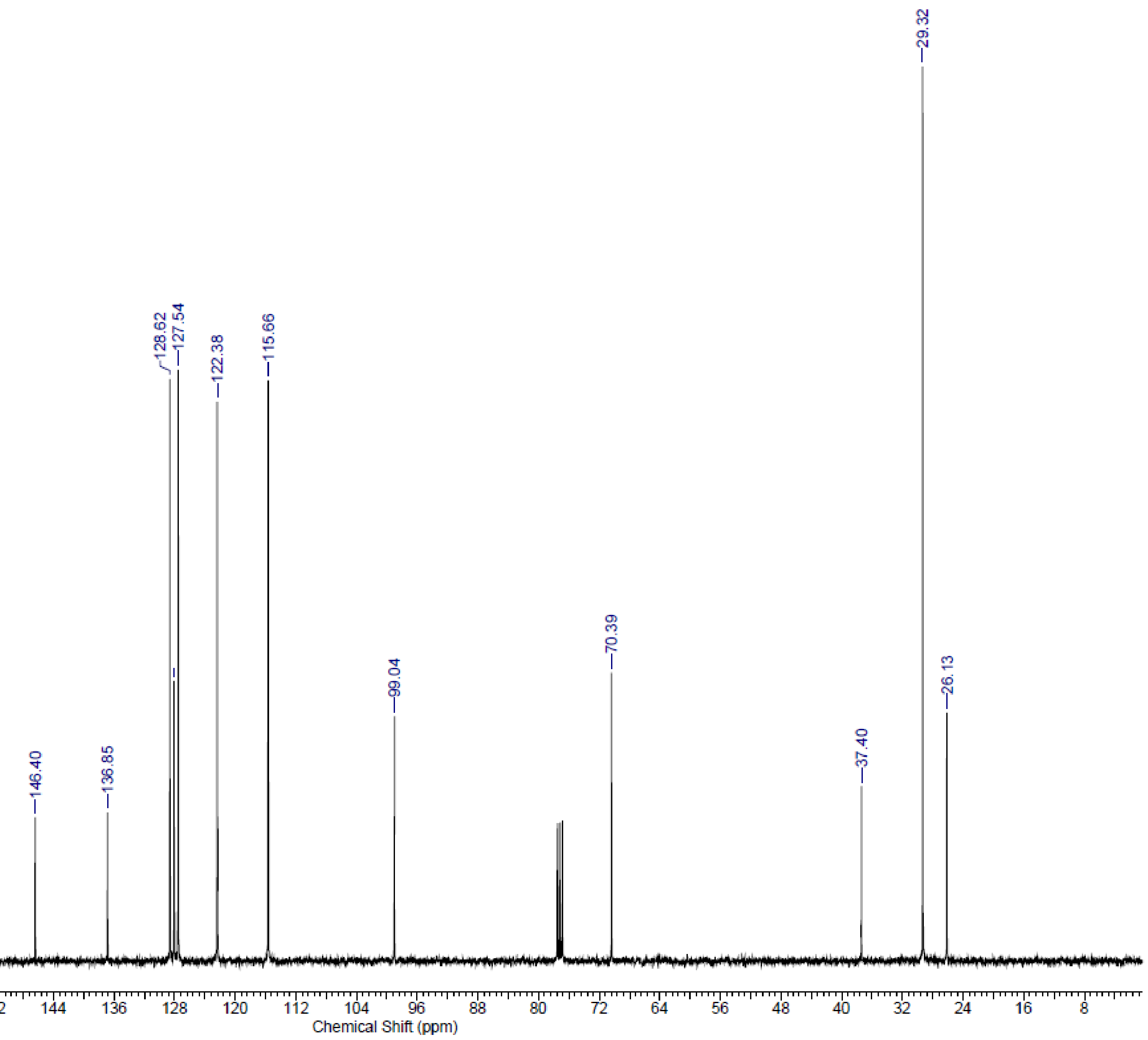

^1H NMR spectrum ($CDCl_3$) of compound **4e**

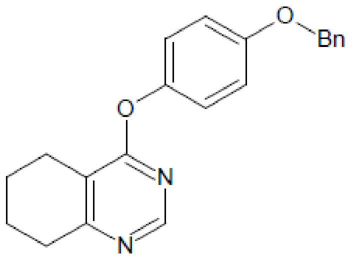

4e

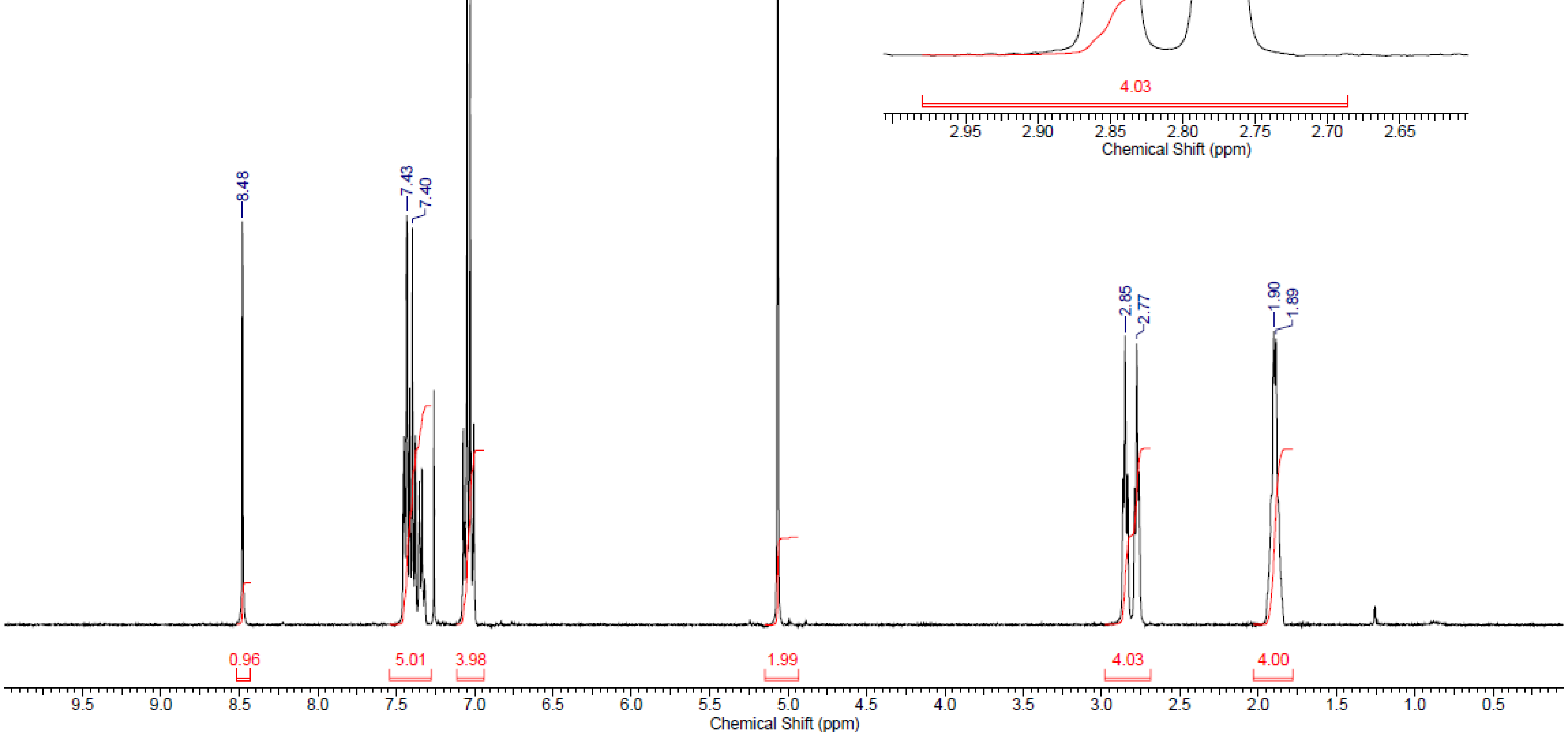

^{13}C NMR spectrum ($CDCl_3$) of compound **4e**

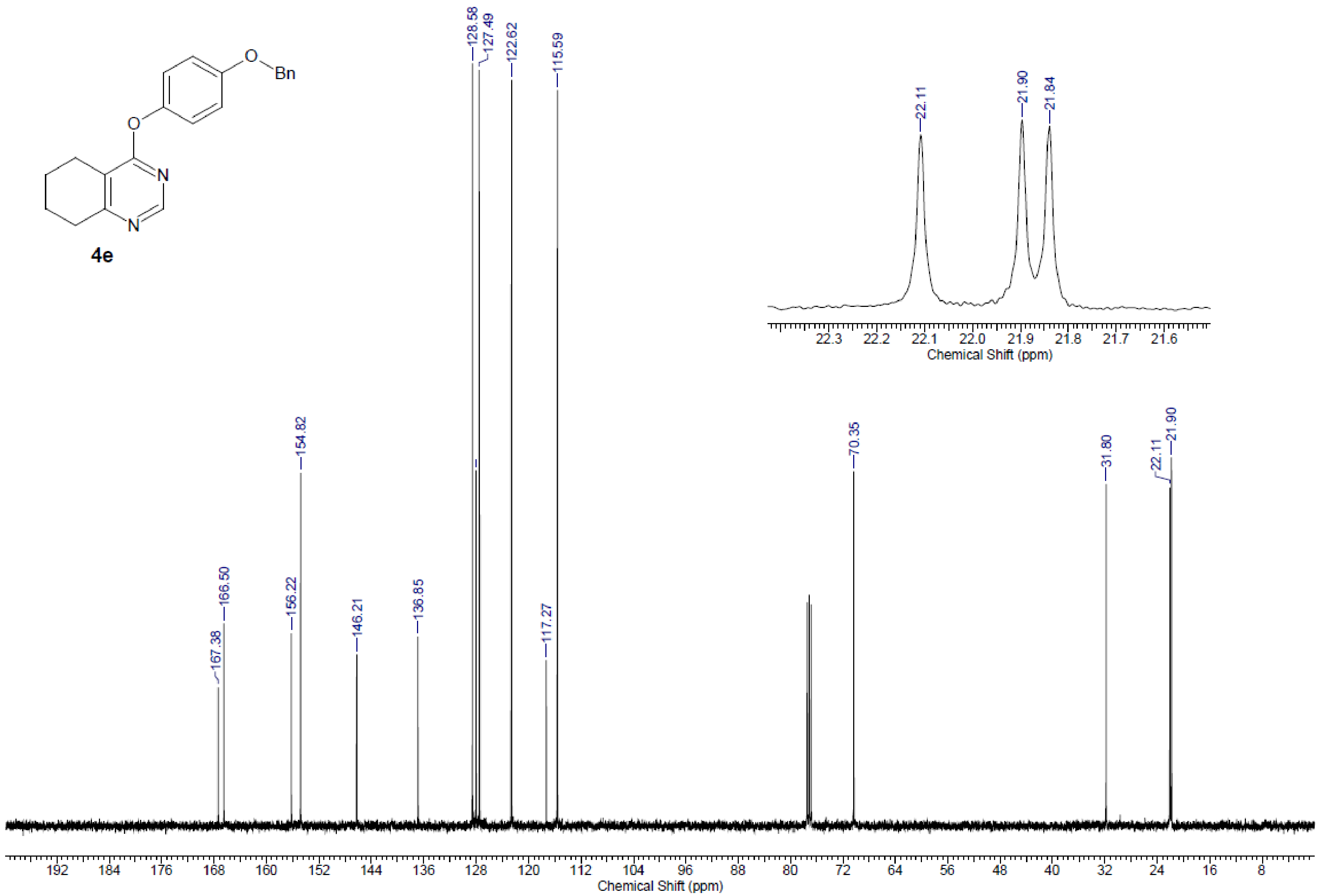

^{1}H NMR spectrum ($CDCl_3$) of compound **7**

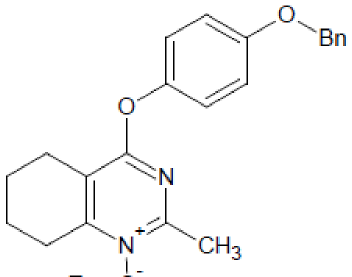

7

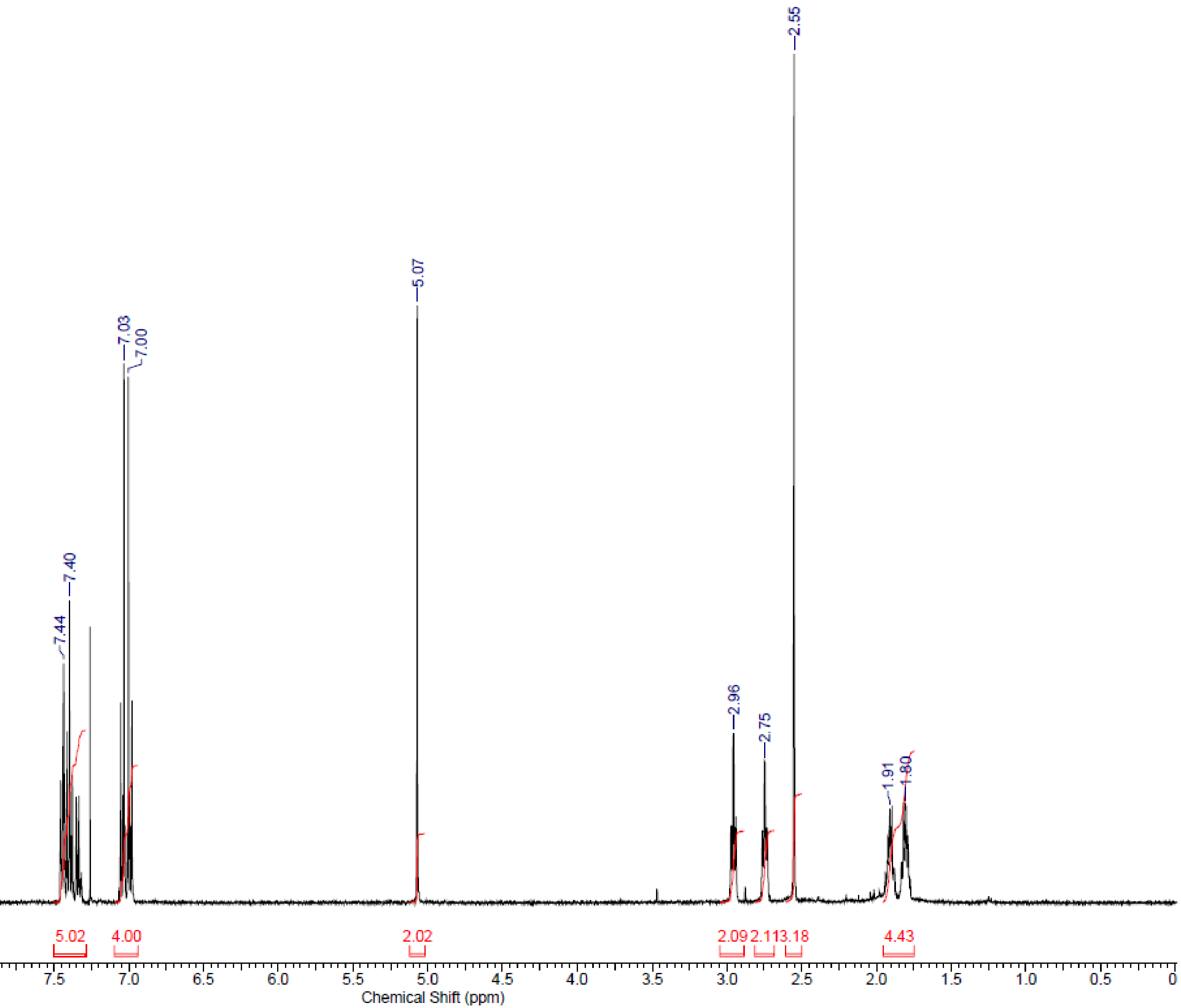

^{13}C NMR spectrum ($CDCl_3$) of compound **7**

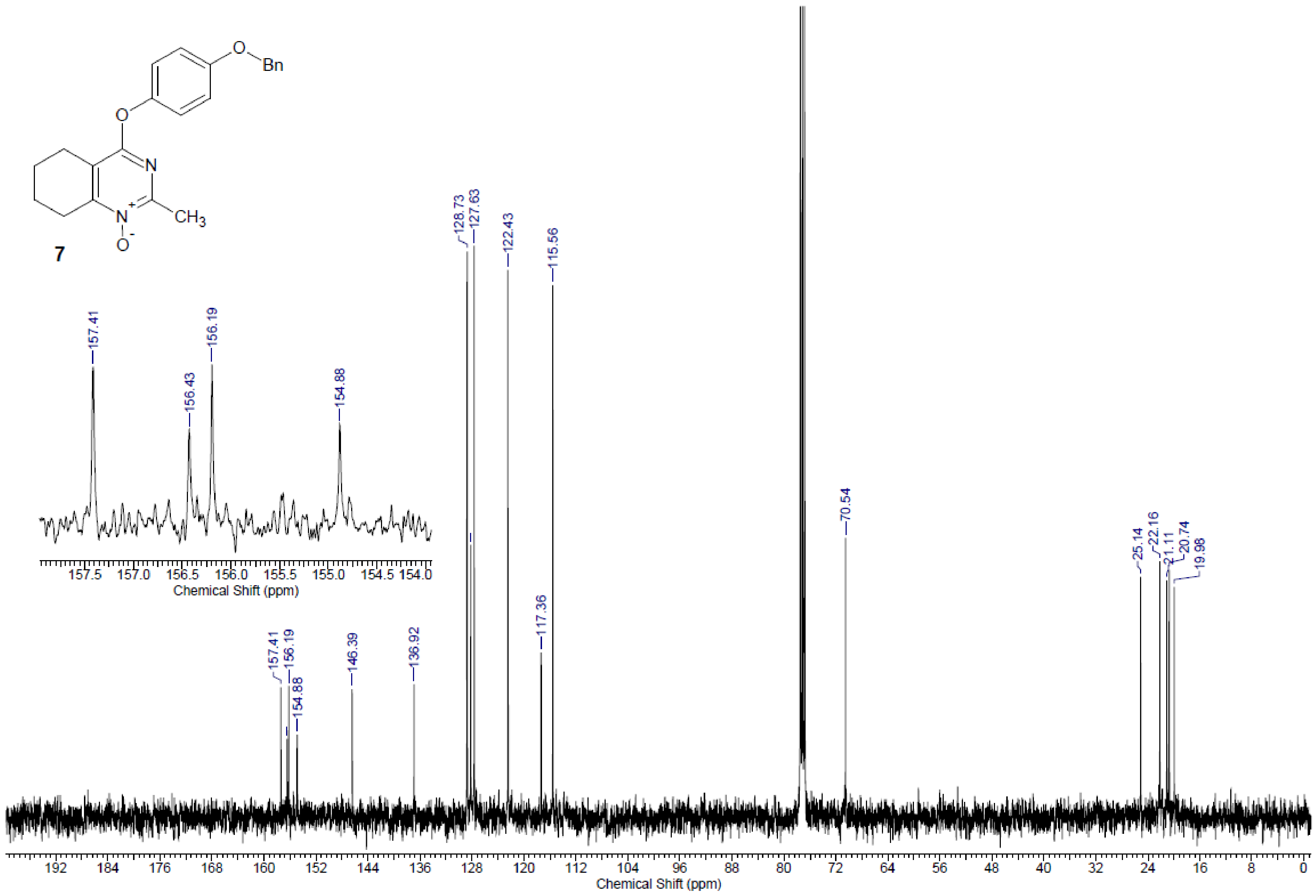

^{1}H NMR spectrum ($CDCl_3$) of compound **5a**

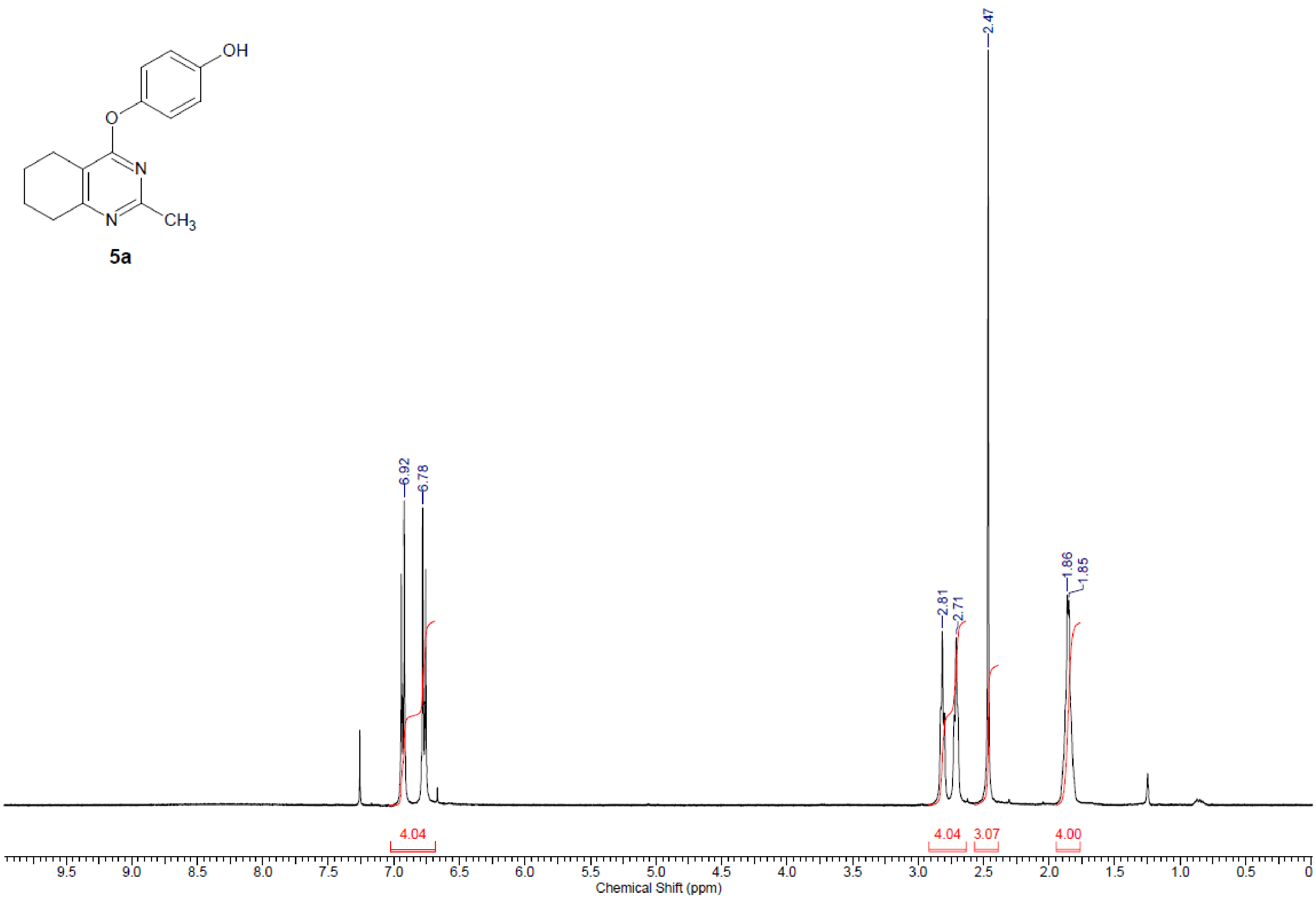

^{13}C NMR spectrum ($CDCl_3$) of compound **5a**

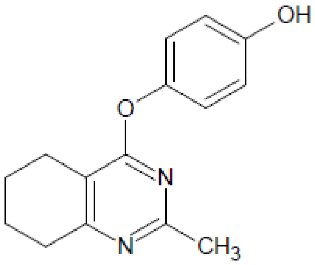

5a

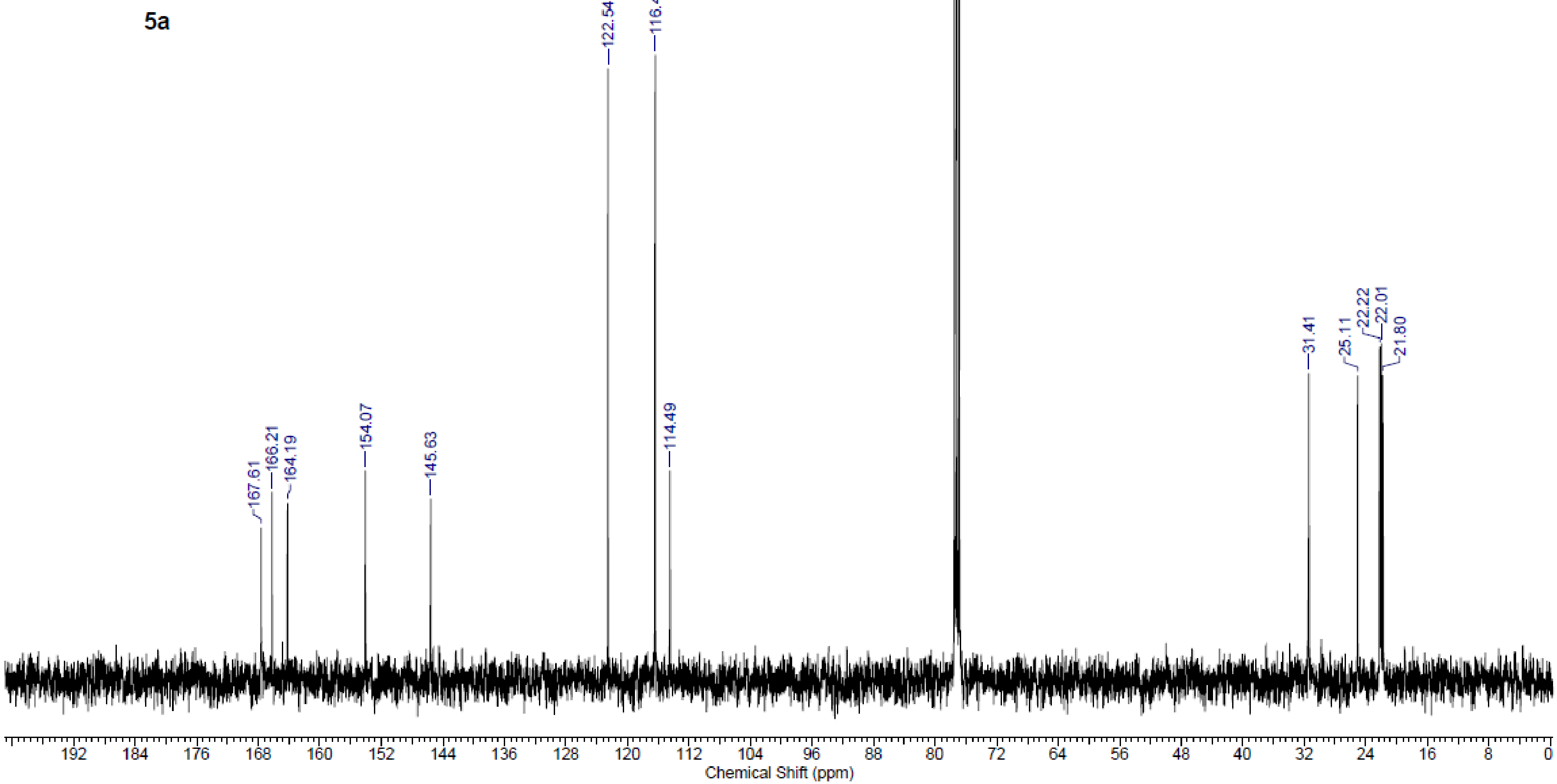

^{1}H NMR spectrum ($CDCl_3$) of compound **5b**

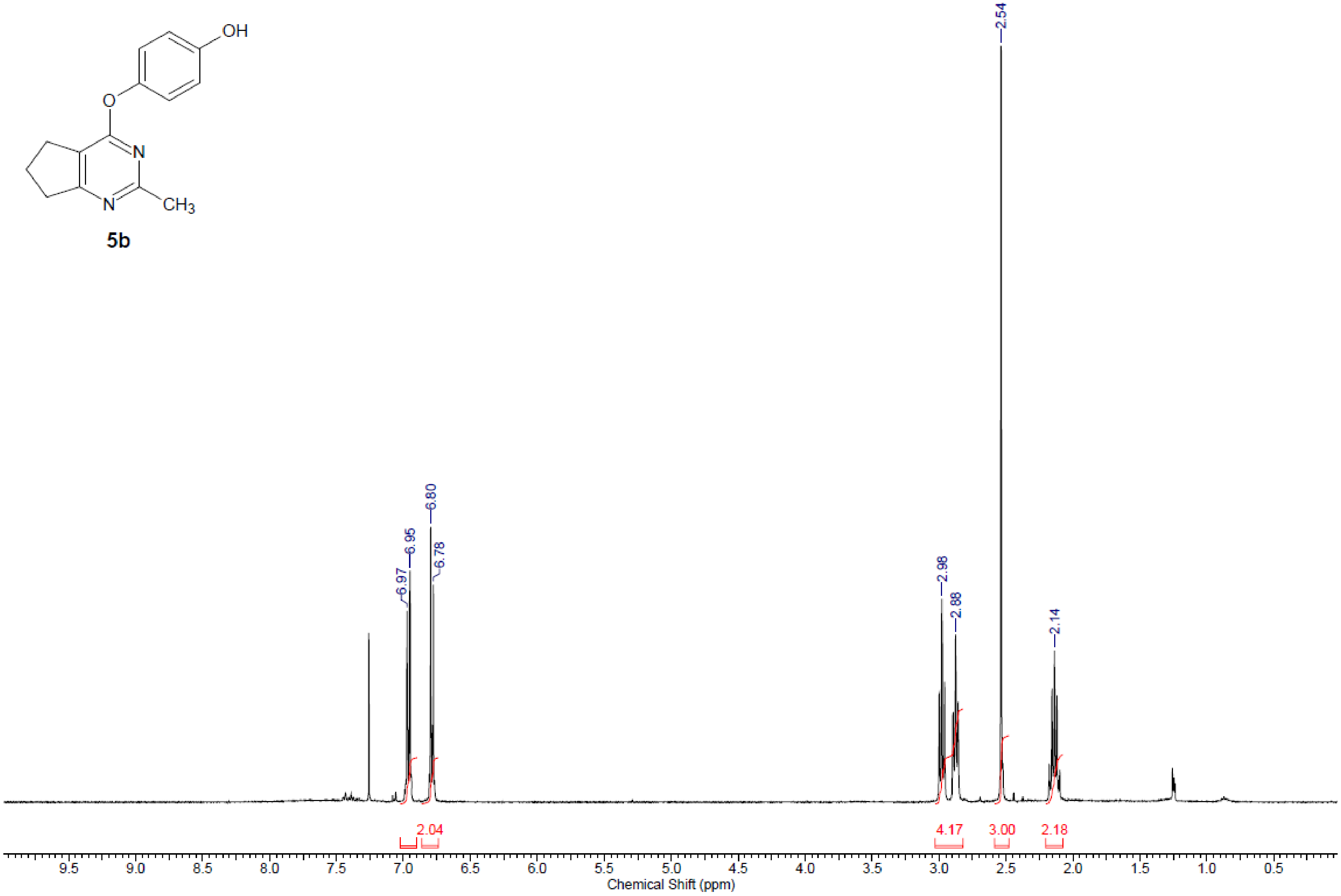

^{13}C NMR spectrum ($CDCl_3$) of compound **5b**

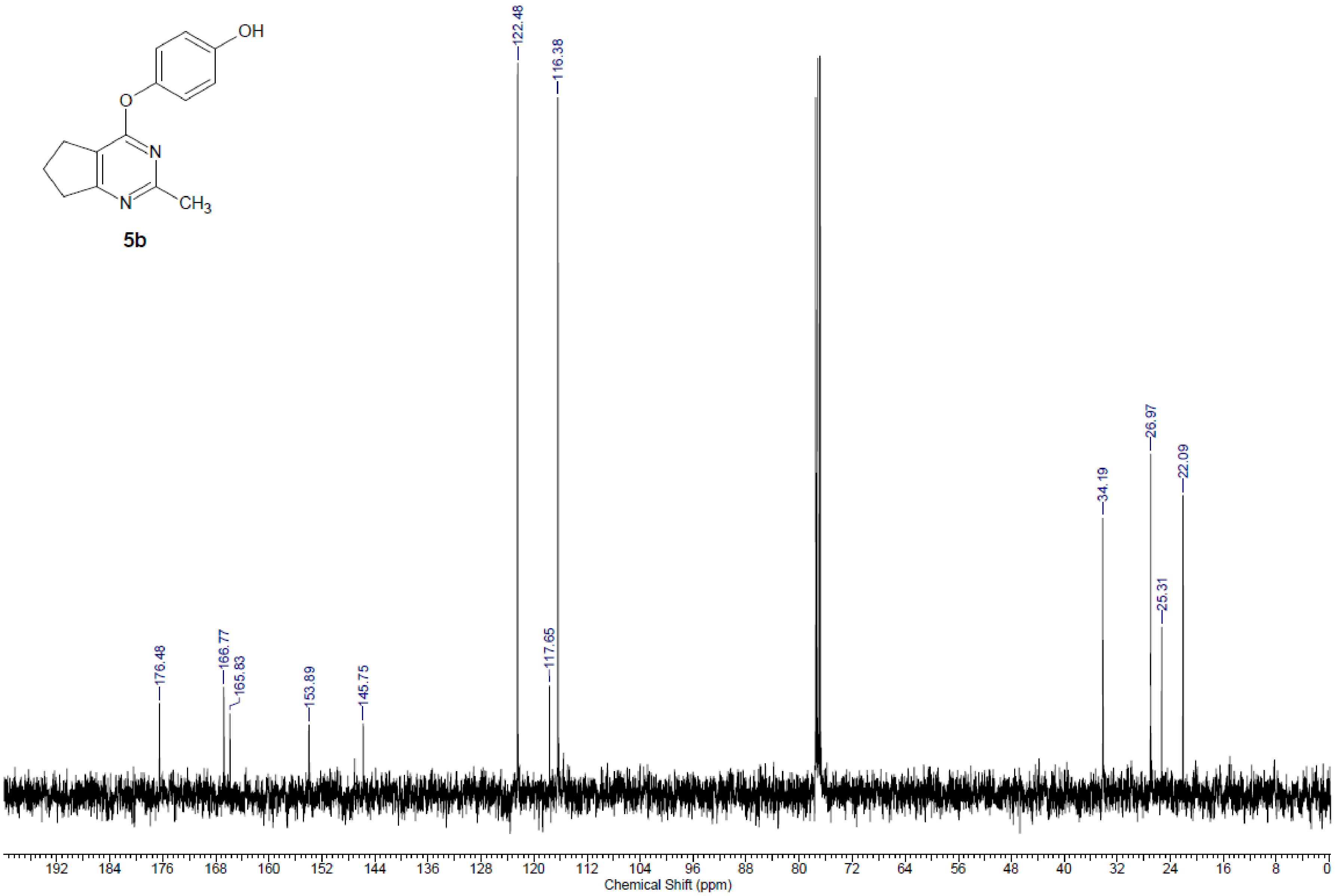

^{1}H NMR spectrum ($CDCl_3$) of compound **5c**

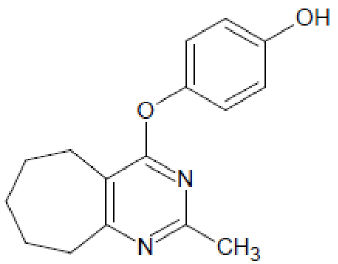

5c

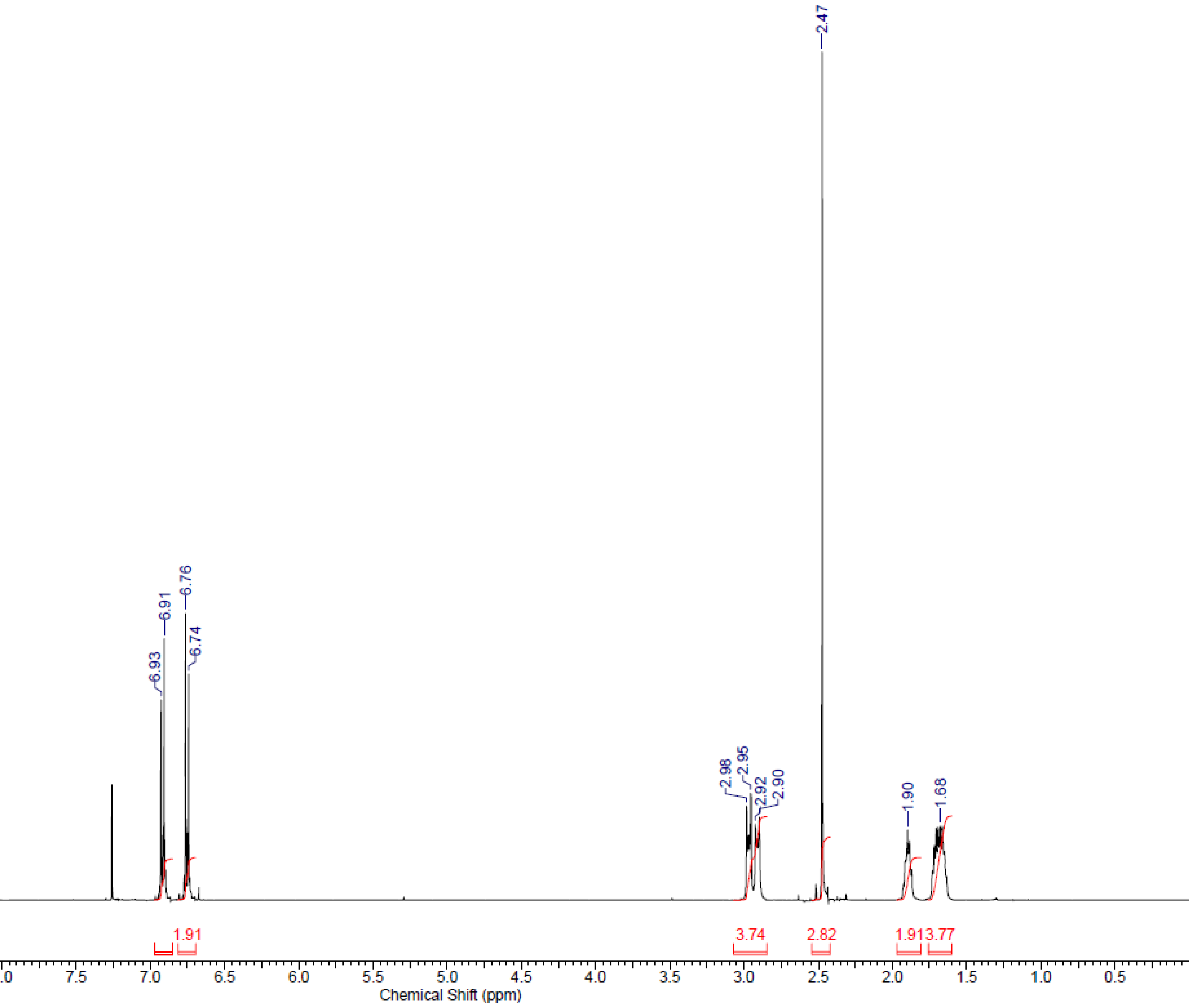

^{13}C NMR spectrum ($CDCl_3$) of compound **5c**

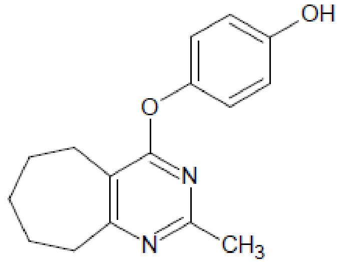

5c

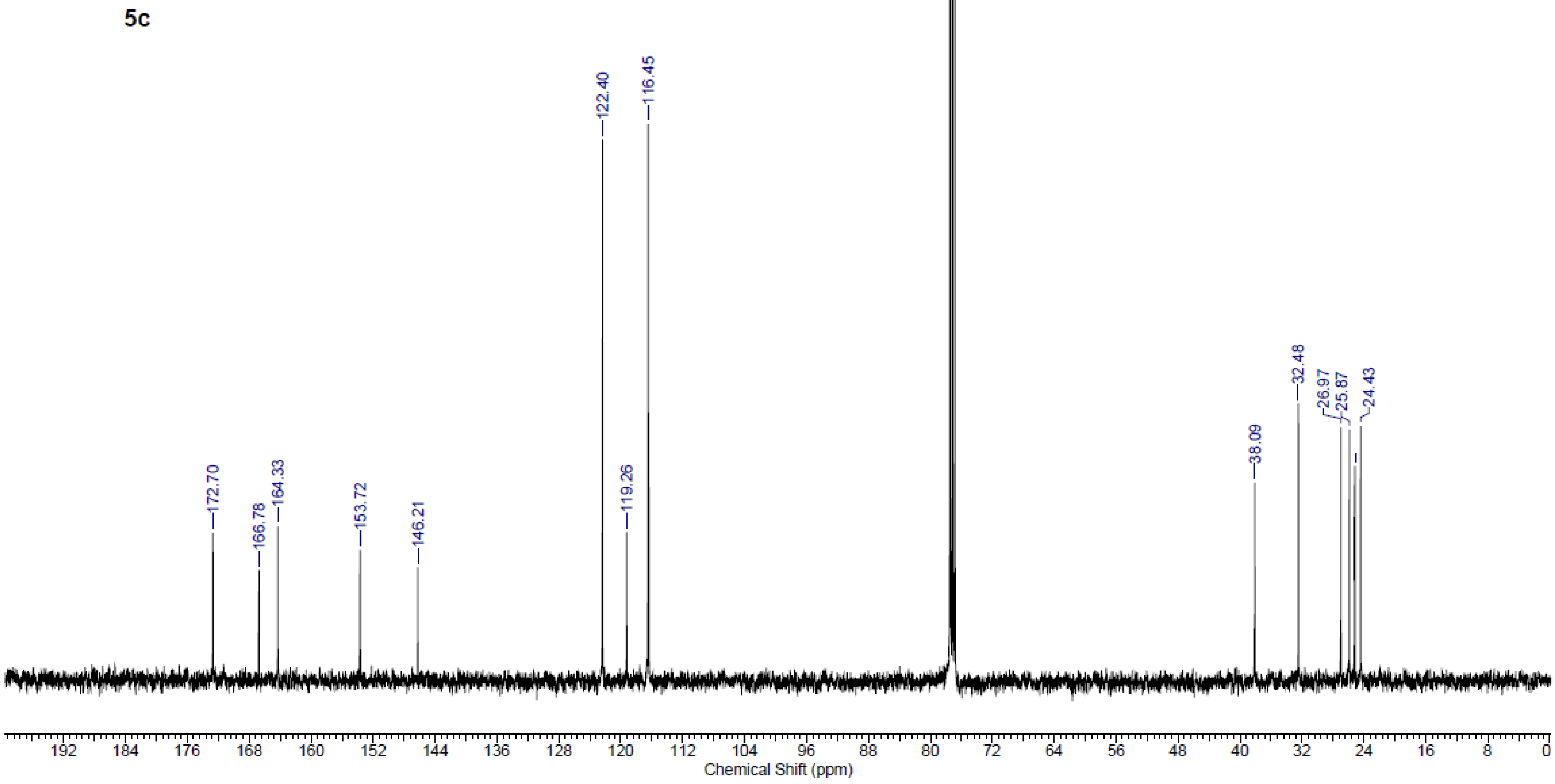

HSQC NMR spectrum ($CDCl_3$) of compound **5c**

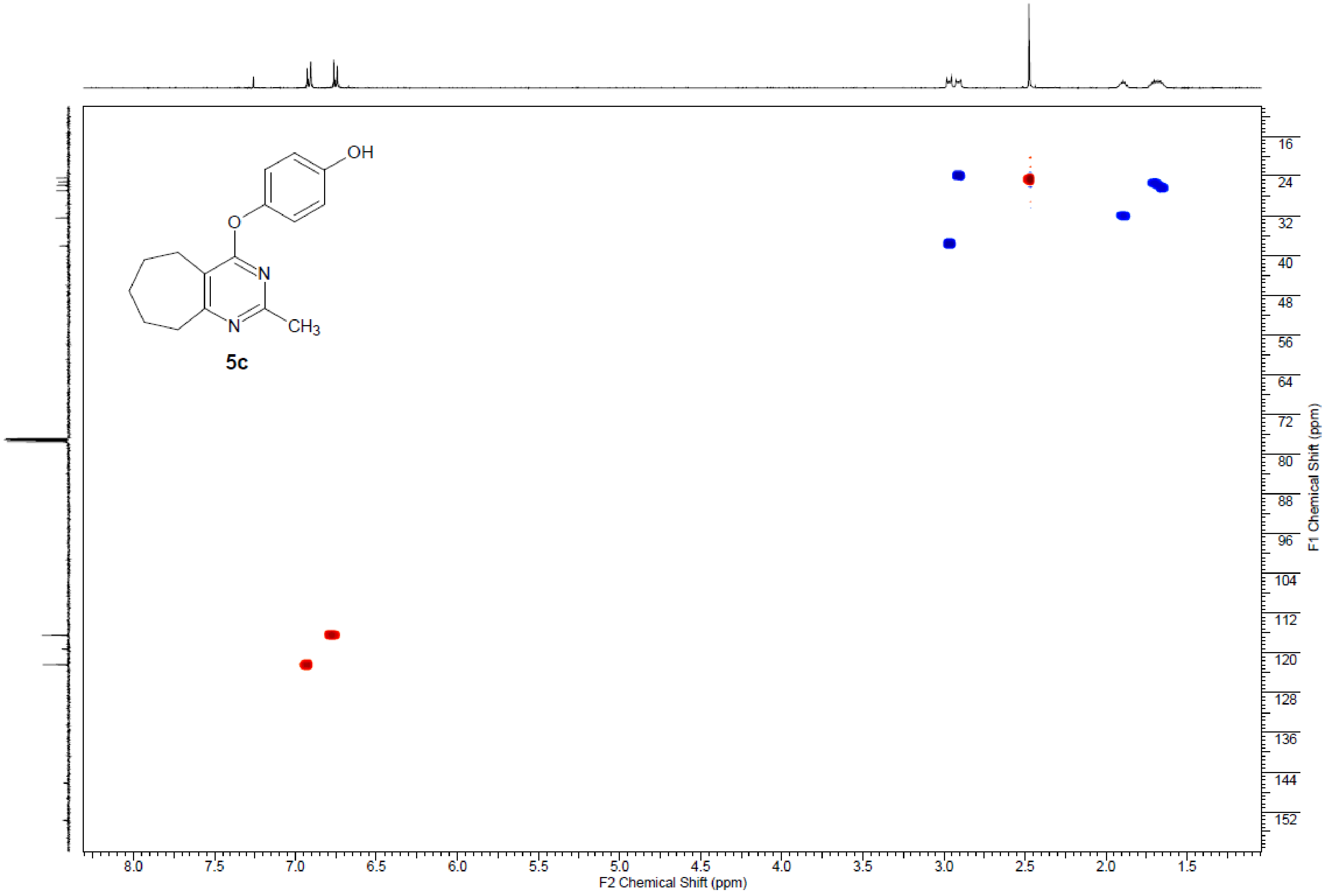

HMBC NMR spectrum ($CDCl_3$) of compound **5c**

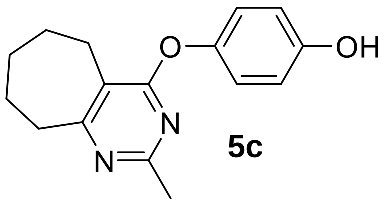

5c

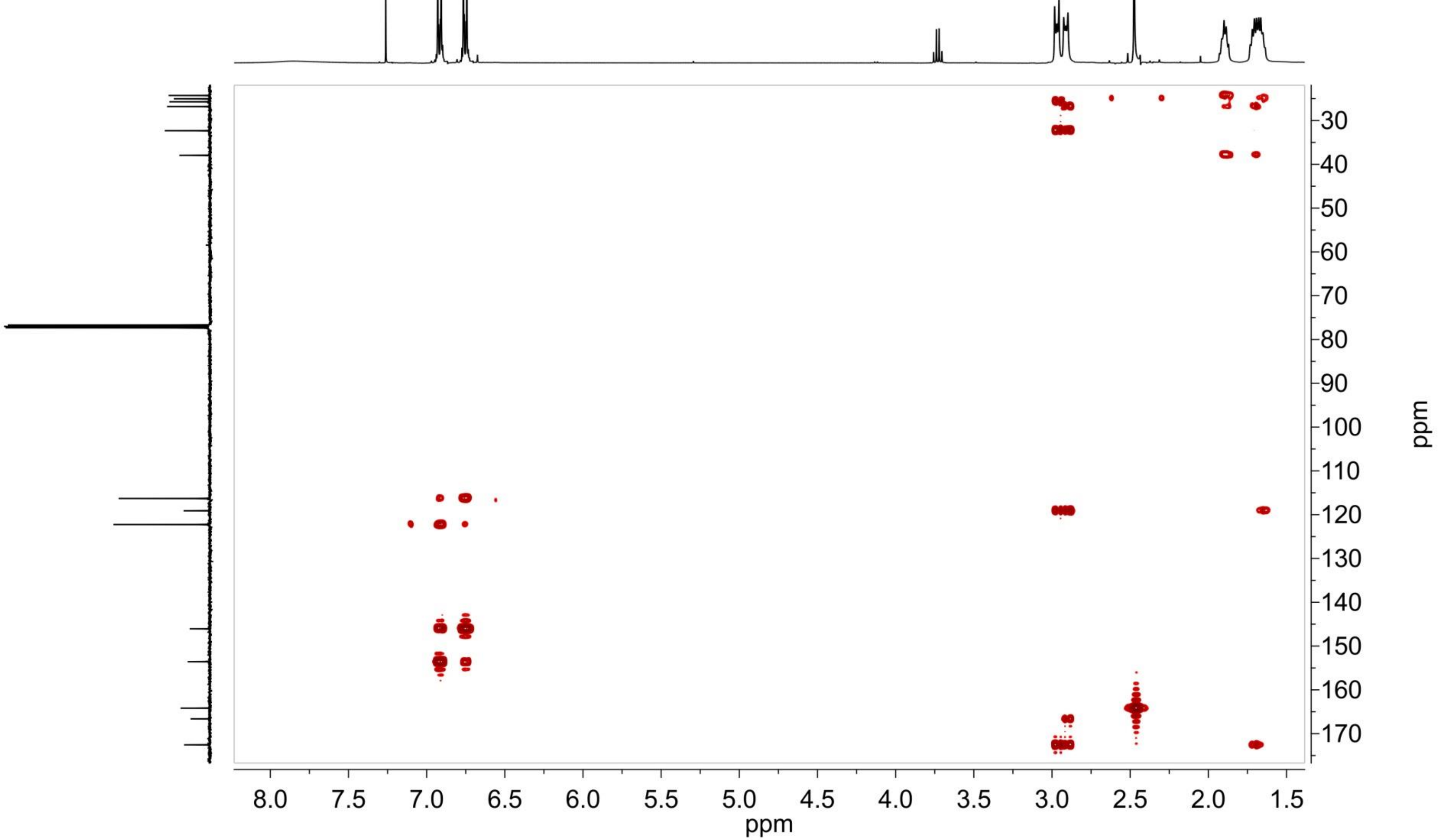

^{1}H NMR spectrum ($CDCl_3$) of compound **5d**

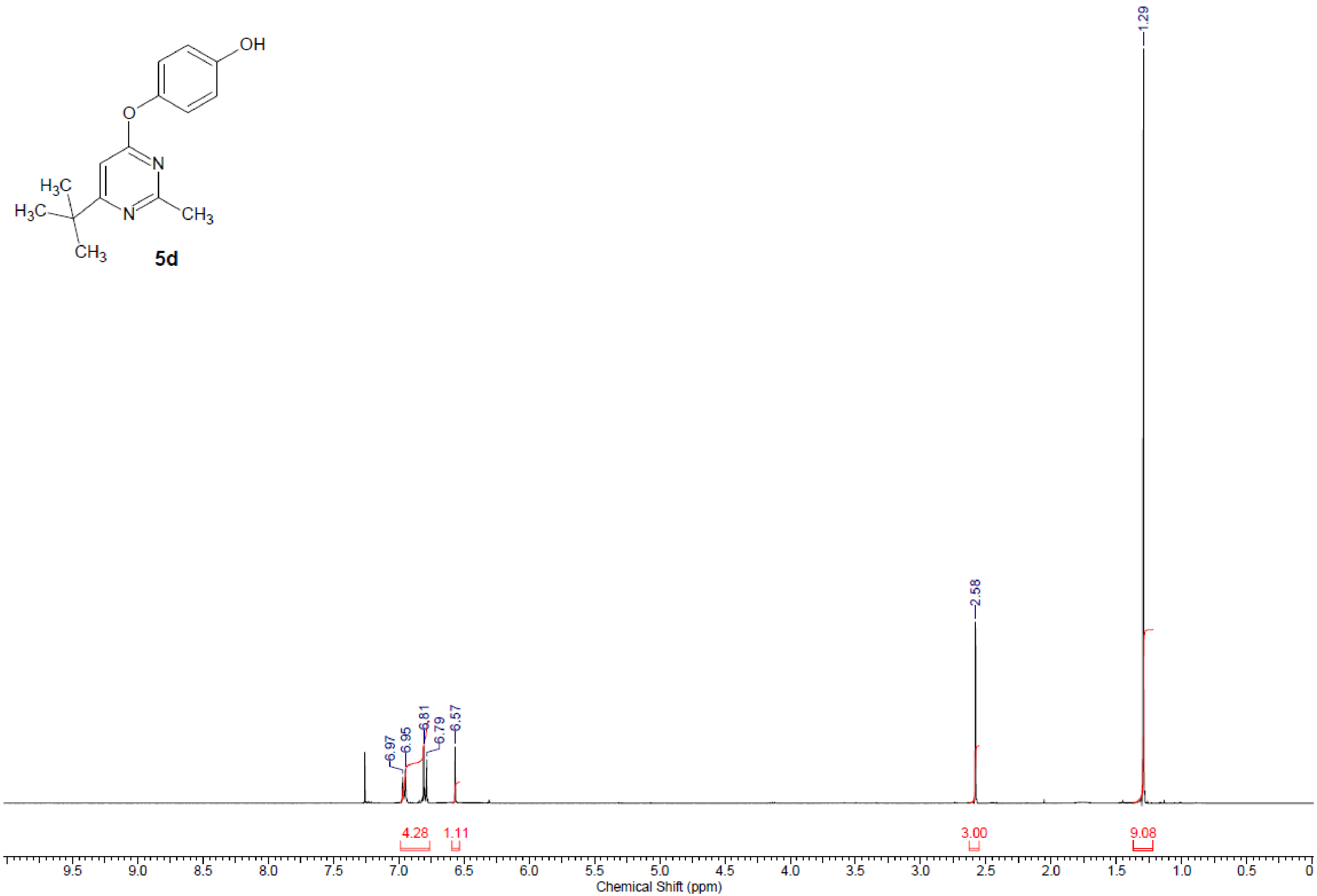

^{13}C NMR spectrum ($CDCl_3$+CD_3OD) of compound **5d**

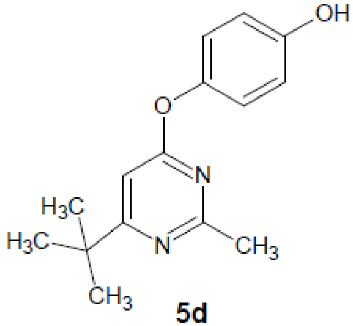

5d

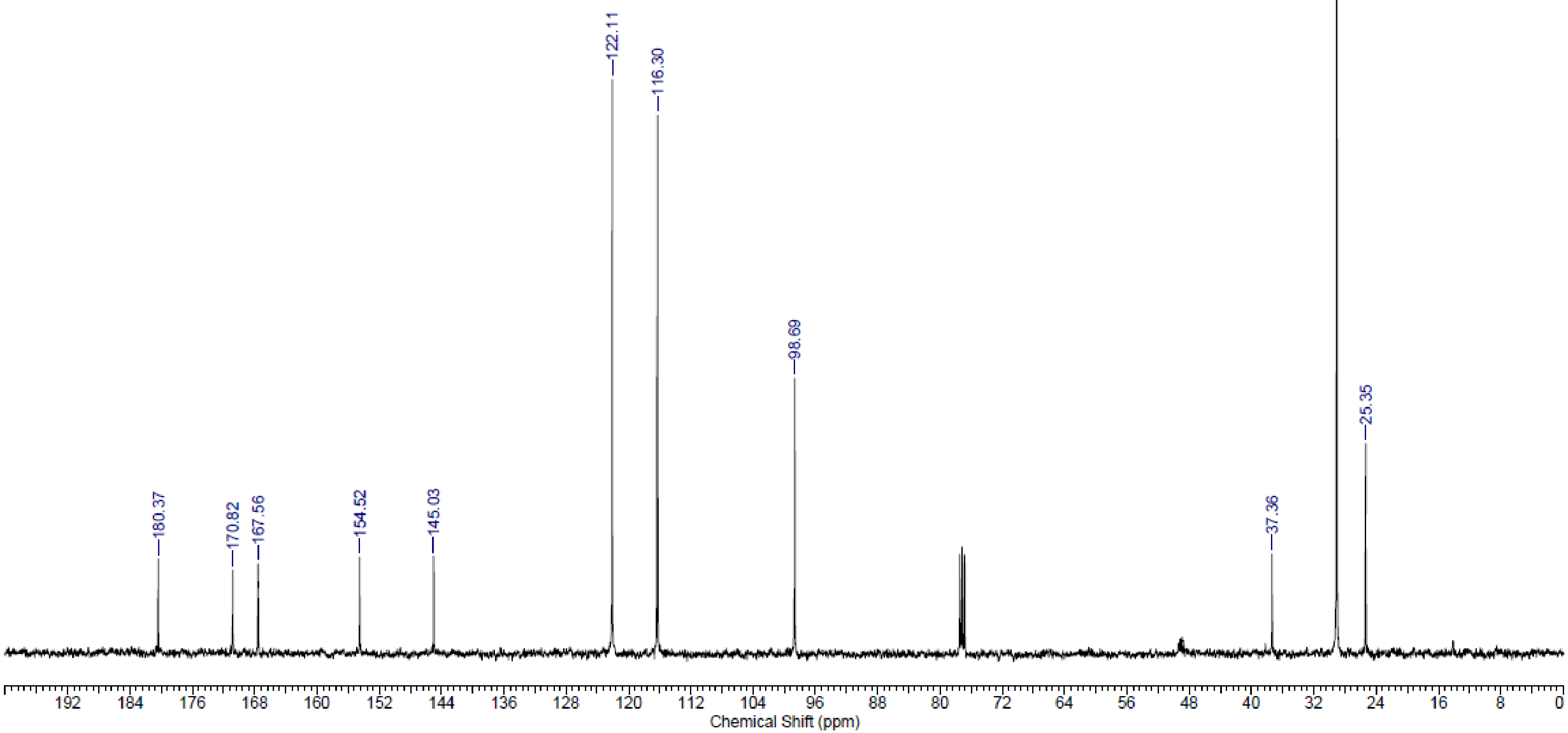

HSQC NMR spectrum ($CDCl_3$) of compound **5d**

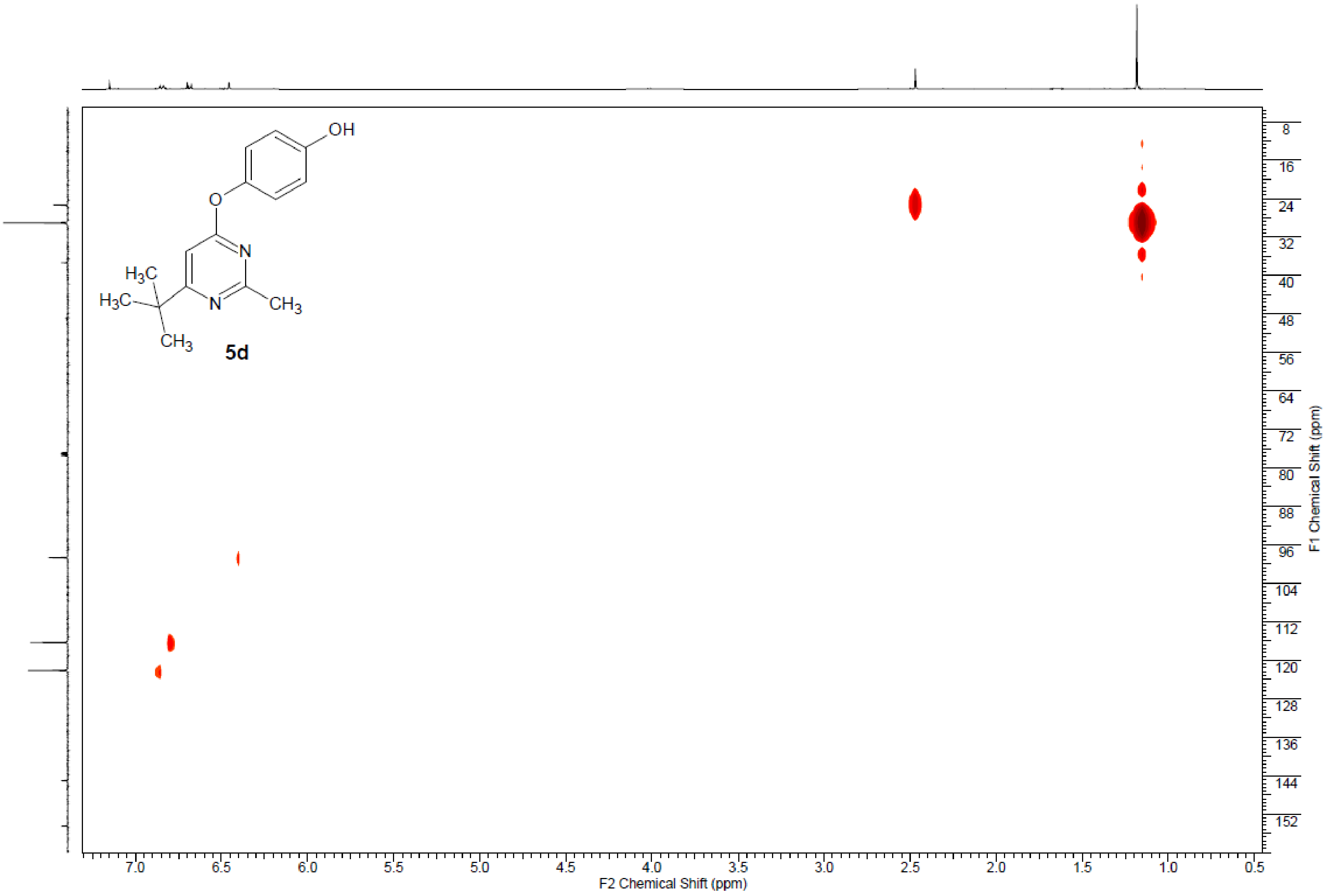

^{1}H NMR spectrum ($CDCl_3$) of compound **5e**

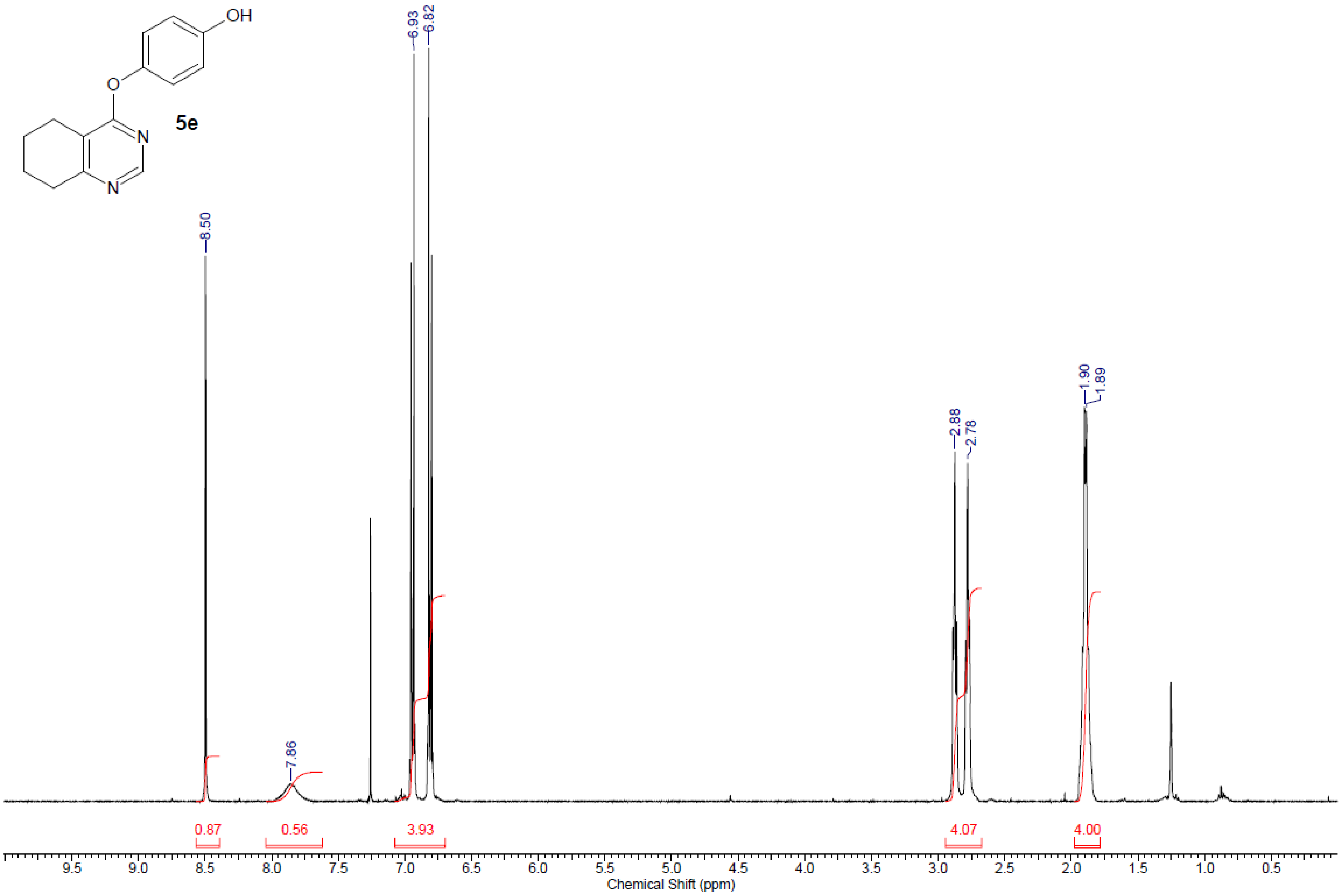

^{13}C NMR spectrum ($CDCl_3$) of compound **5e**

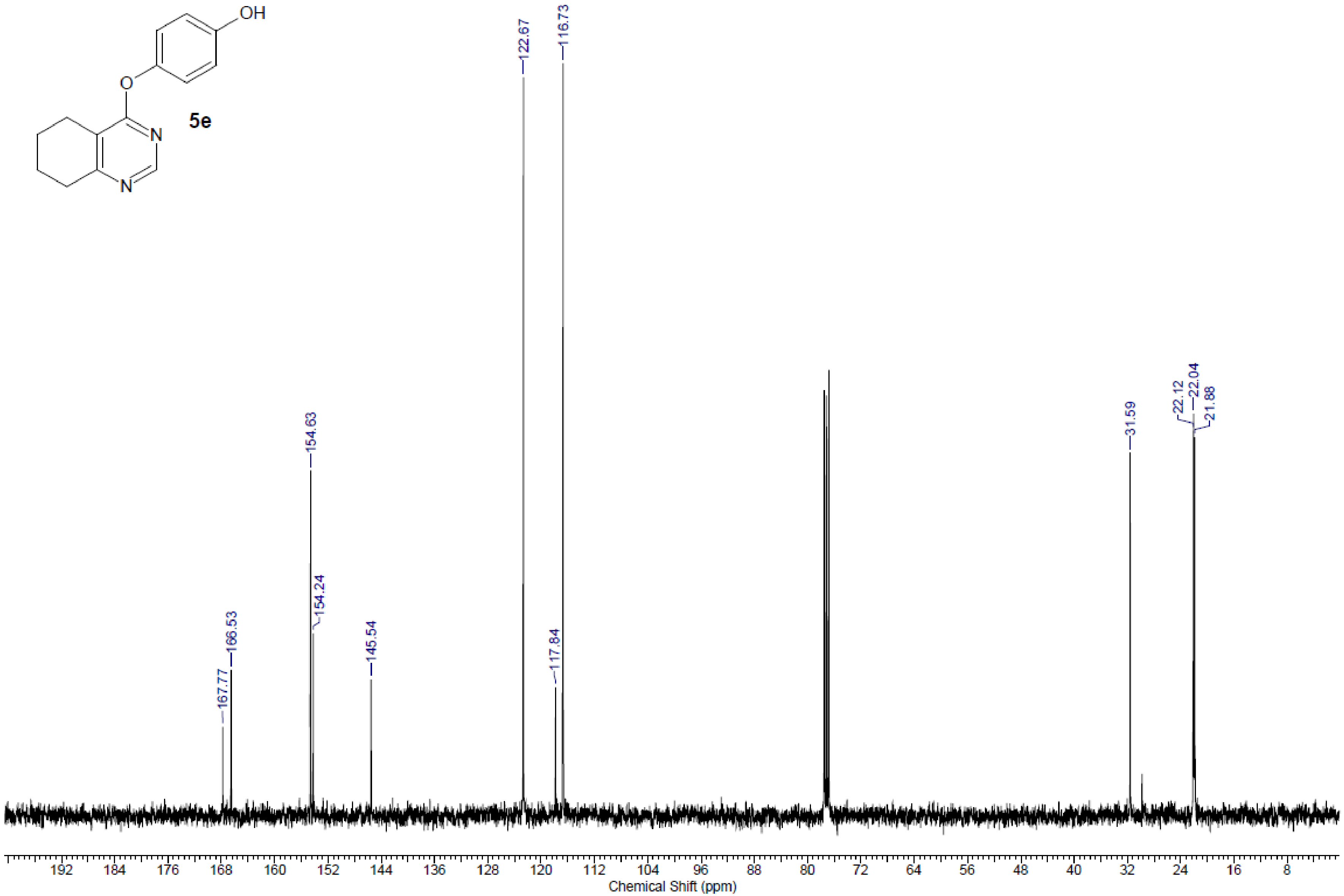

5e

^{1}H NMR spectrum ($CDCl_3$) of compound **8**

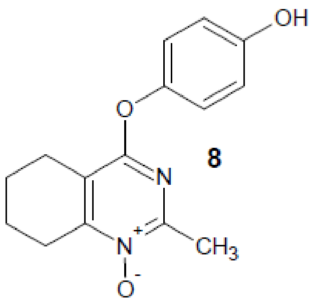

8

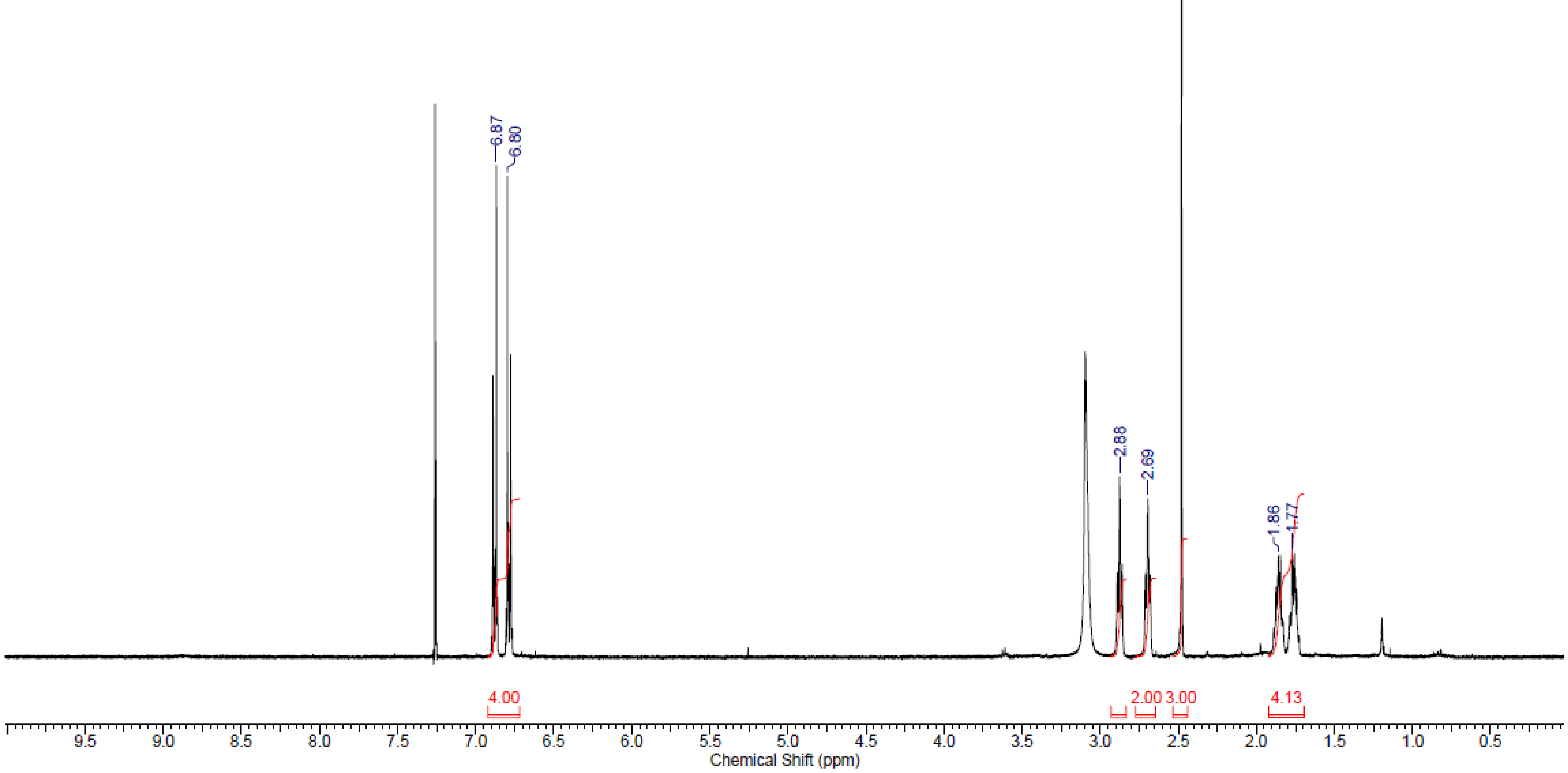

^{1}H NMR spectrum ($CDCl_3$) of compound **1a**

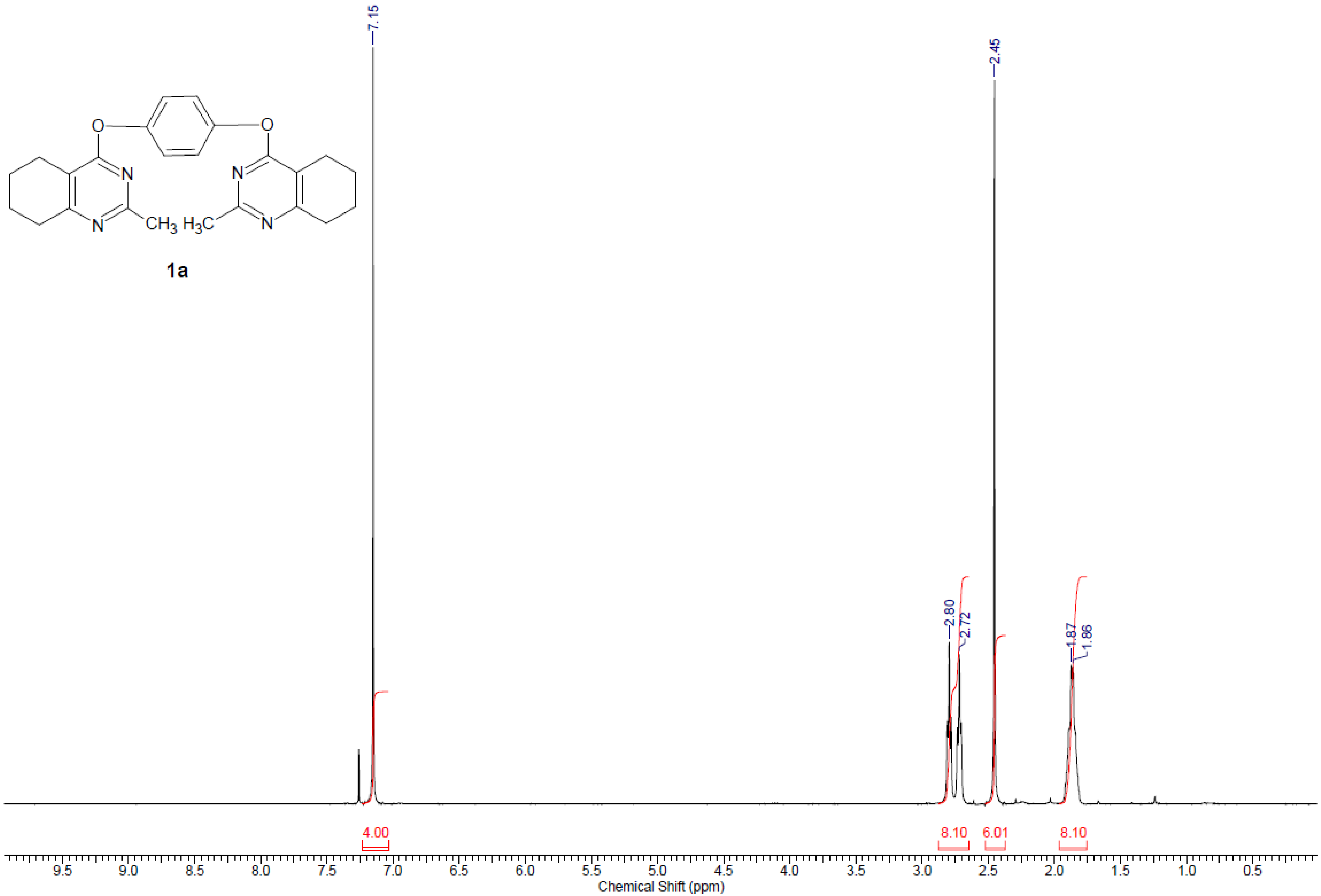

^1H NMR spectrum ($CDCl_3$) of compound **1f**

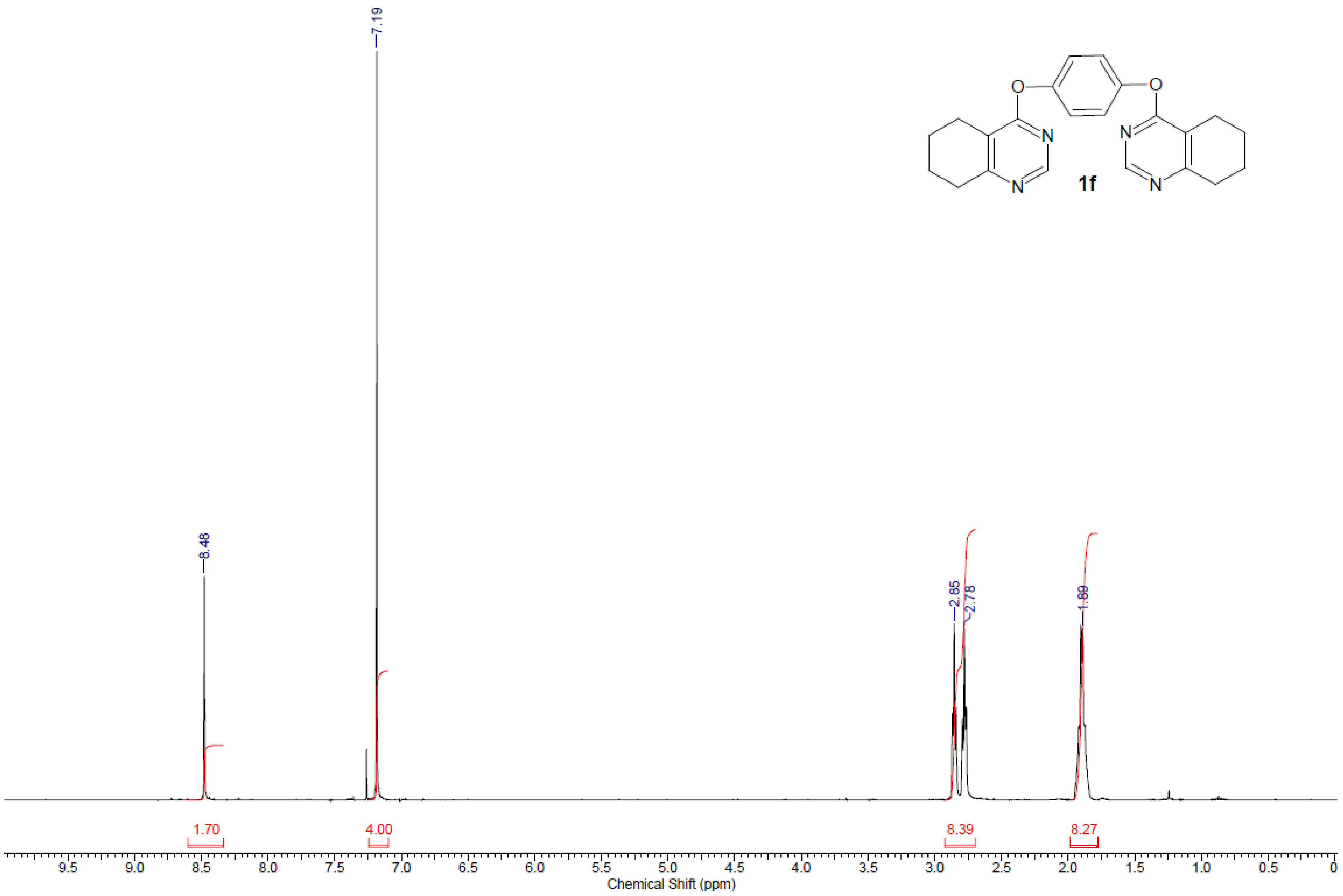

^{13}C NMR spectrum ($CDCl_3$) of compound **1f**

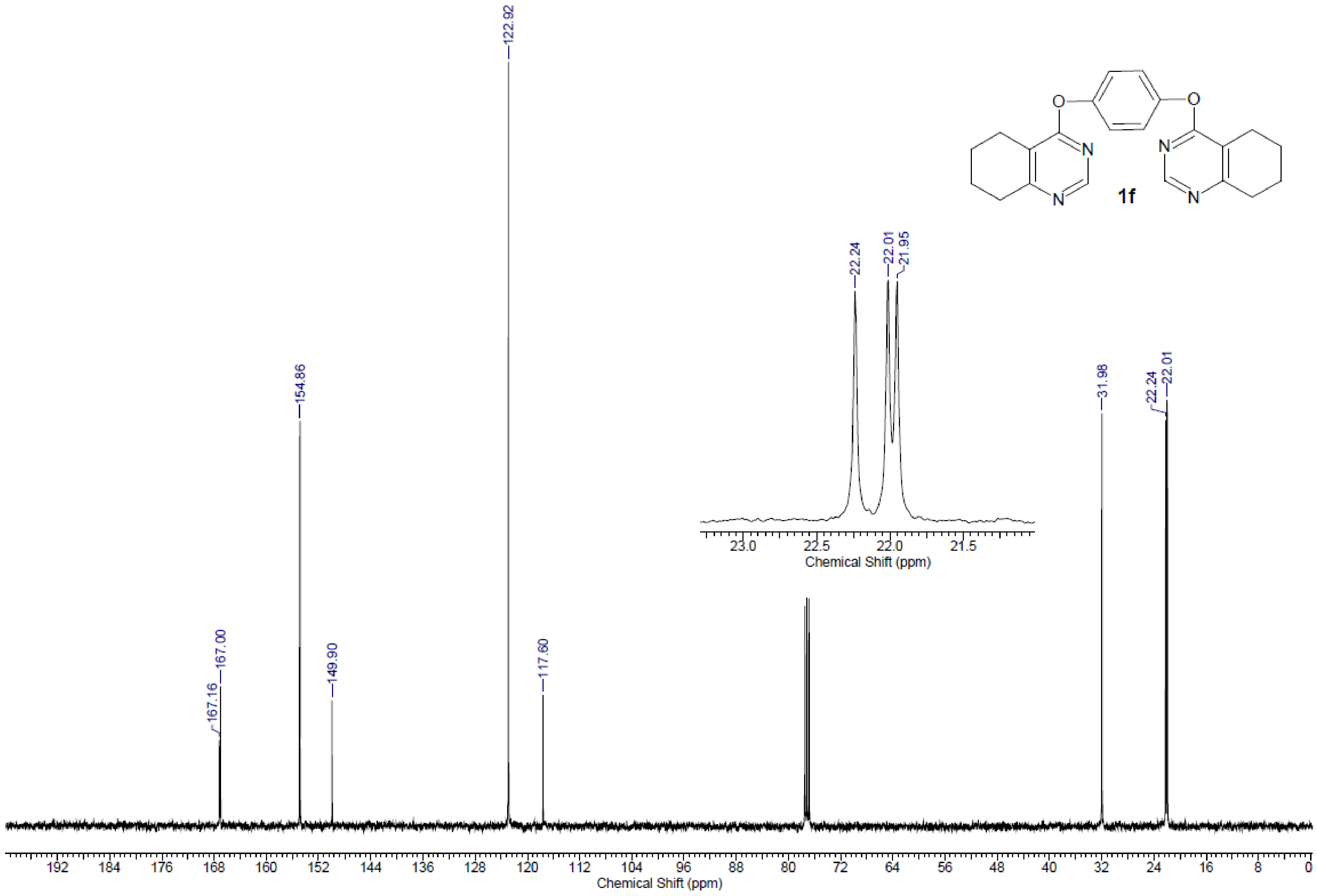

HSQC NMR spectrum ($CDCl_3$) of compound **1f**

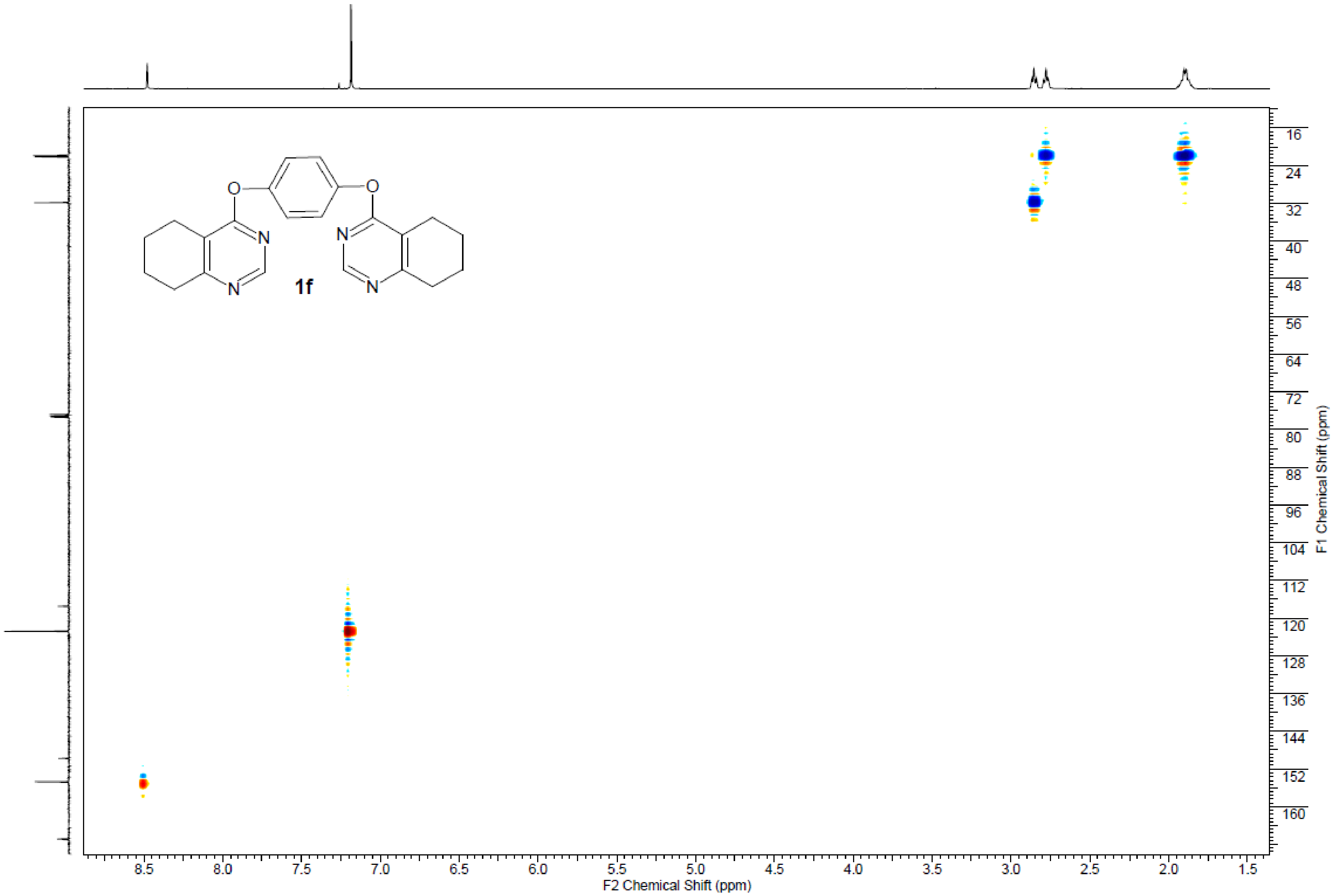

HMBC NMR spectrum ($CDCl_3$) of compound **1f**

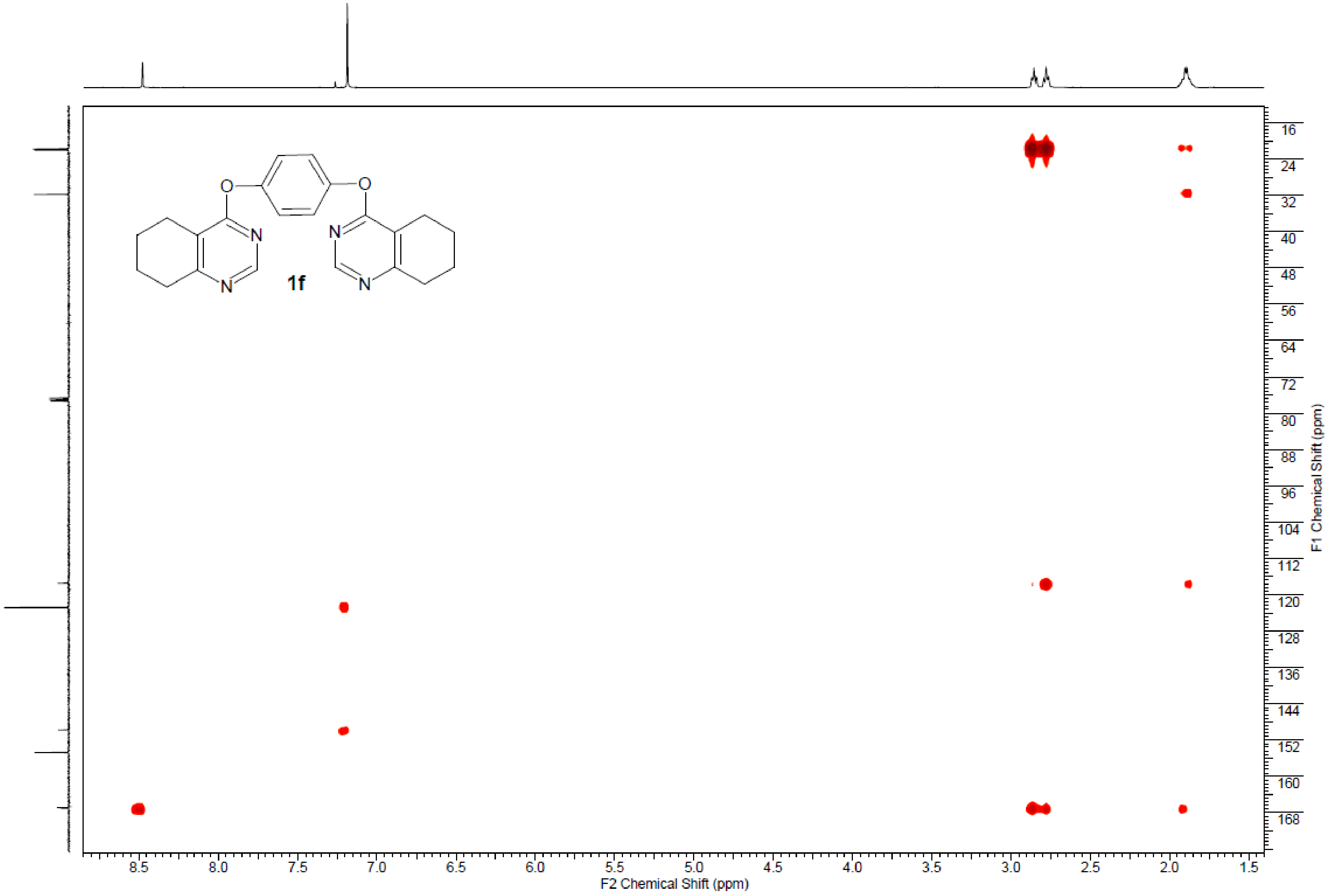

^{1}H NMR spectrum ($CDCl_3$) of compound **1g**

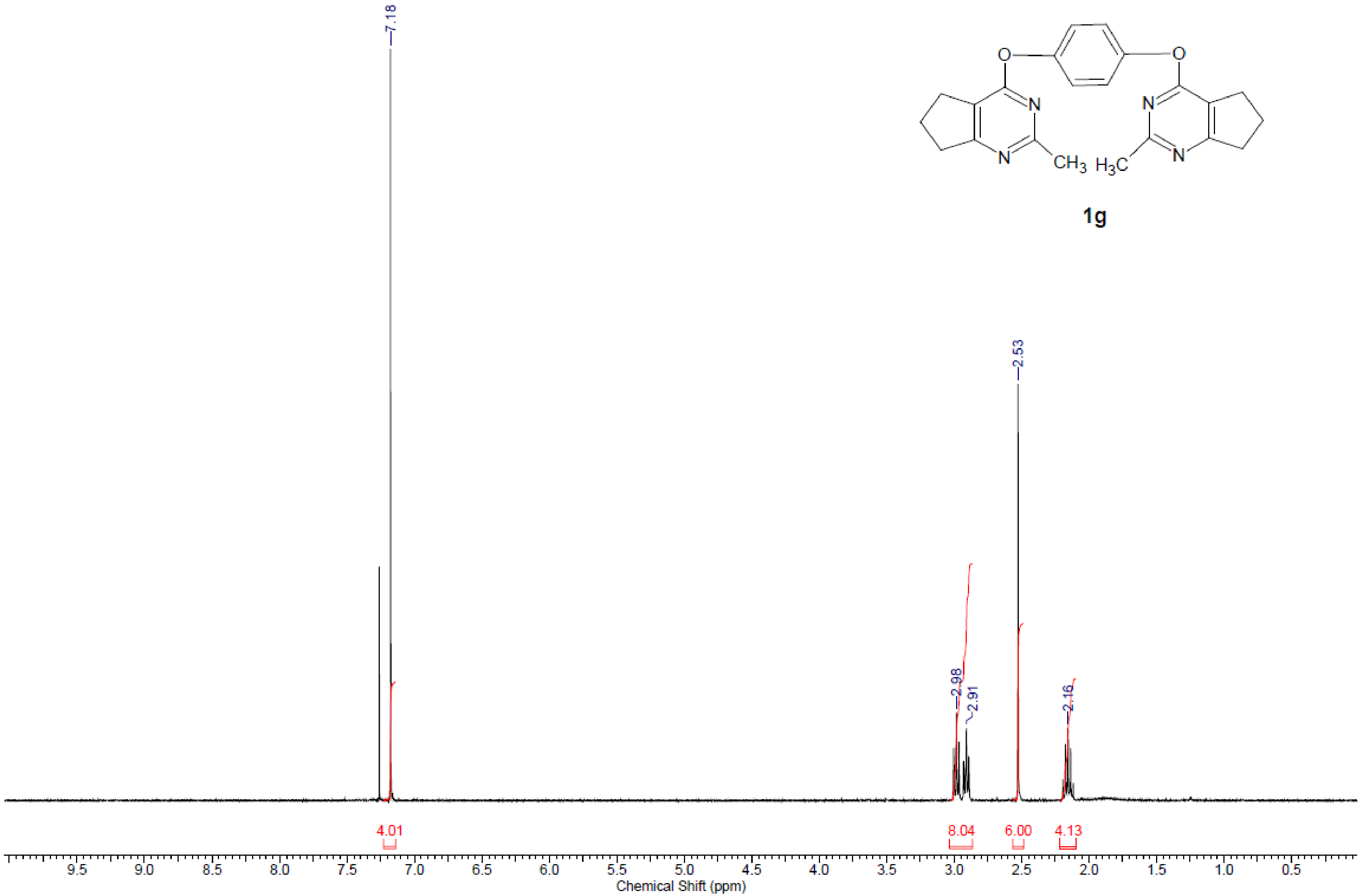

^{13}C NMR spectrum ($CDCl_3$) of compound **1g**

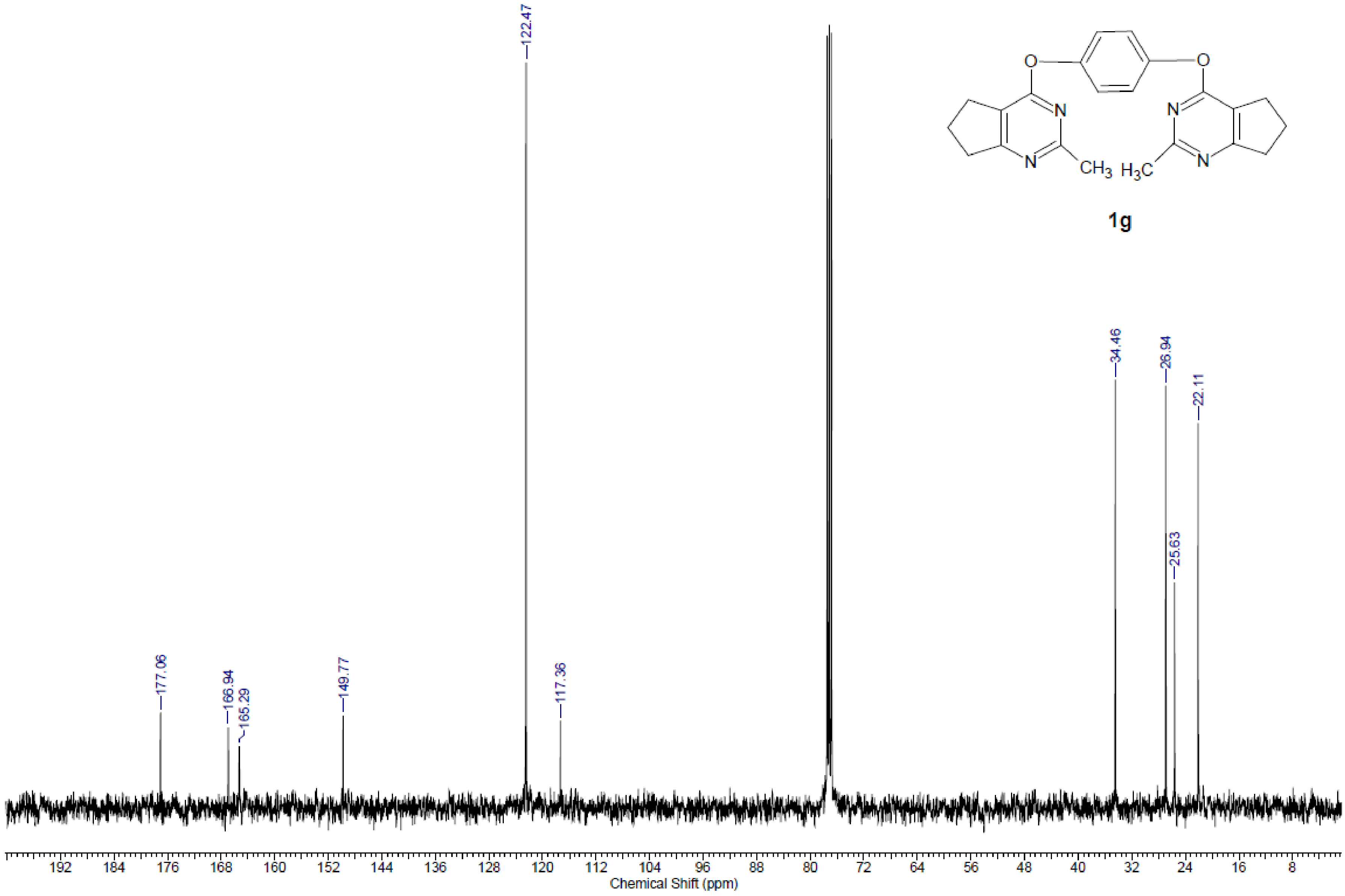

^{1}H NMR spectrum ($CDCl_3$) of compound **1h**

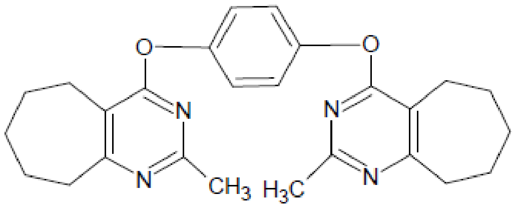

1h

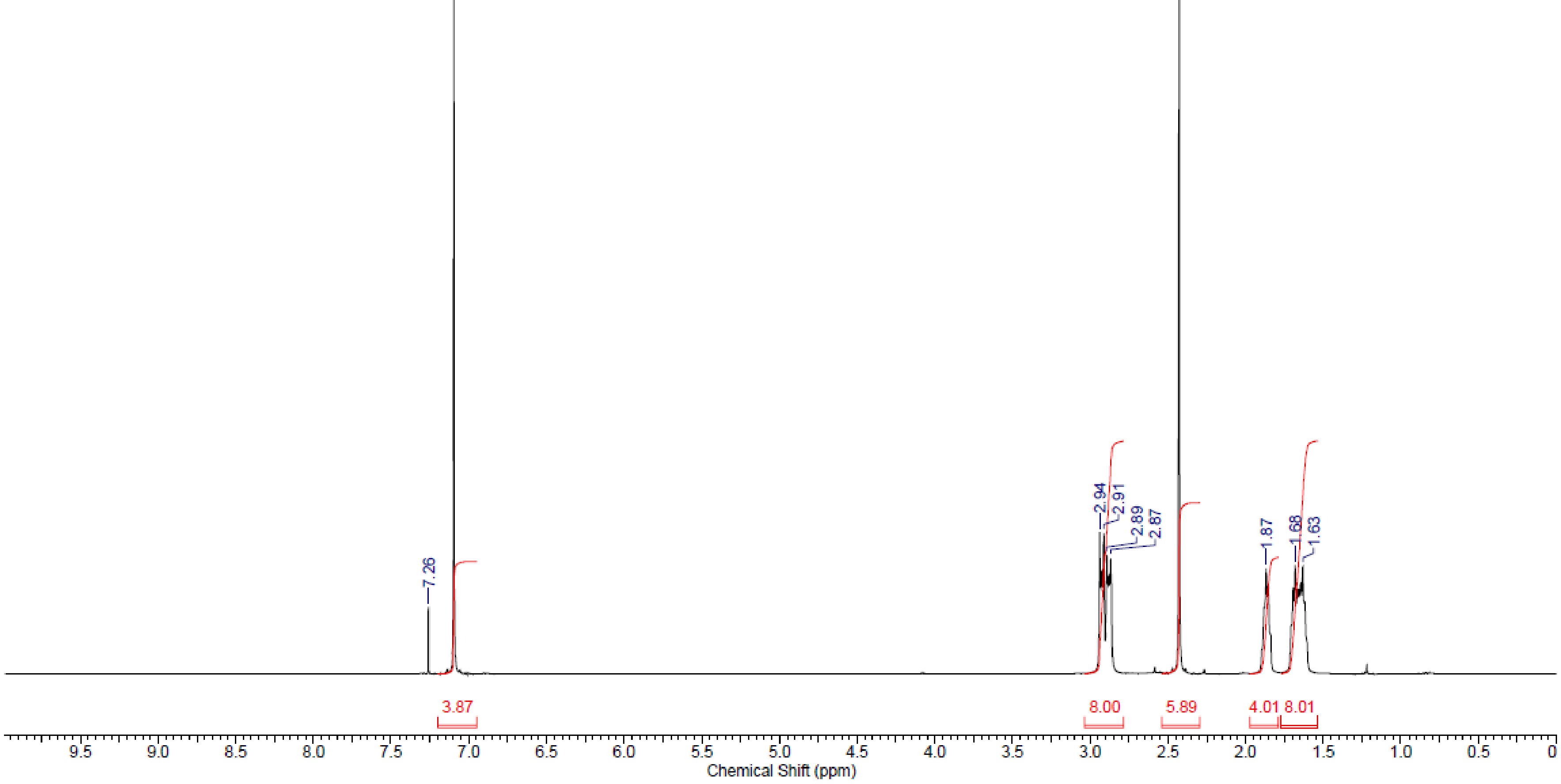

^{13}C NMR spectrum ($CDCl_3$) of compound **1h**

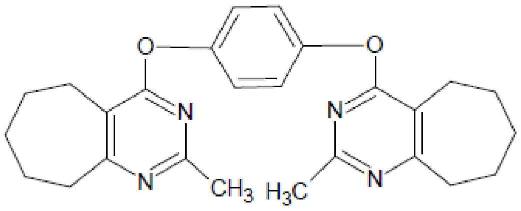

1h

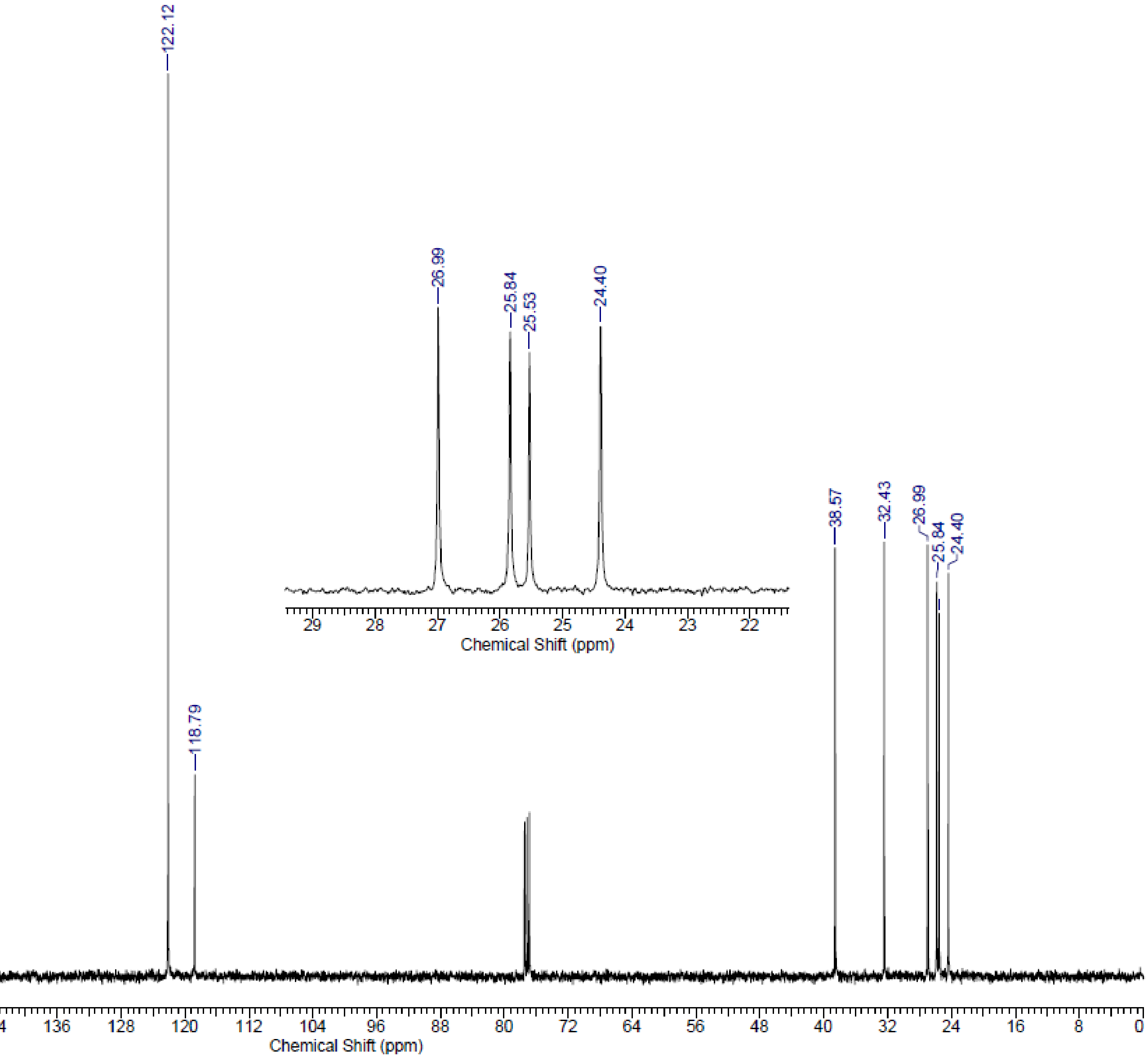

HSQC NMR spectrum ($CDCl_3$) of compound **1h**

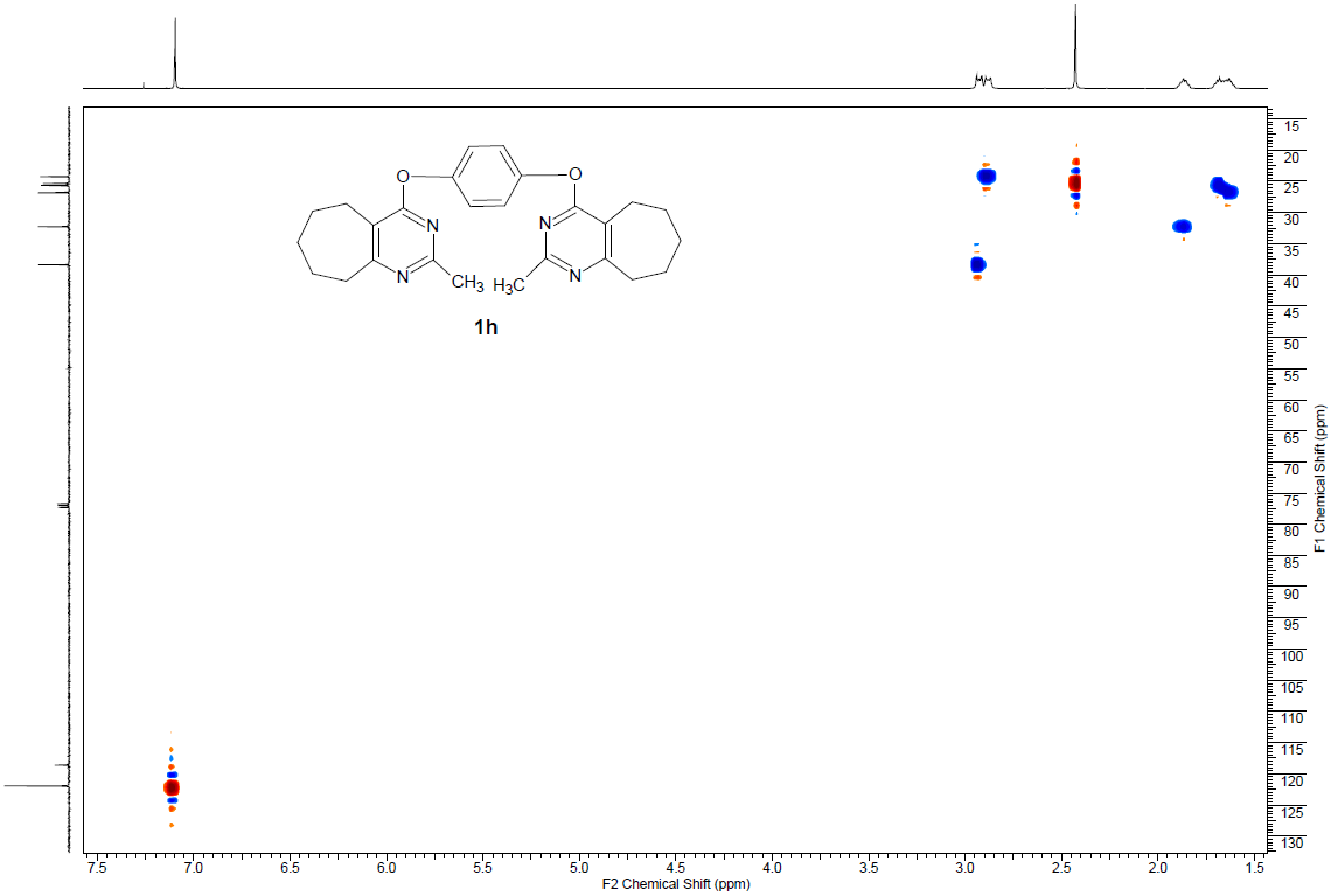

HMBC NMR spectrum ($CDCl_3$) of compound **1h**

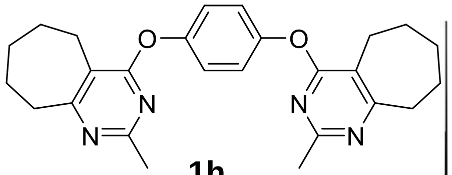

1h

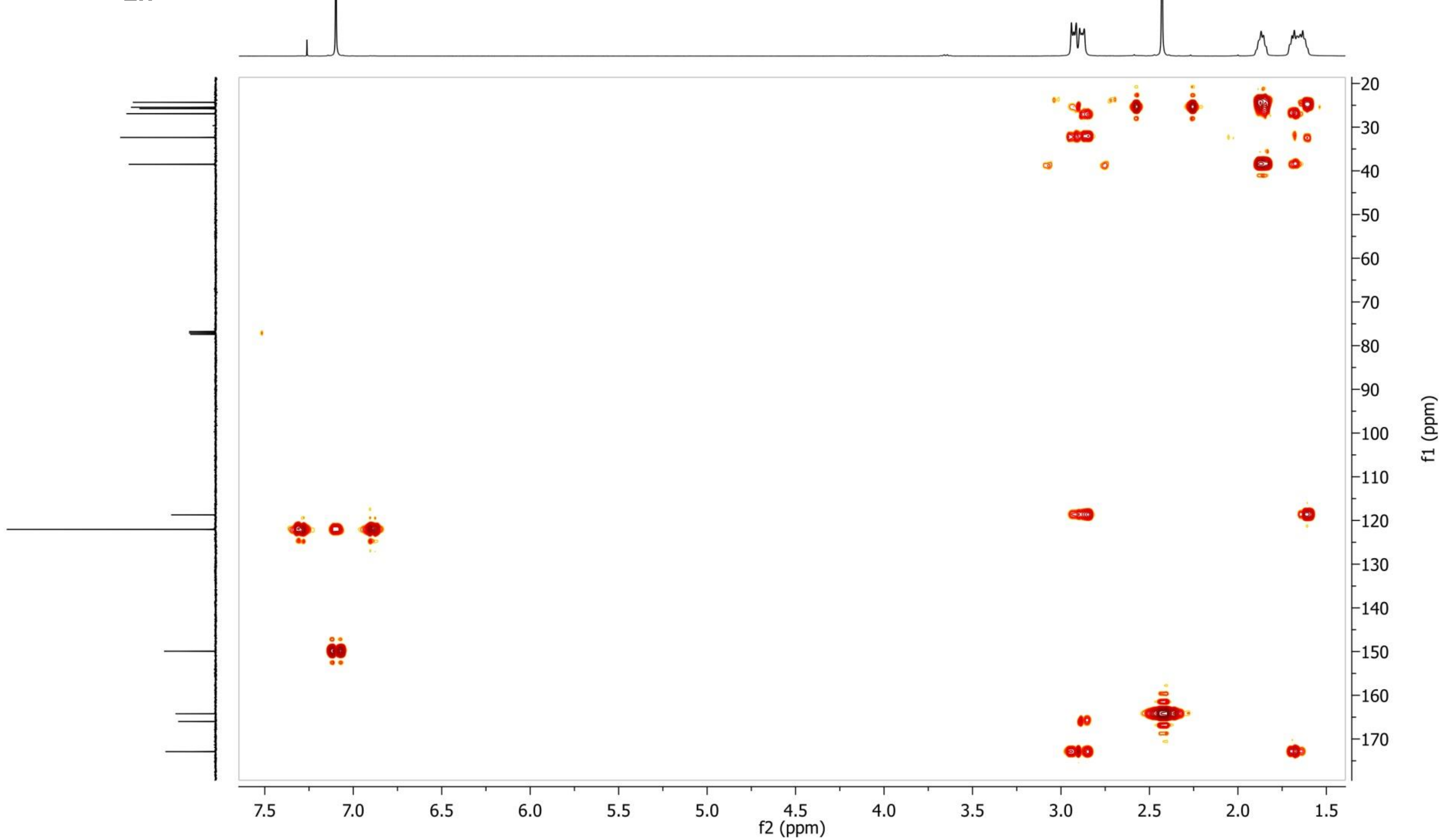

^{1}H NMR spectrum ($CDCl_3$) of compound **1i**

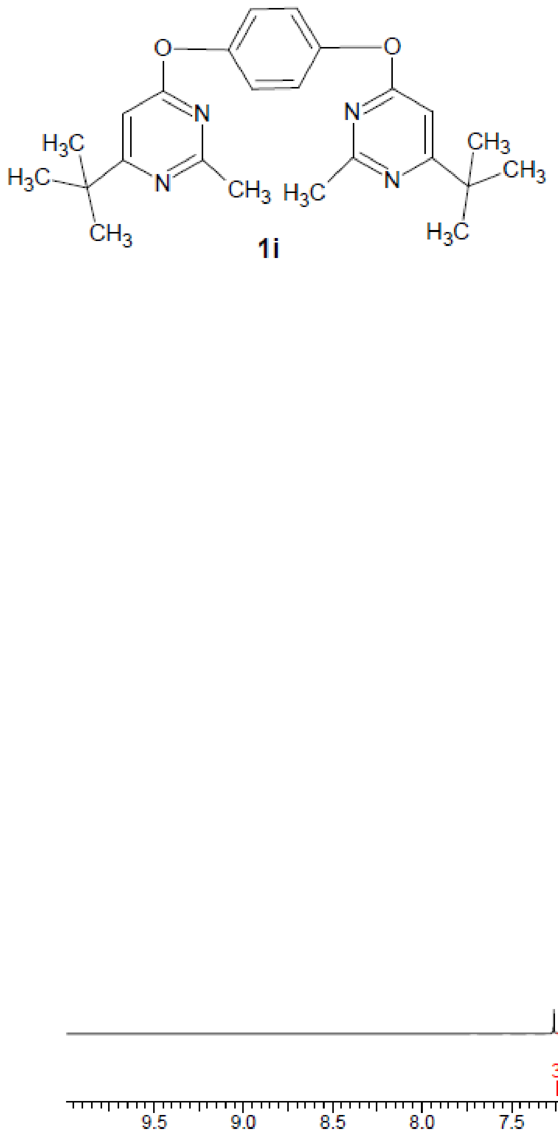

1i

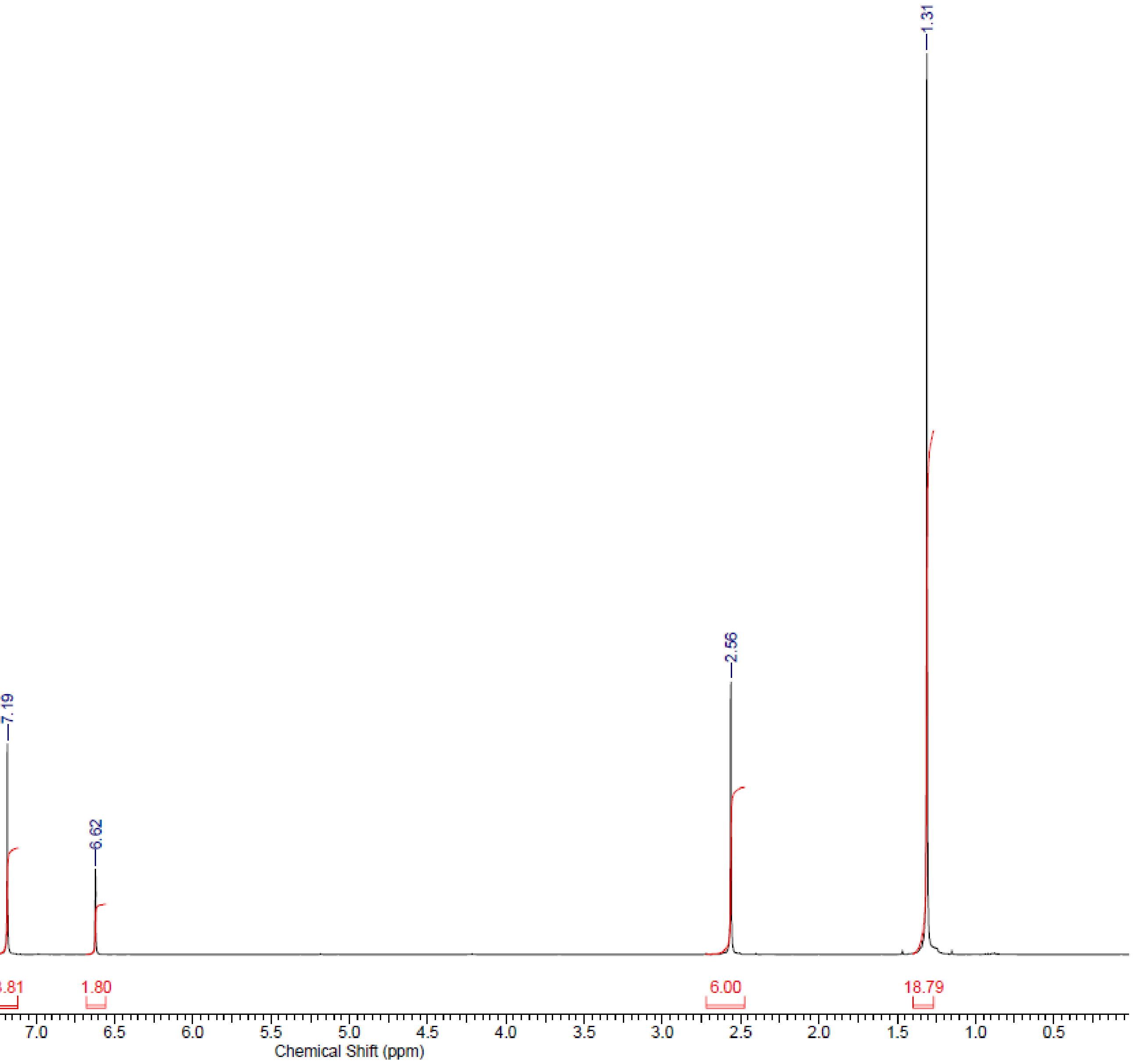

^{13}C NMR spectrum ($CDCl_3$) of compound **1i**

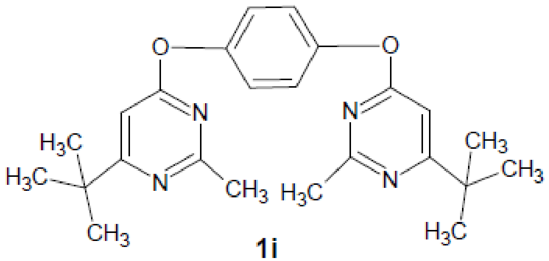

1i

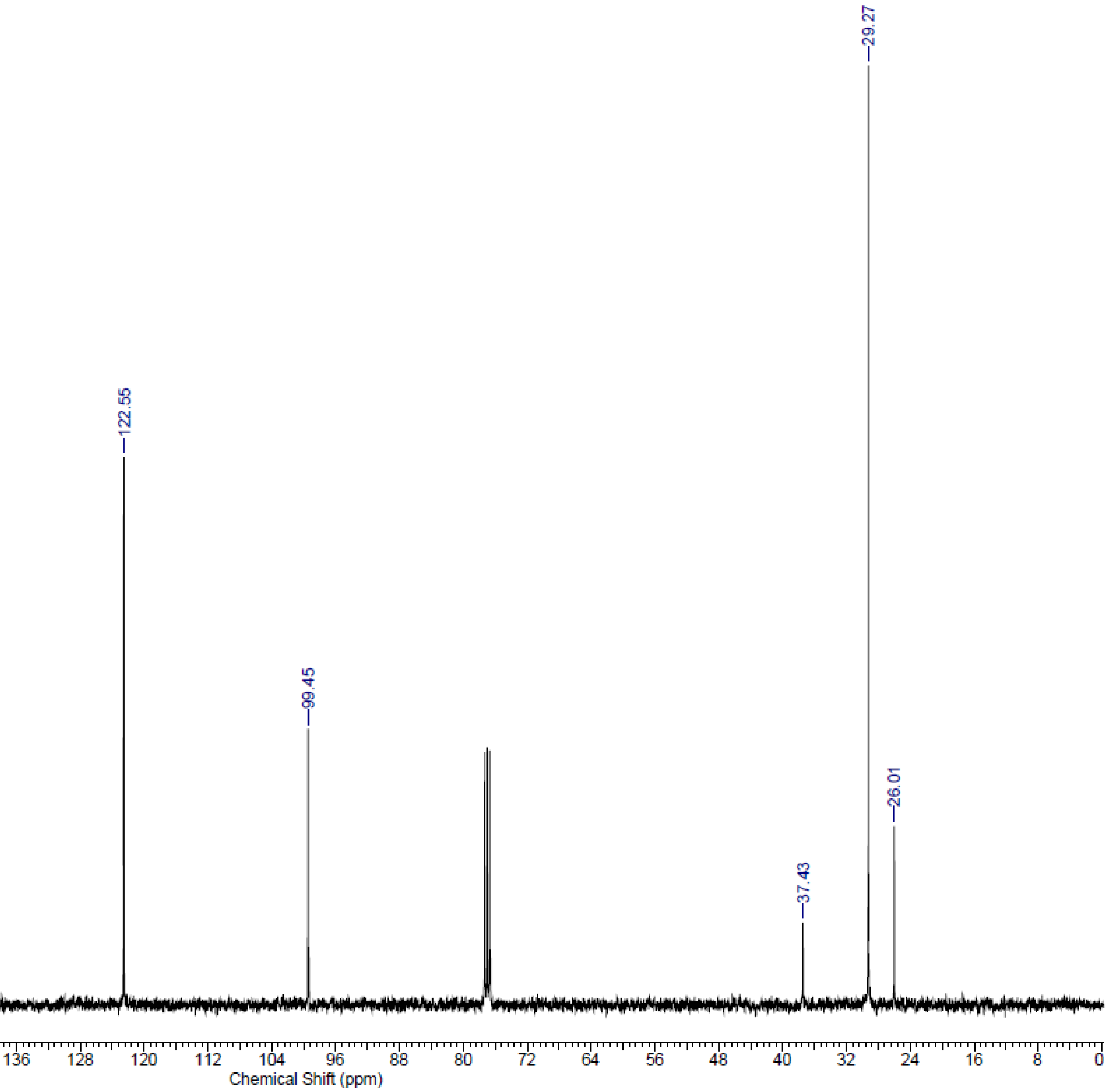

HMBC NMR spectrum ($CDCl_3$) of compound **1i**

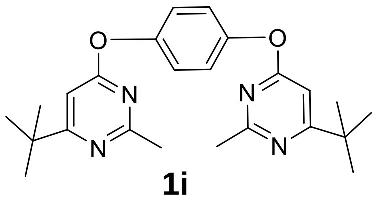

1i

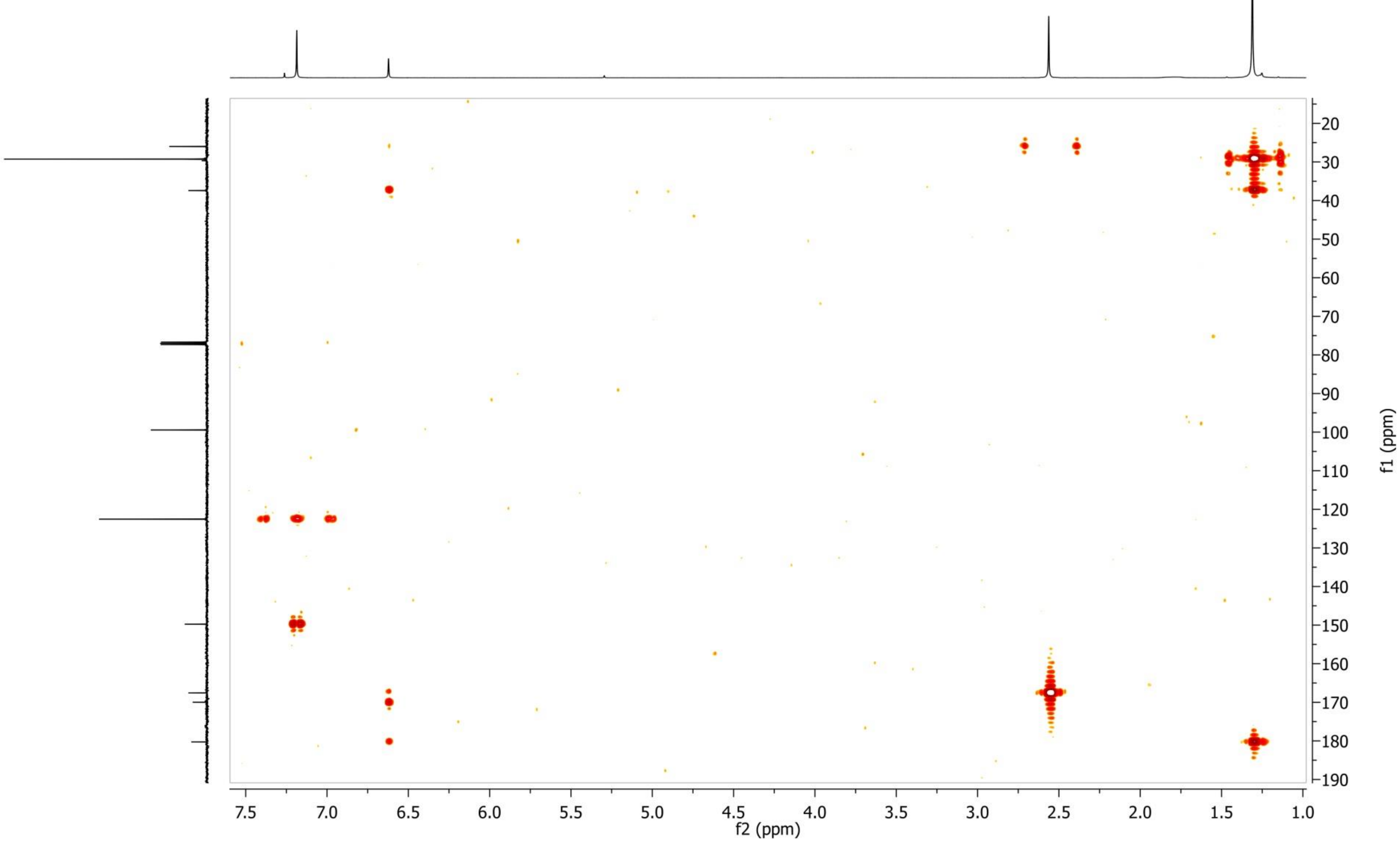

^{1}H NMR spectrum ($CDCl_3$) of compound **1j**

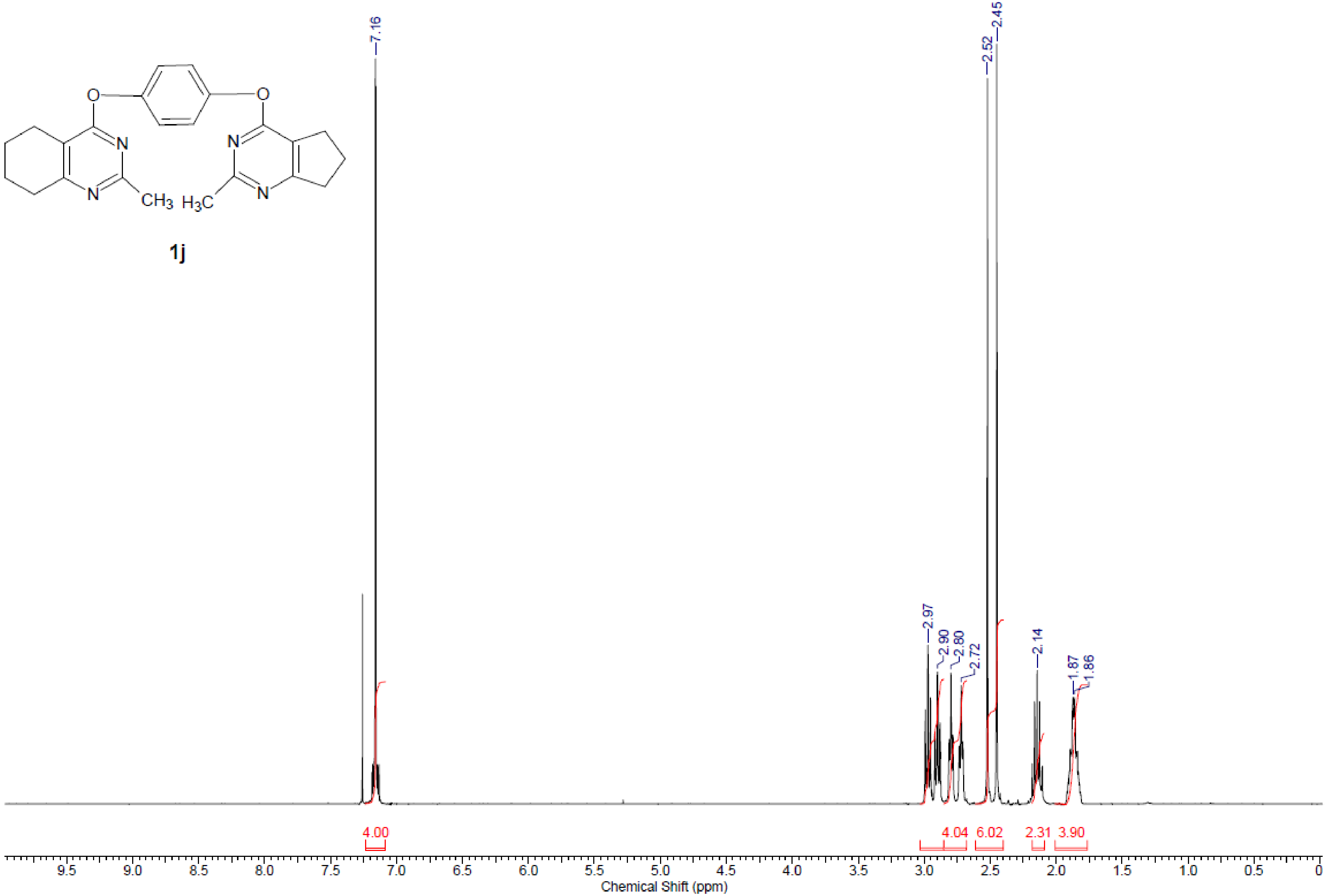

^{13}C NMR spectrum ($CDCl_3$) of compound **1j**

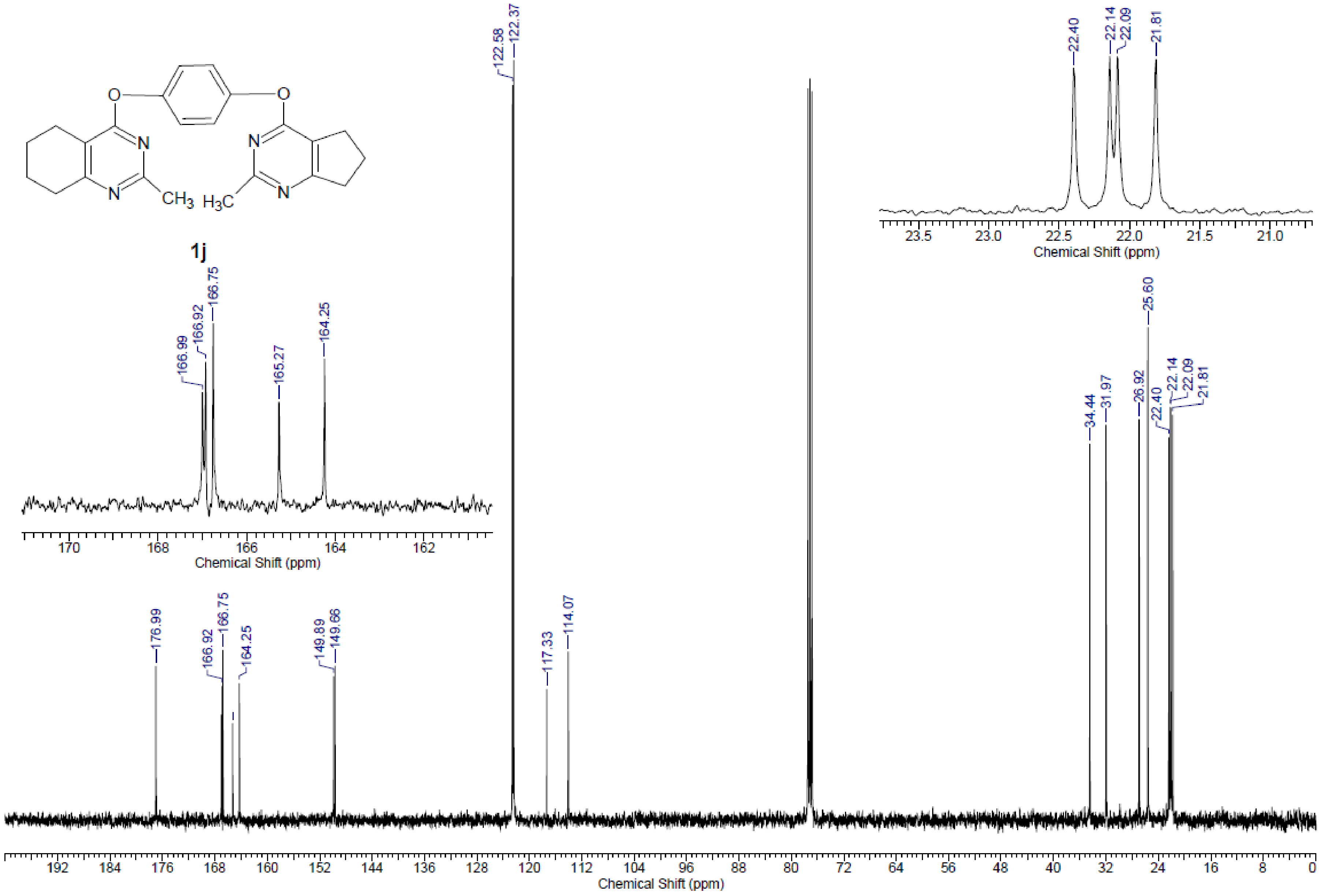

HSQC NMR spectrum ($CDCl_3$) of compound **1j**

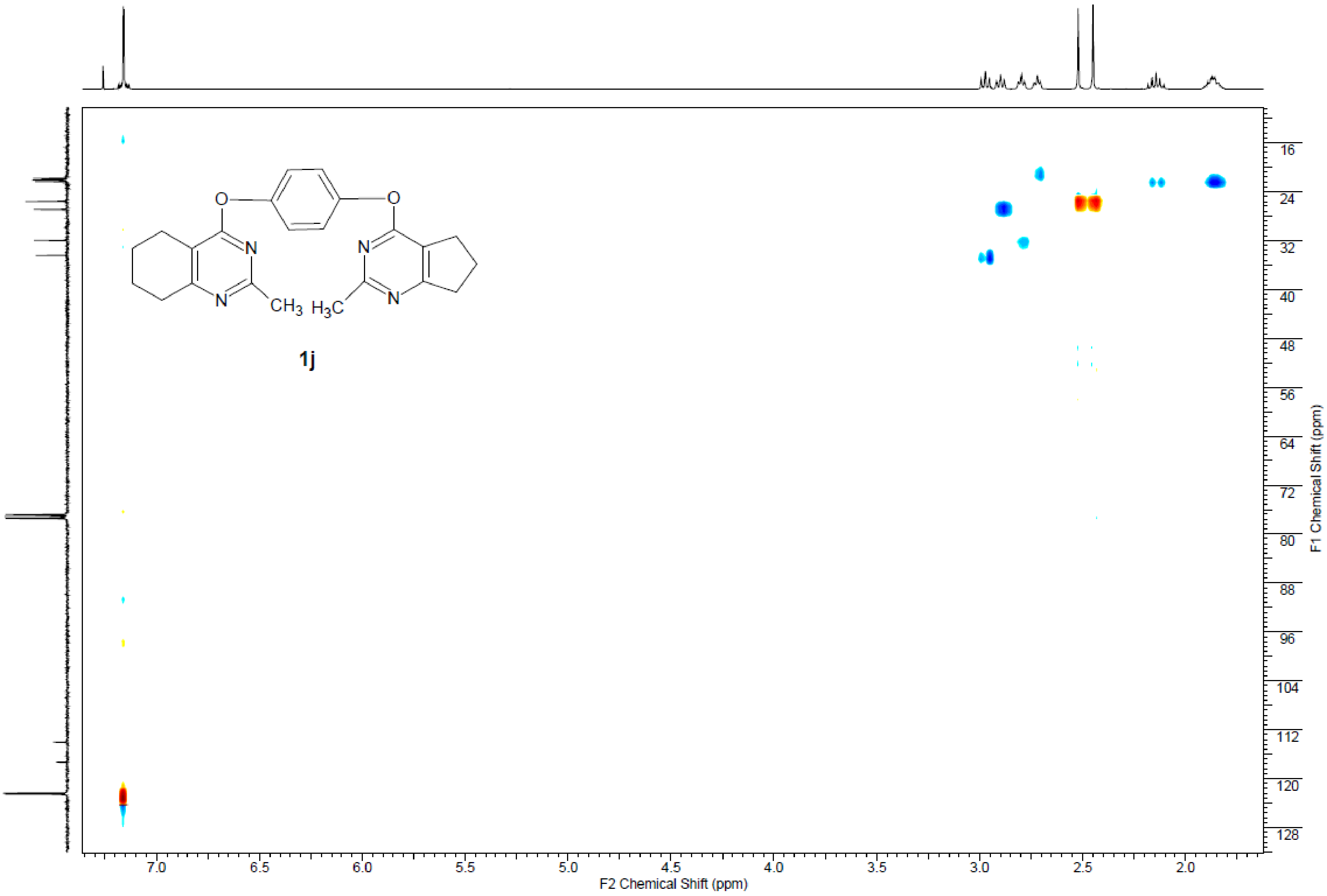

HMBC NMR spectrum ($CDCl_3$) of compound **1j**

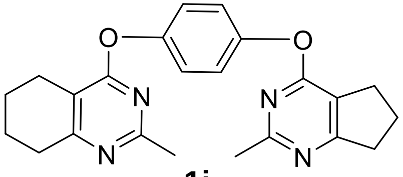

1j

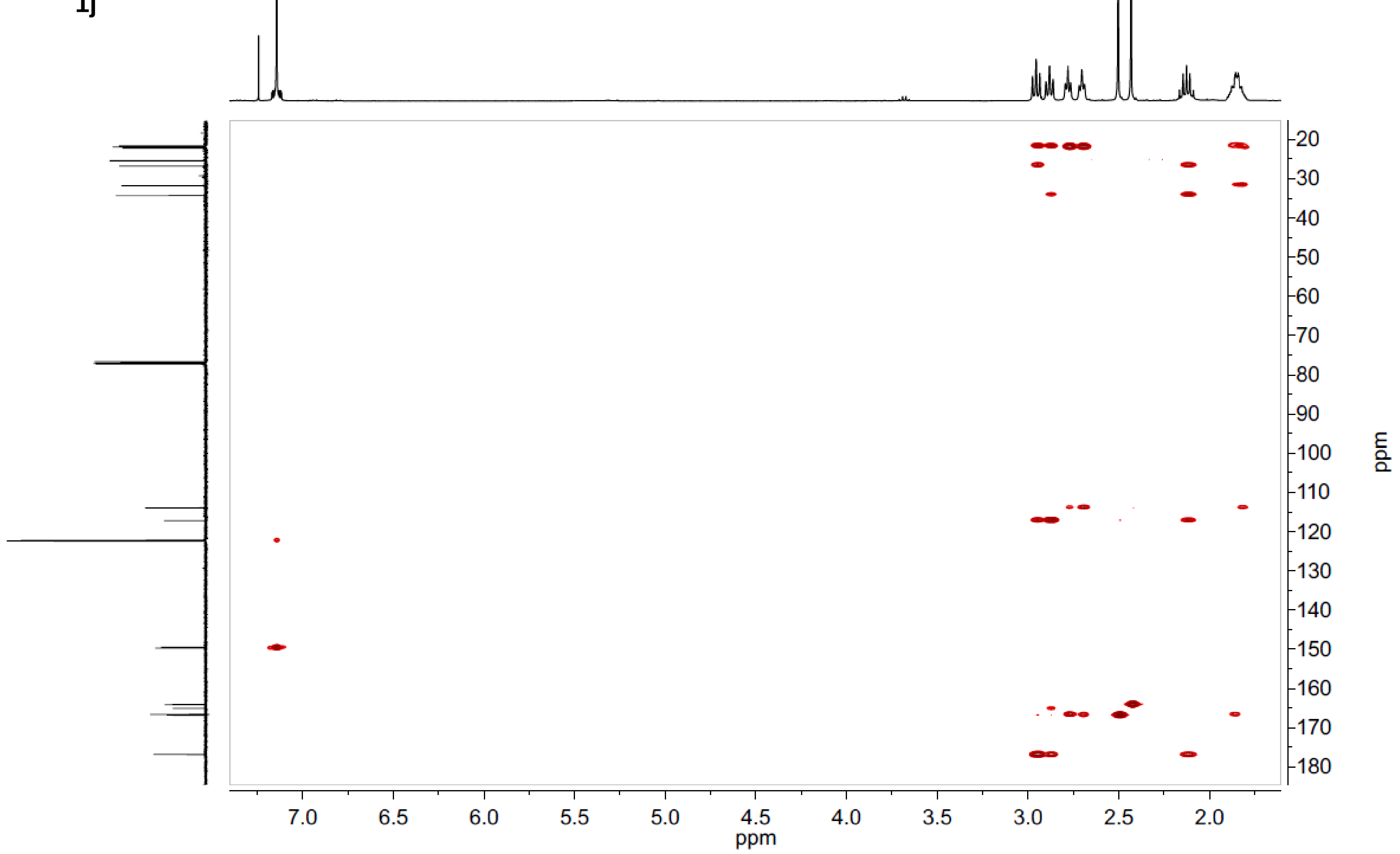

^{1}H NMR spectrum ($CDCl_3$) of compound **1k**

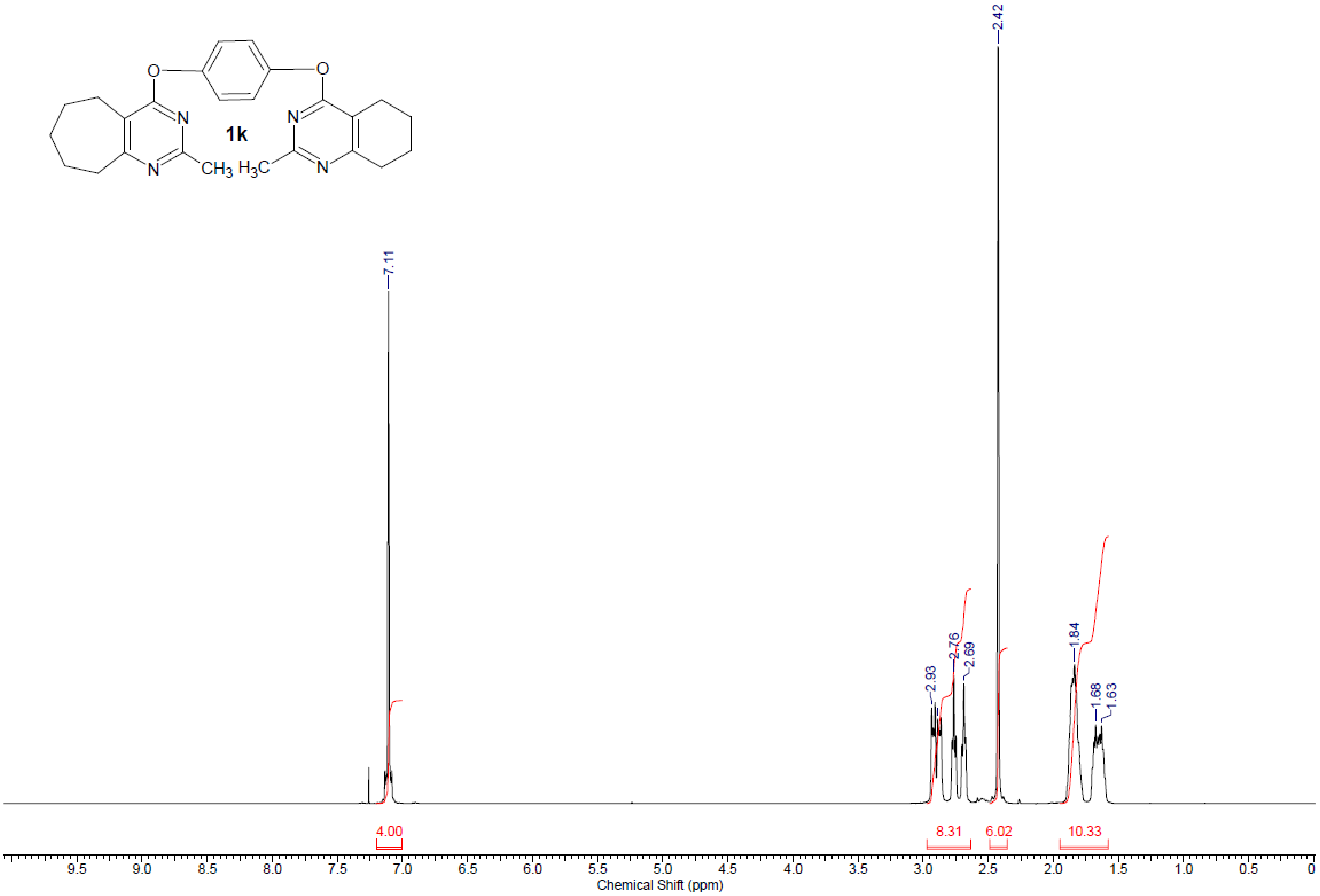

^{13}C NMR spectrum ($CDCl_3$) of compound **1k**

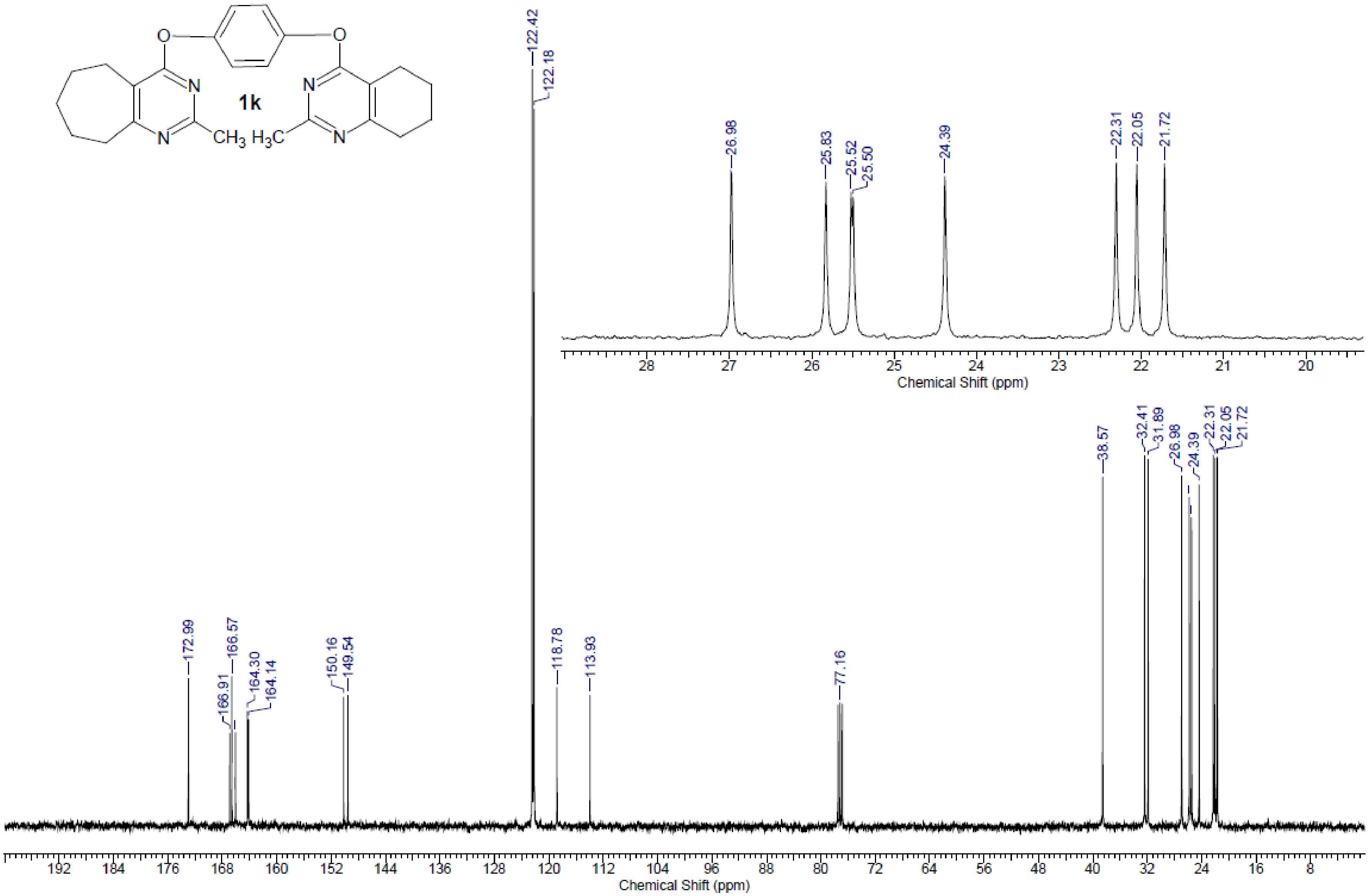

HSQC NMR spectrum ($CDCl_3$) of compound **1k**

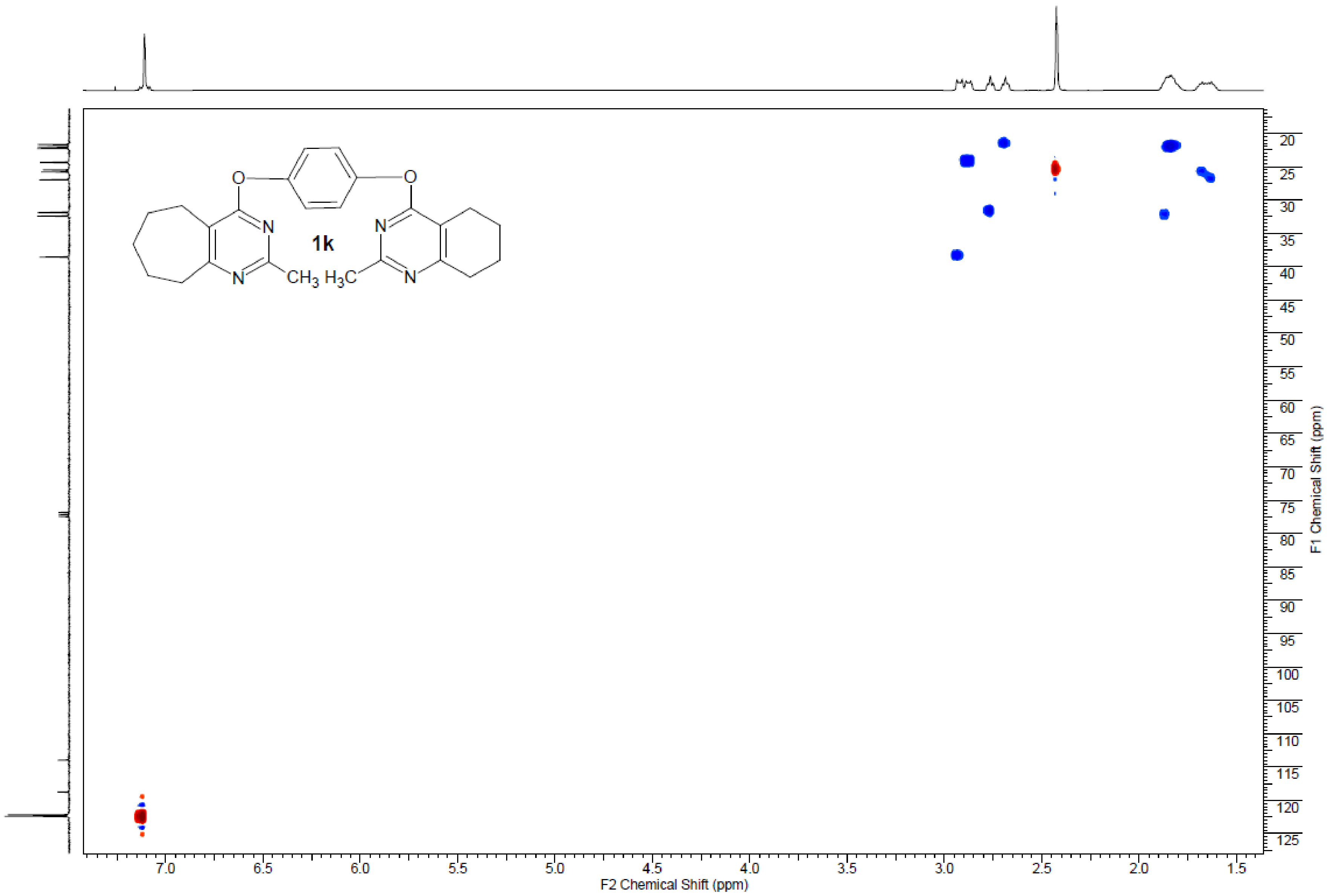

HMBC NMR spectrum ($CDCl_3$) of compound **1k**

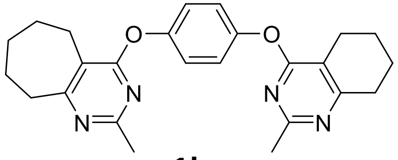

1k

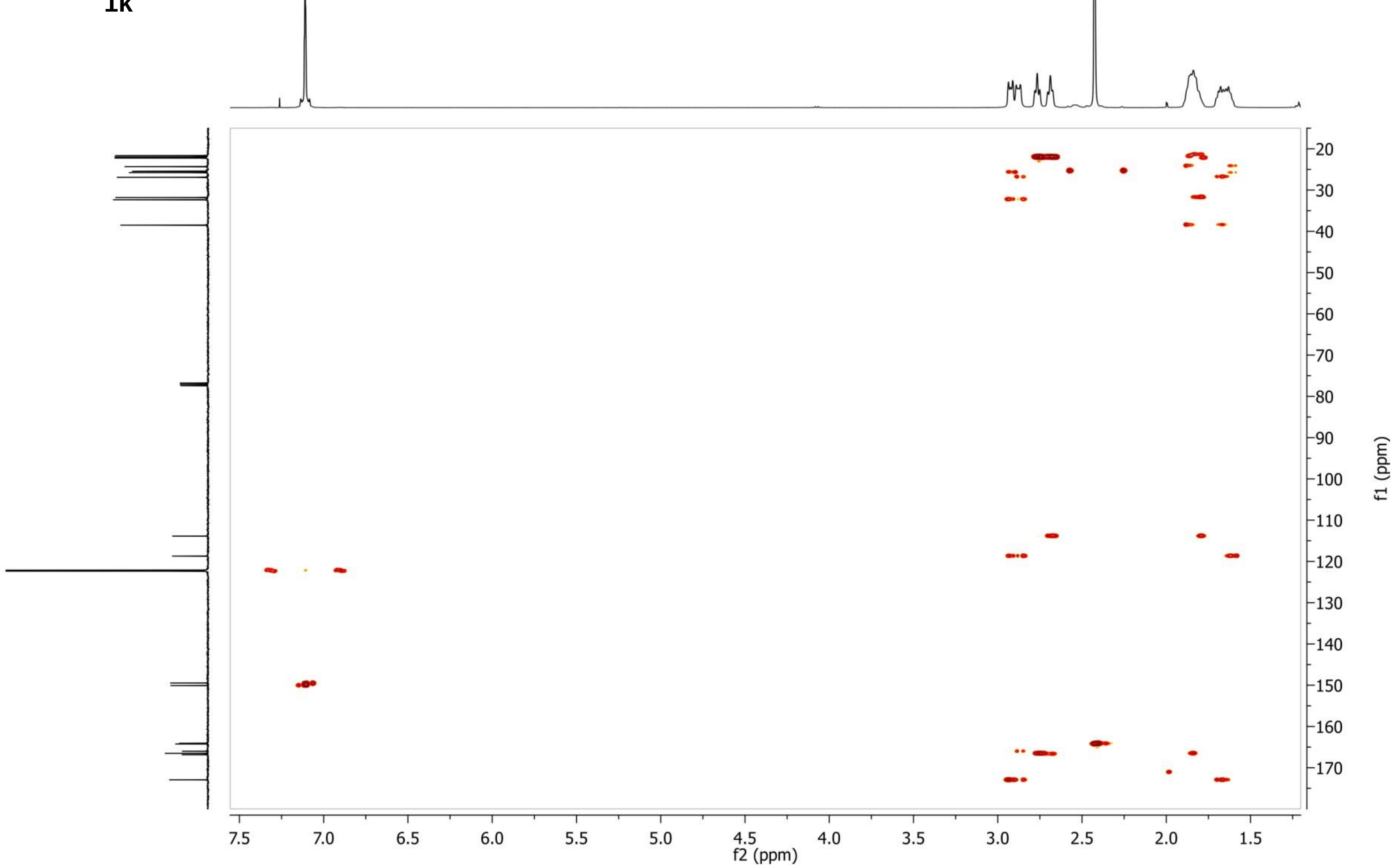

^{1}H NMR spectrum ($CDCl_3$) of compound **1l**

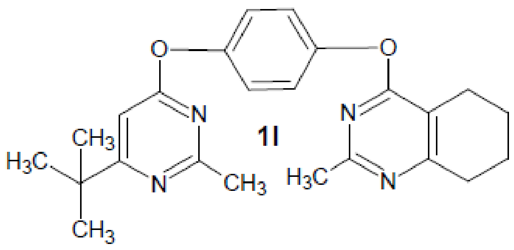

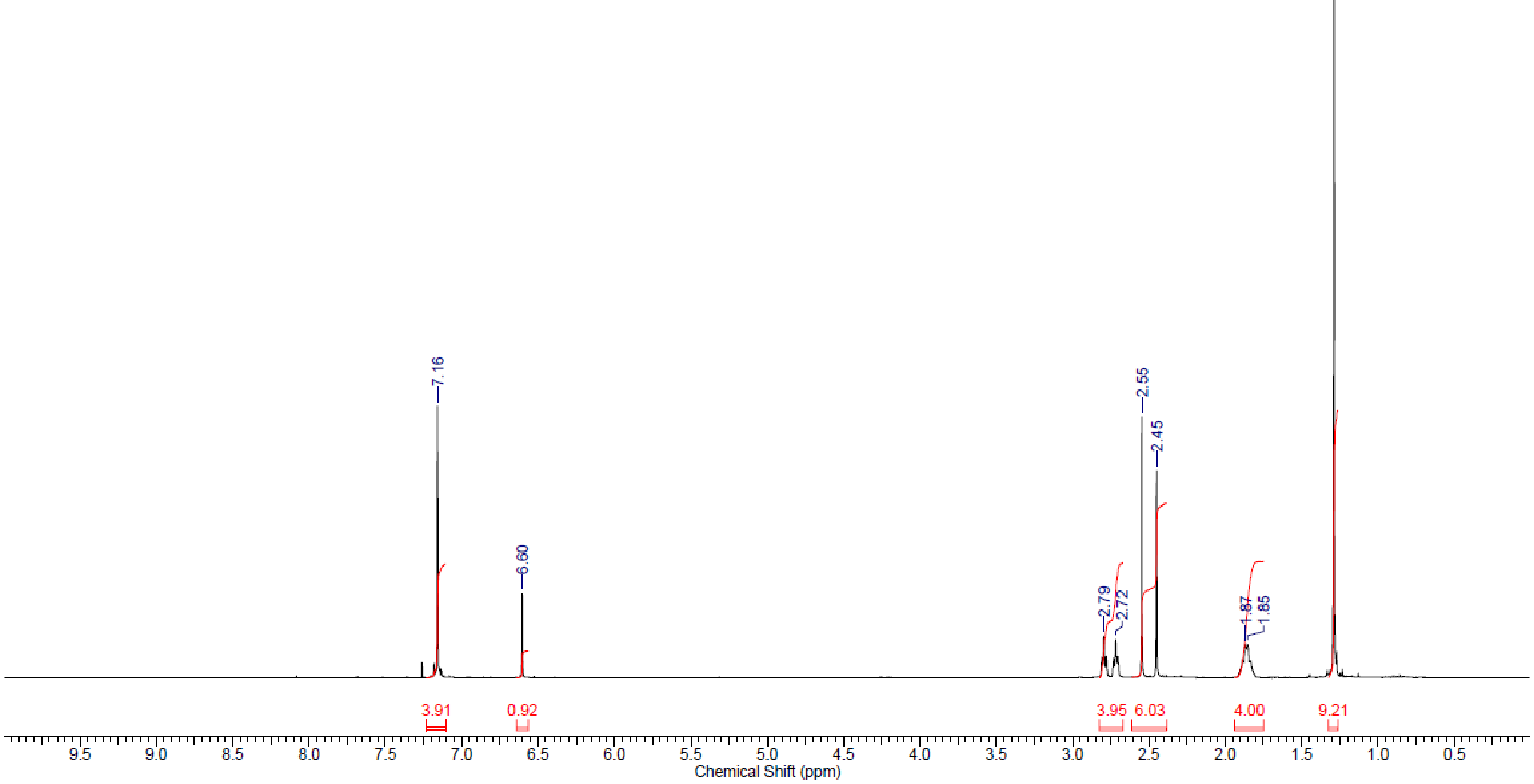

^{13}C NMR spectrum ($CDCl_3$) of compound **1l**

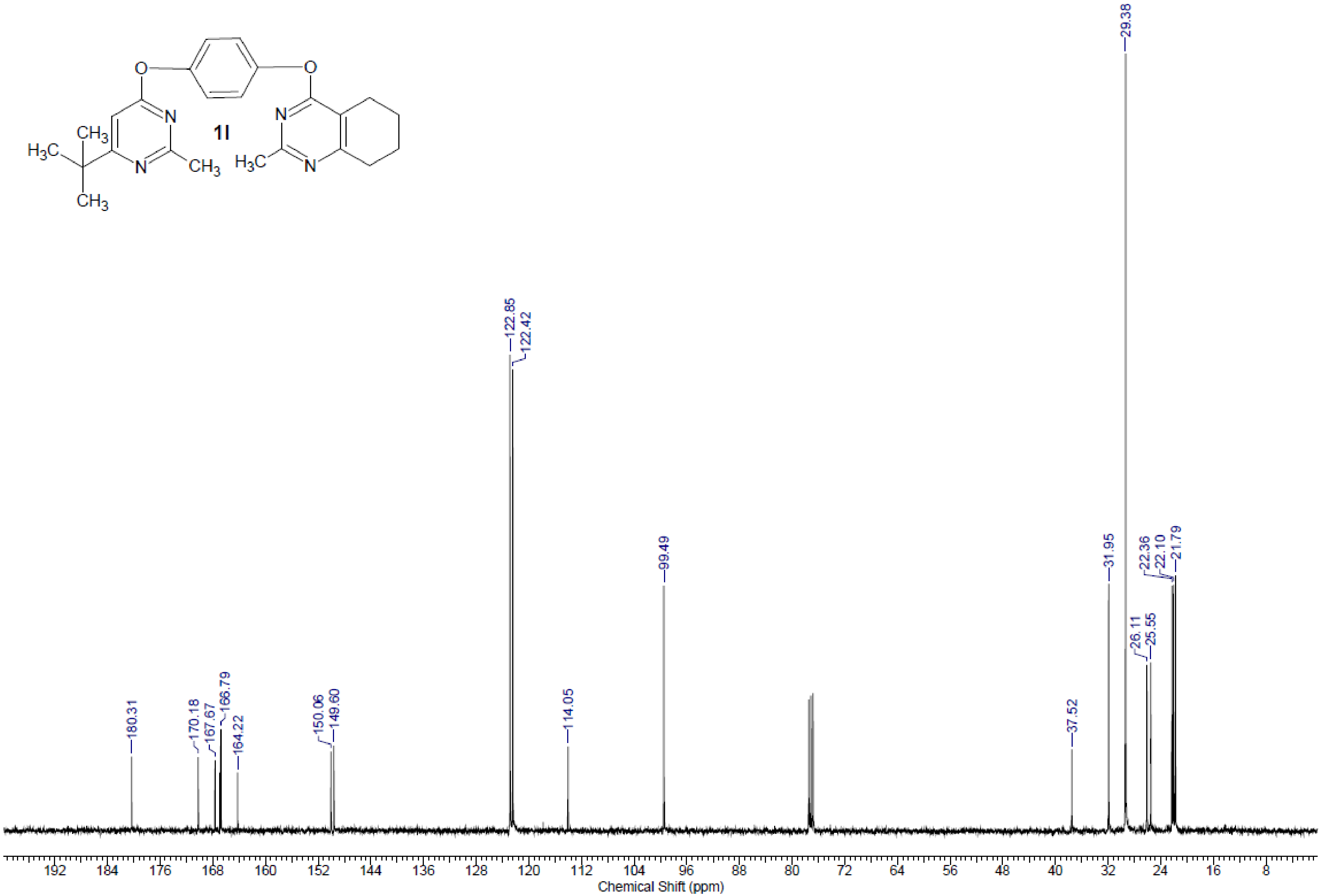

HSQC NMR spectrum ($CDCl_3$) of compound **1l**

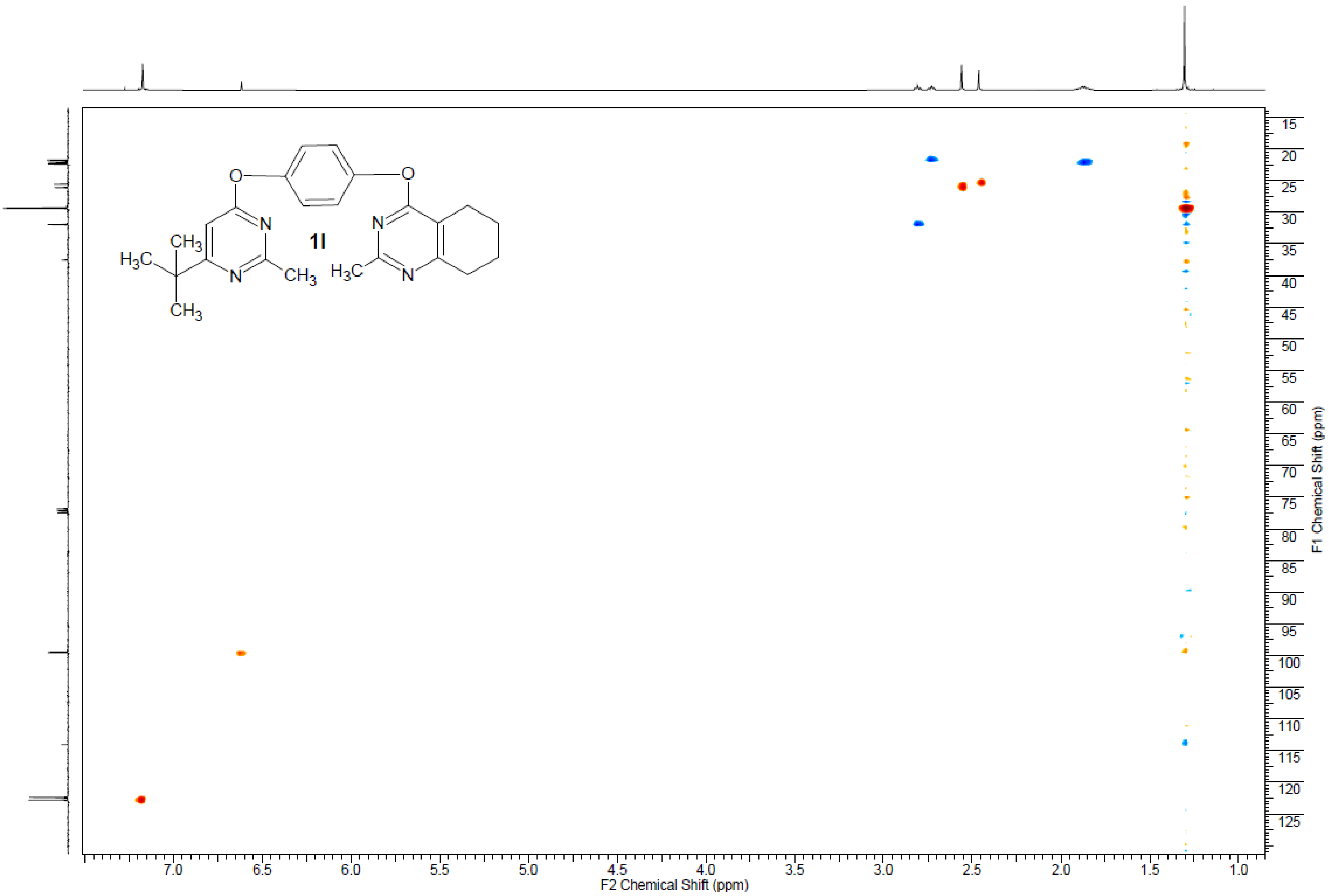

HMBC NMR spectrum ($CDCl_3$) of compound **1l**

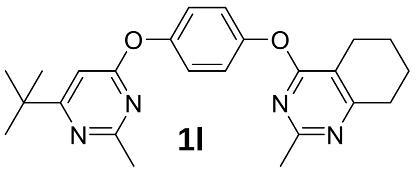

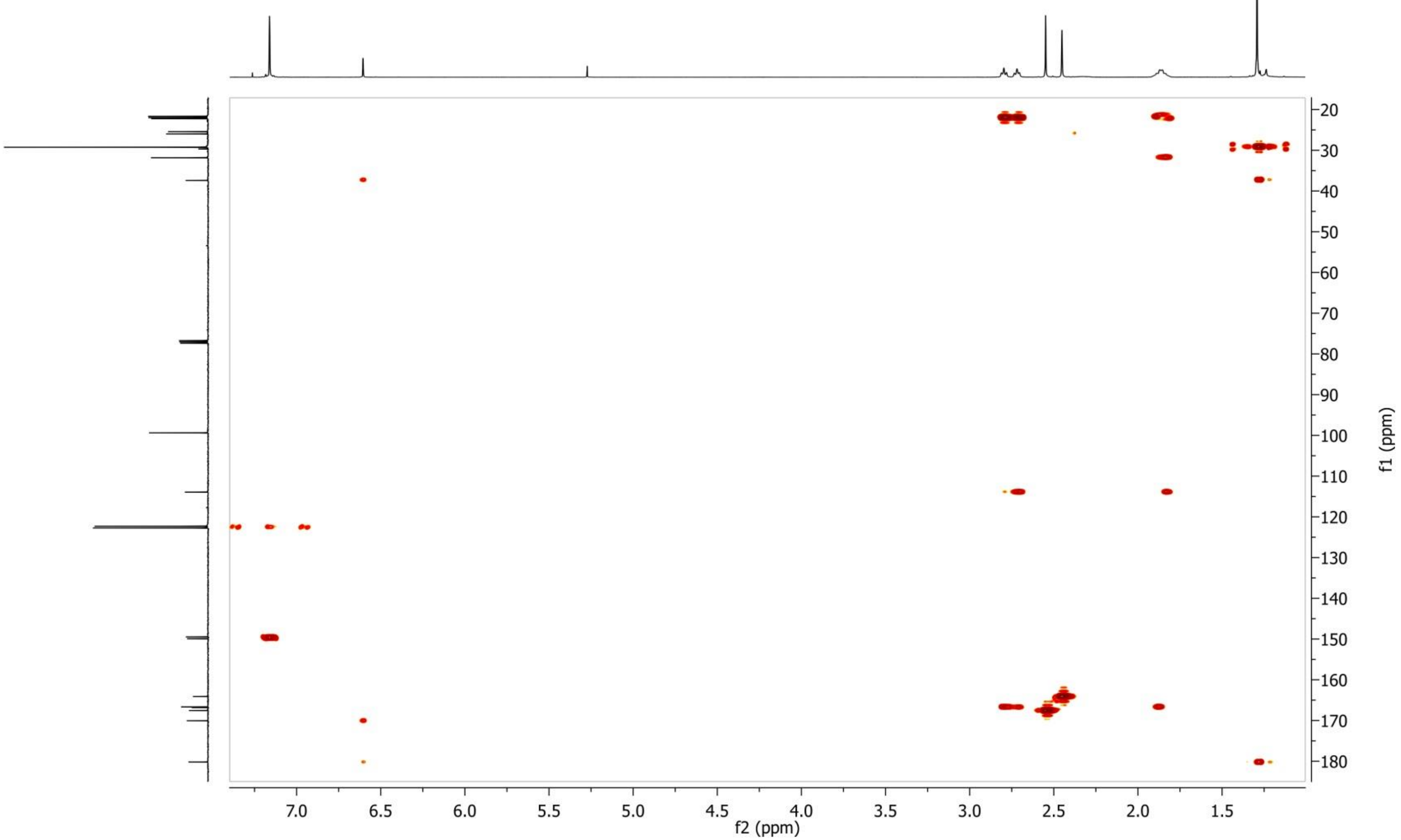

^{1}H NMR spectrum ($CDCl_3$) of compound **1m**

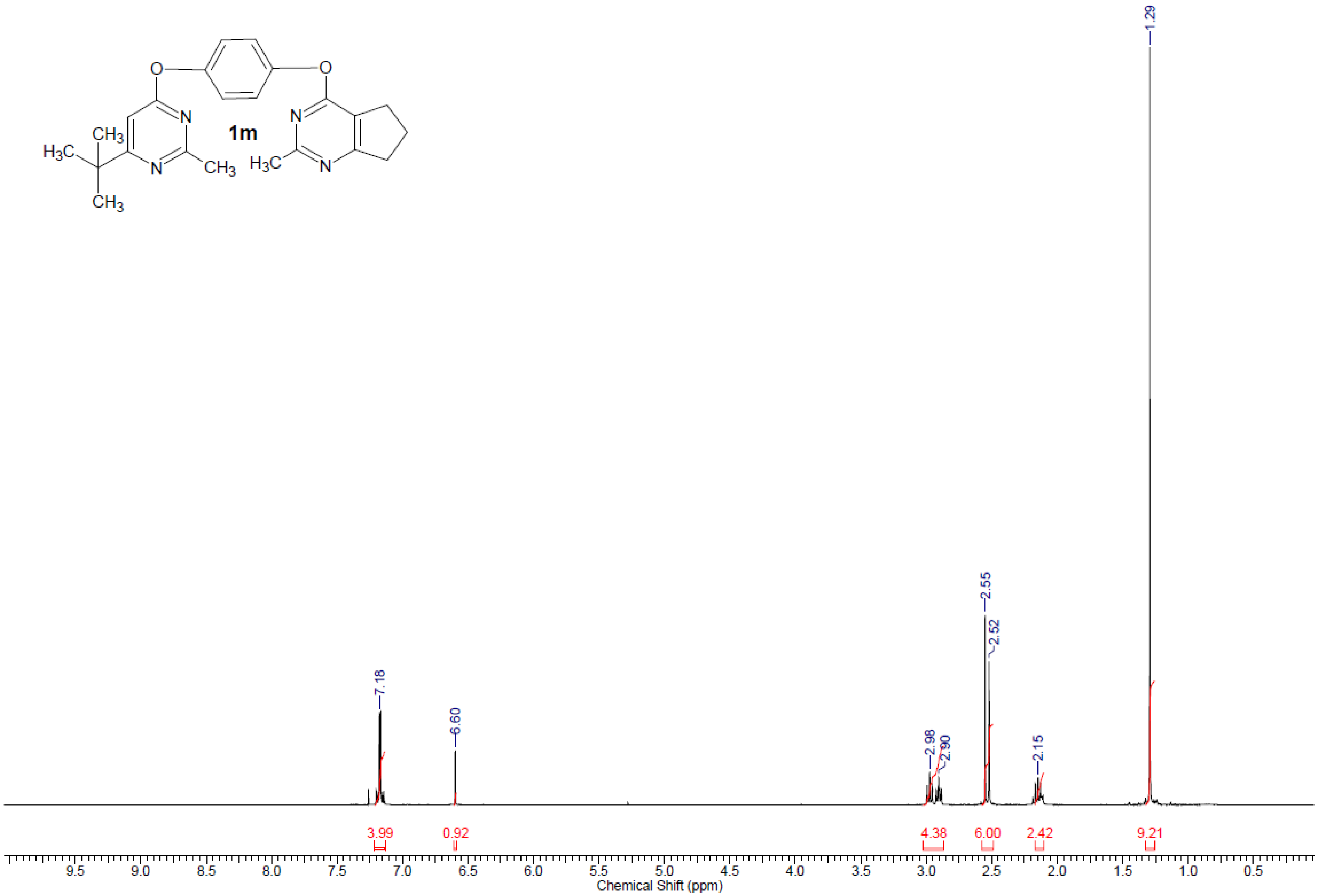

^{13}C NMR spectrum ($CDCl_3$) of compound **1m**

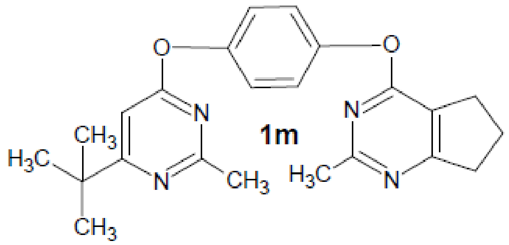

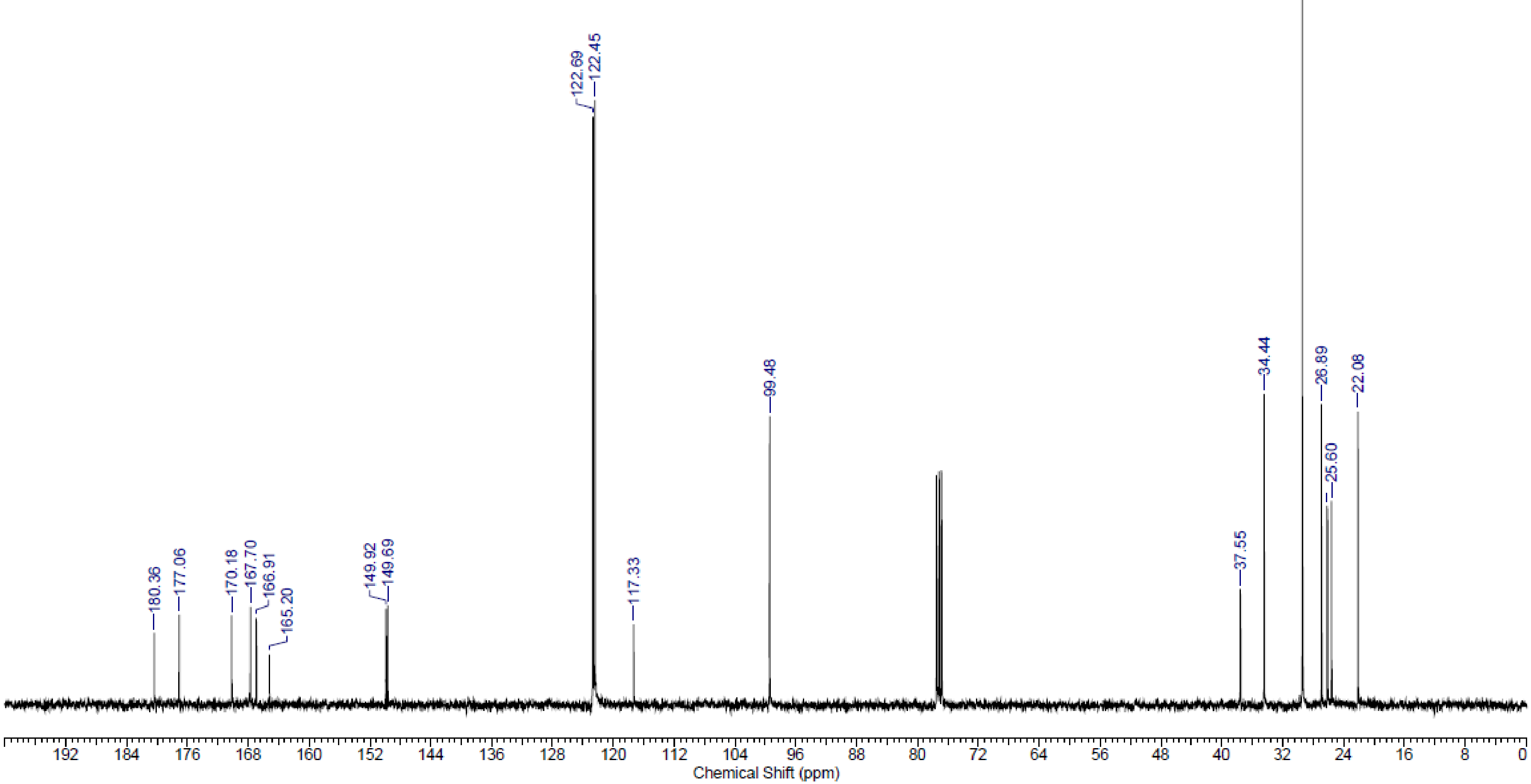

HSQC NMR spectrum ($CDCl_3$) of compound **1m**

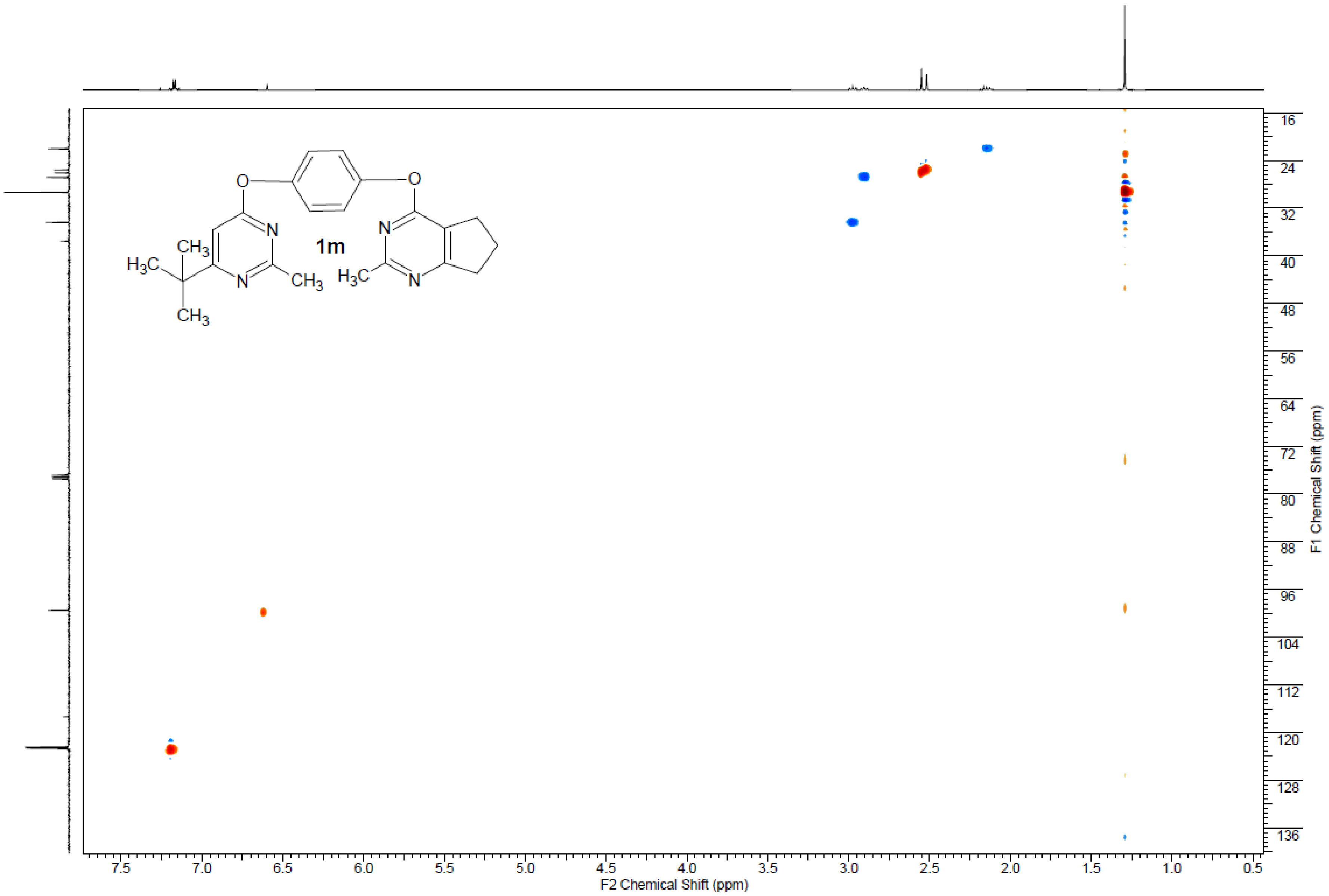

HMBC NMR spectrum ($CDCl_3$) of compound **1m**

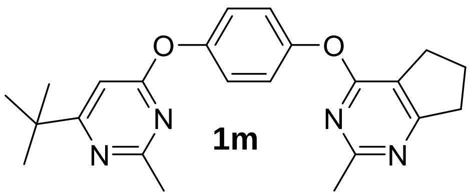

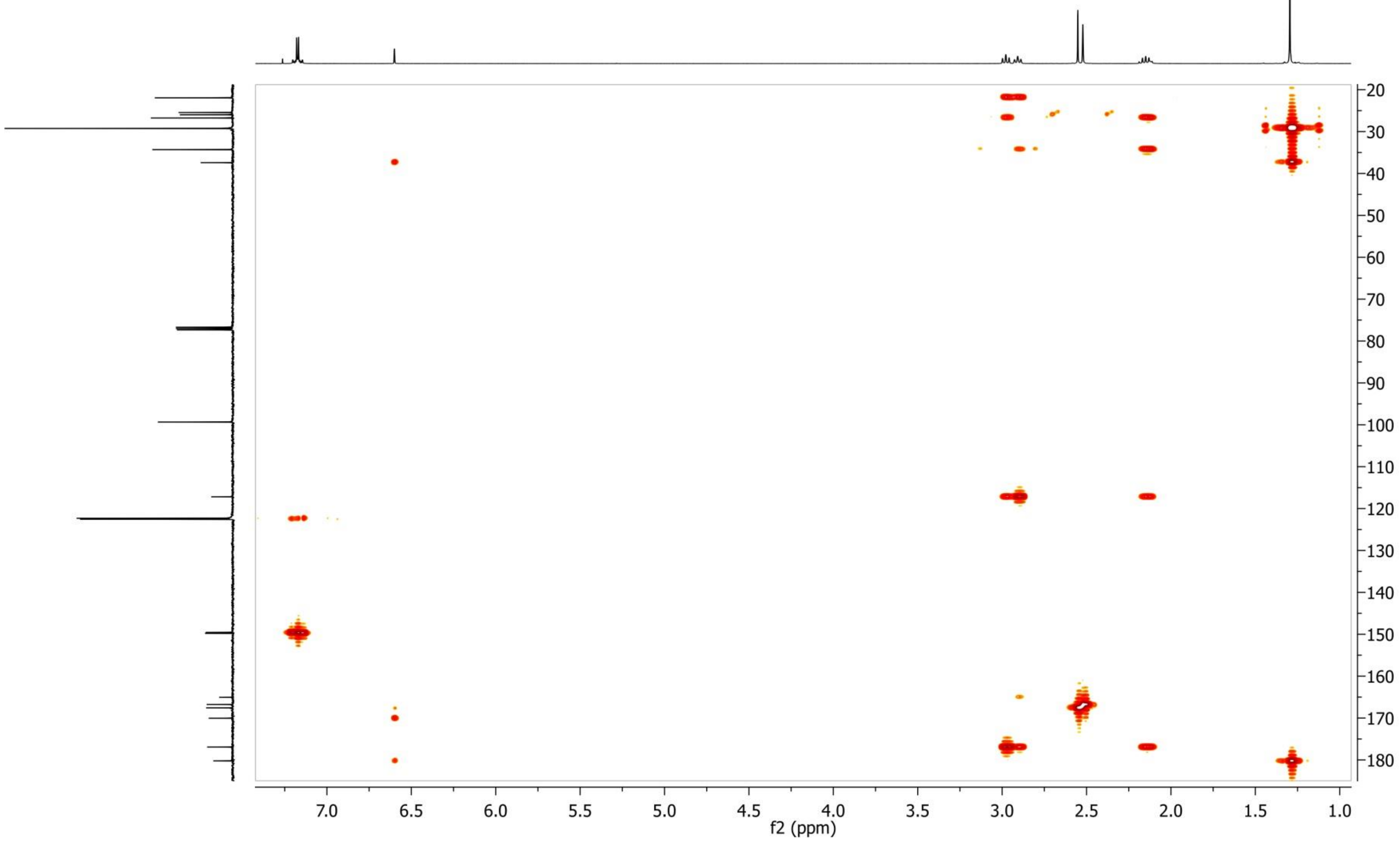

^{1}H NMR spectrum ($CDCl_3$) of compound **1n**

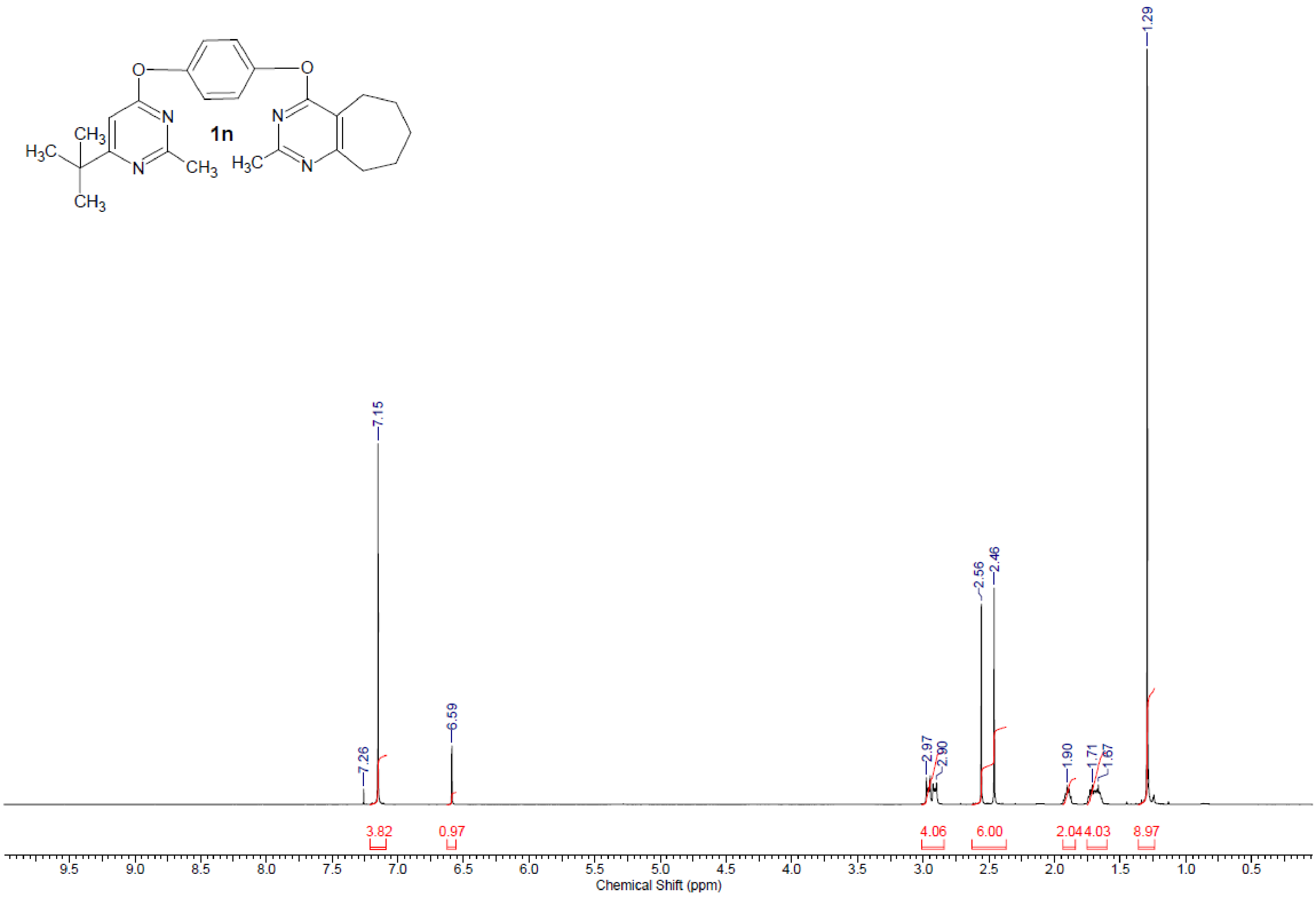

^{13}C NMR spectrum ($CDCl_3$) of compound **1n**

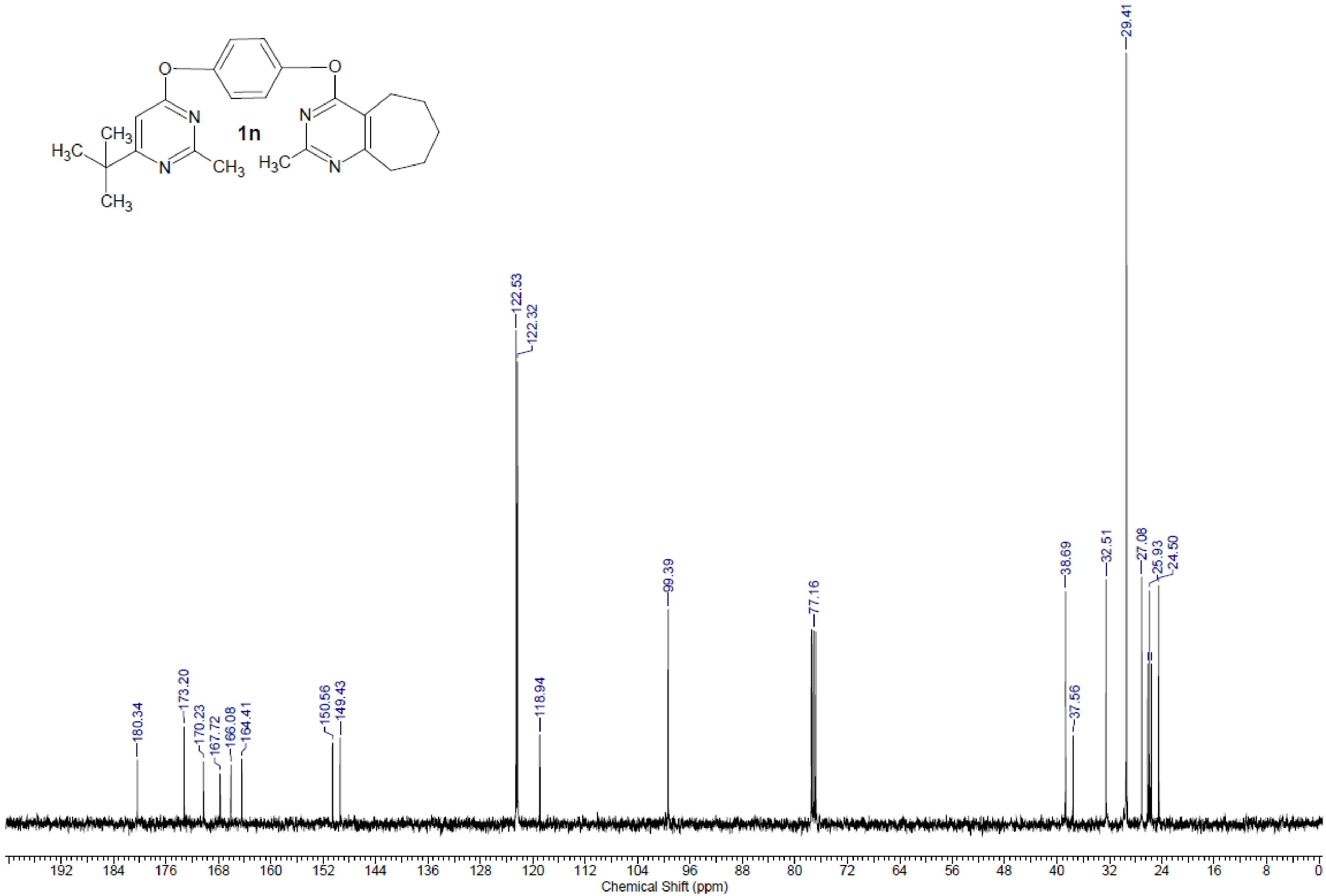

HSQC NMR spectrum ($CDCl_3$) of compound **1n**

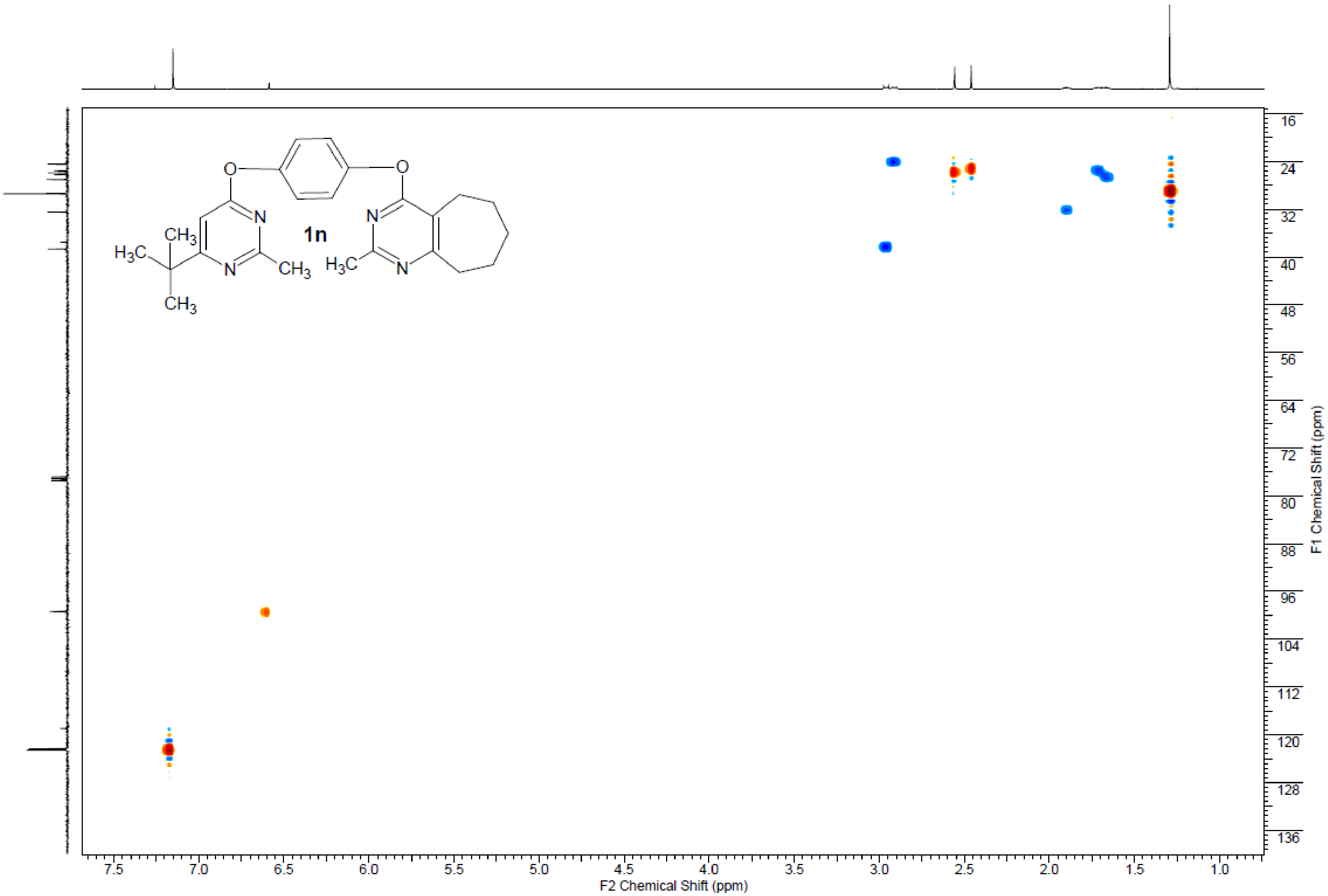

HMBC NMR spectrum ($CDCl_3$) of compound **1n**

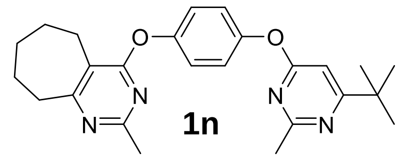

1n

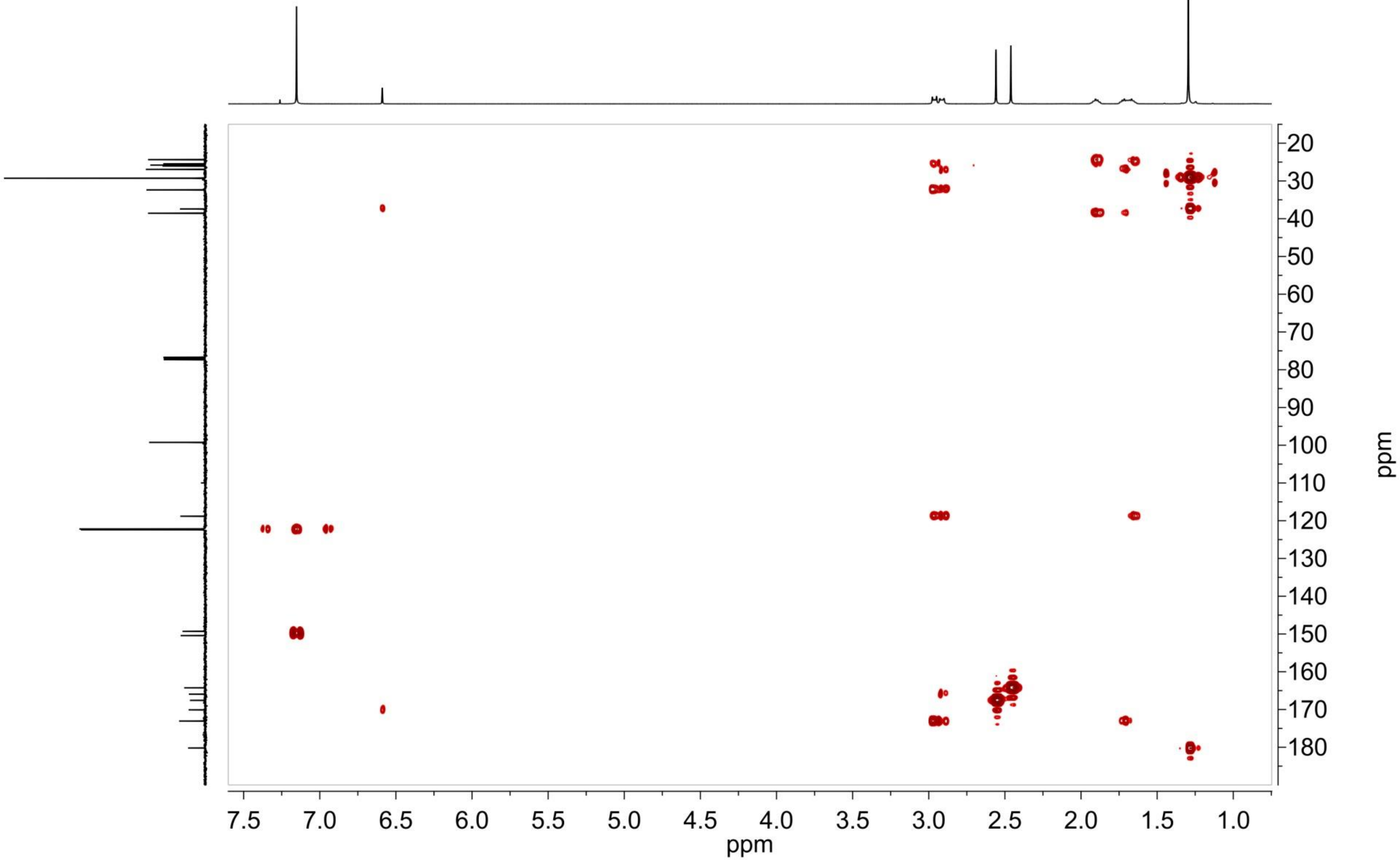

^{1}H NMR spectrum ($CDCl_3$) of compound **1o**

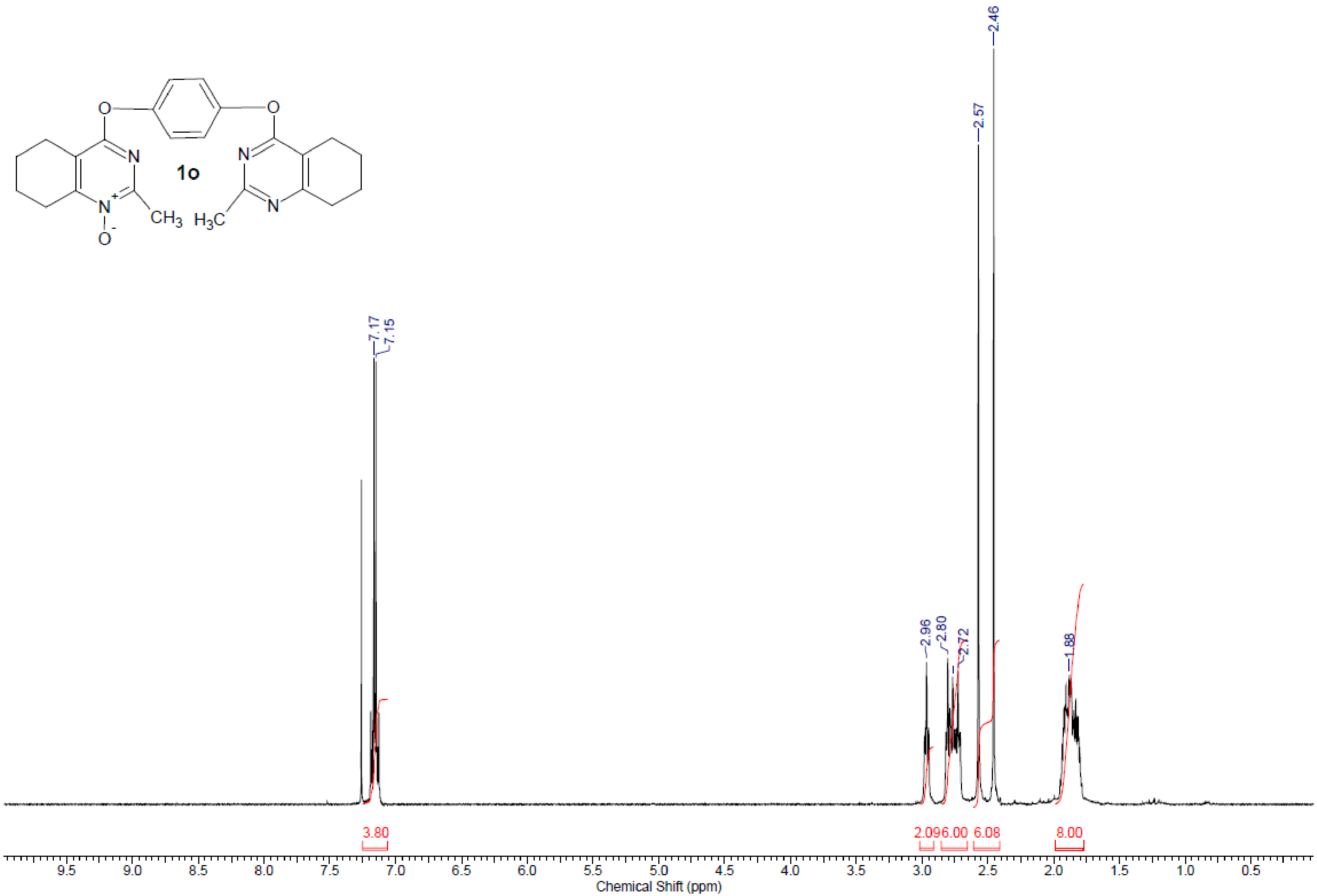

^{13}C NMR spectrum ($CDCl_3$) of compound **1o**

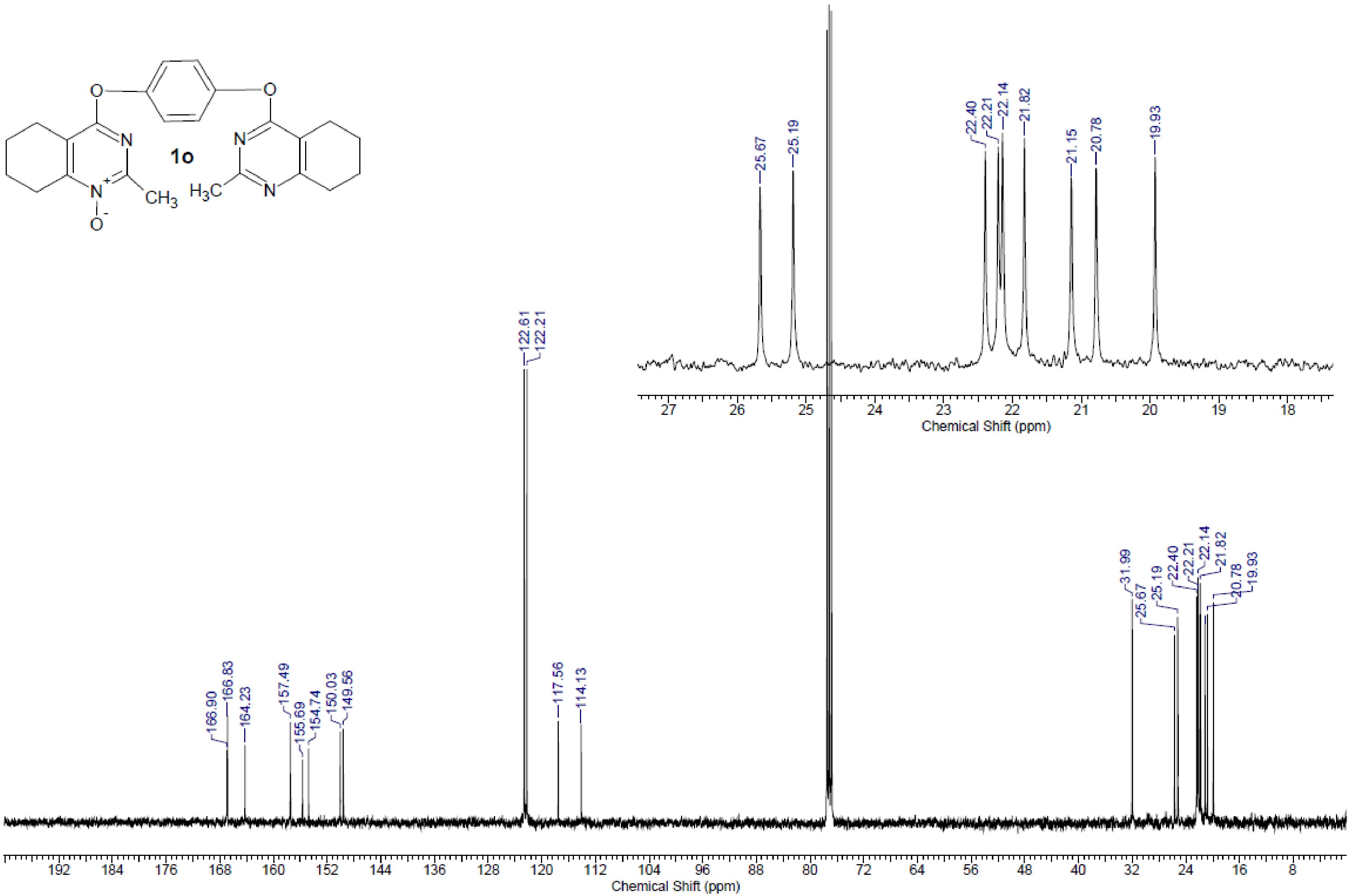

HSQC NMR spectrum ($CDCl_3$) of compound **1o**

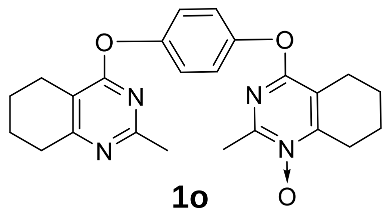

1o

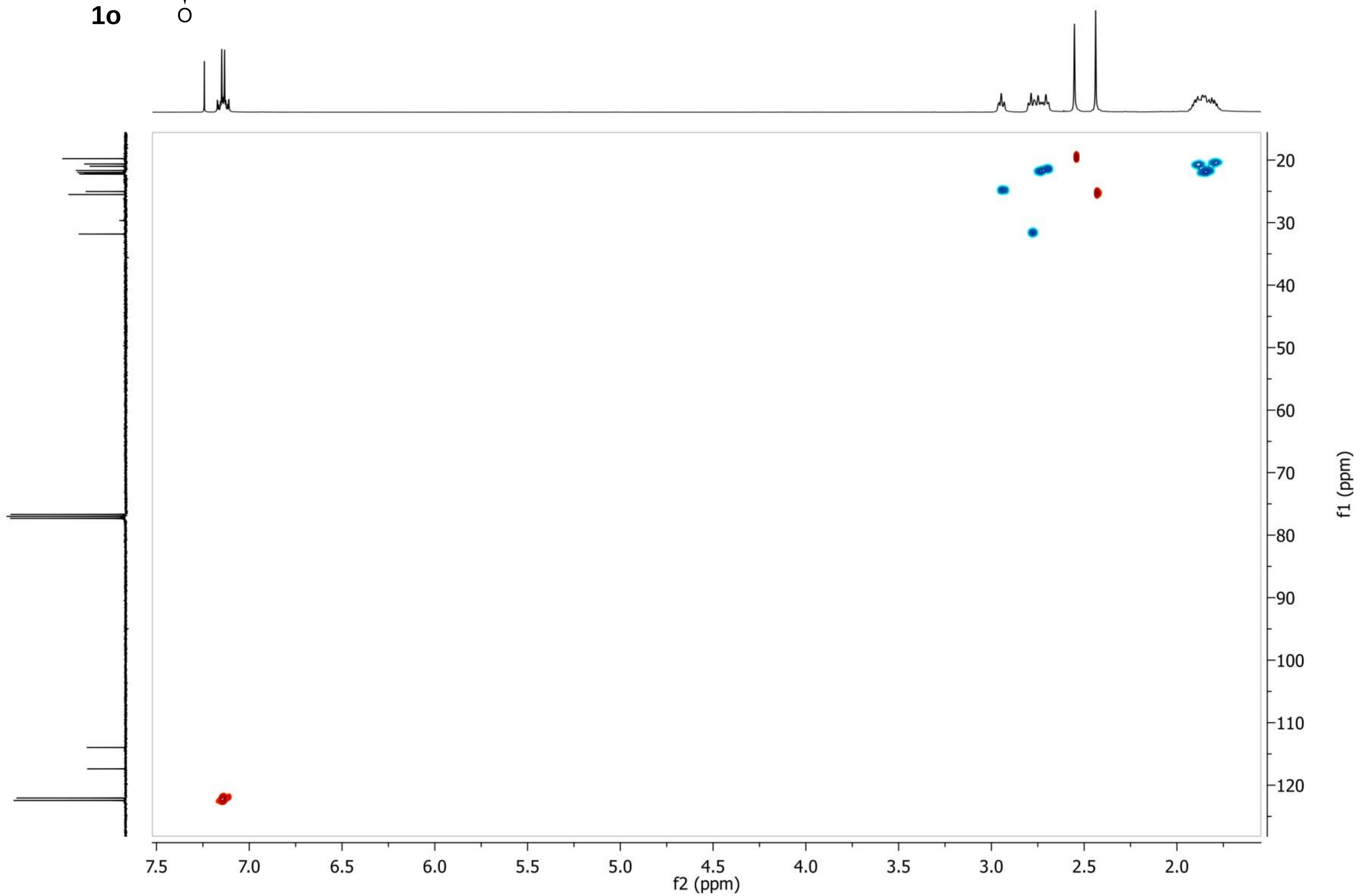

HMBC NMR spectrum ($CDCl_3$) of compound **1o**

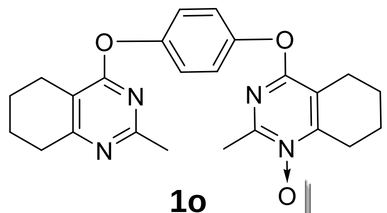

1o

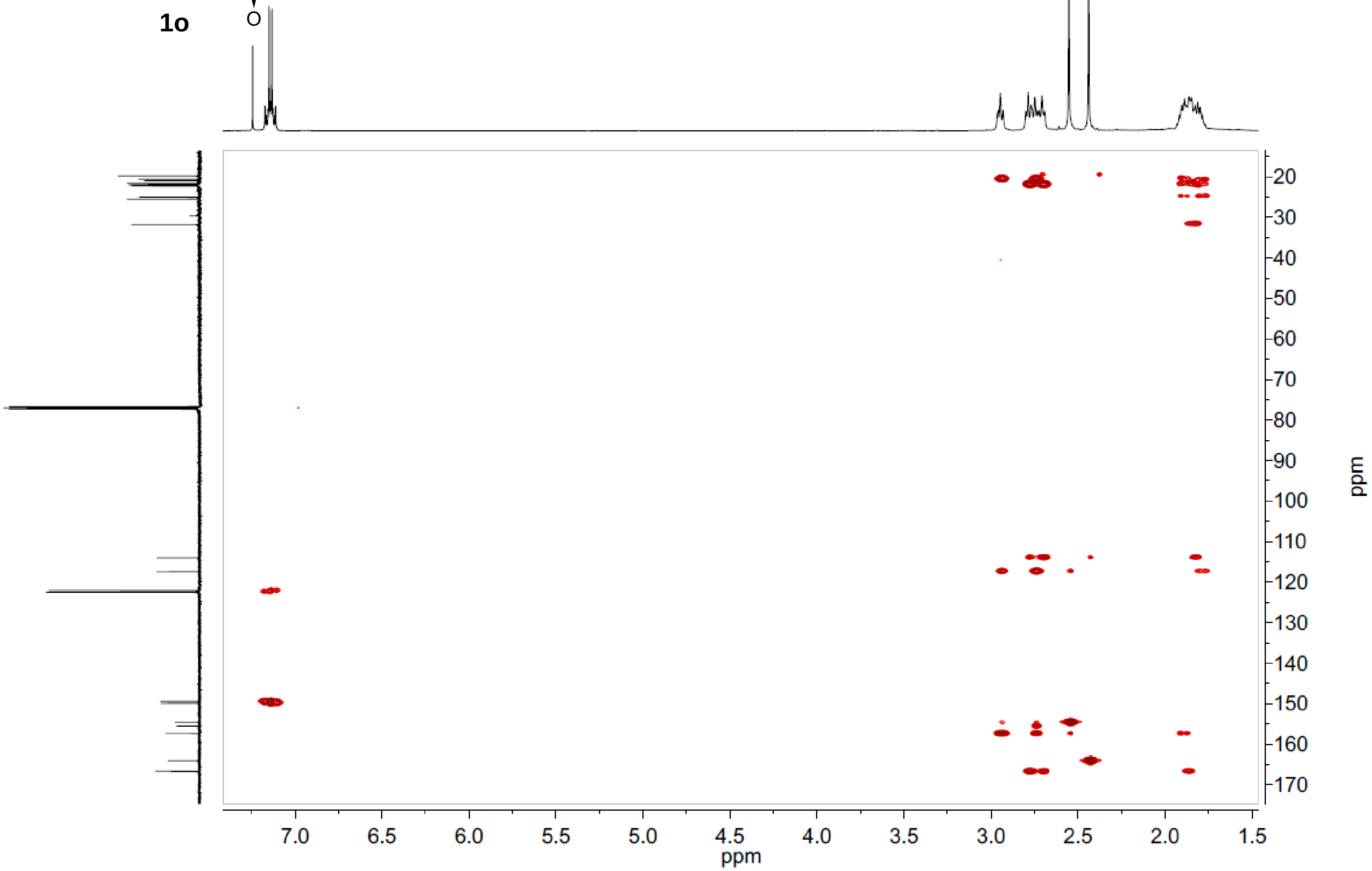

^{1}H NMR spectrum ($CDCl_3$) of compound **1p**

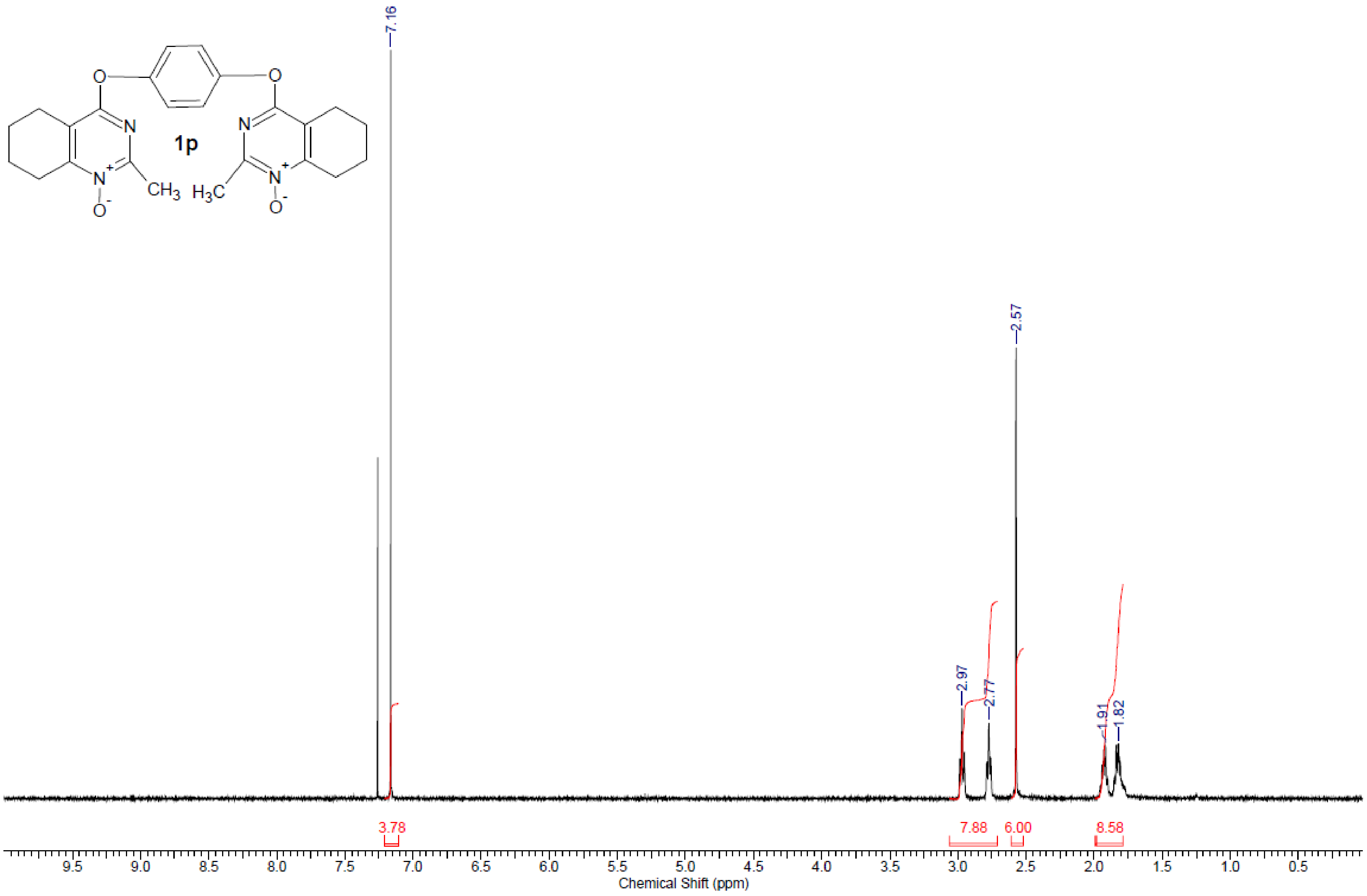

3. Copies of HRMS spectra

HRMS spectrum of compound **1f**

Acquisition Parameter

Source Type	ESI	Ion Polarity	Positive	Set Nebulizer	0.4 Bar
Focus	Not active			Set Dry Heater	180 °C
Scan Begin	50 m/z	Set Capillary	4500 V	Set Dry Gas	4.0 l/min
Scan End	3000 m/z	Set End Plate Offset	-500 V	Set Divert Valve	Waste

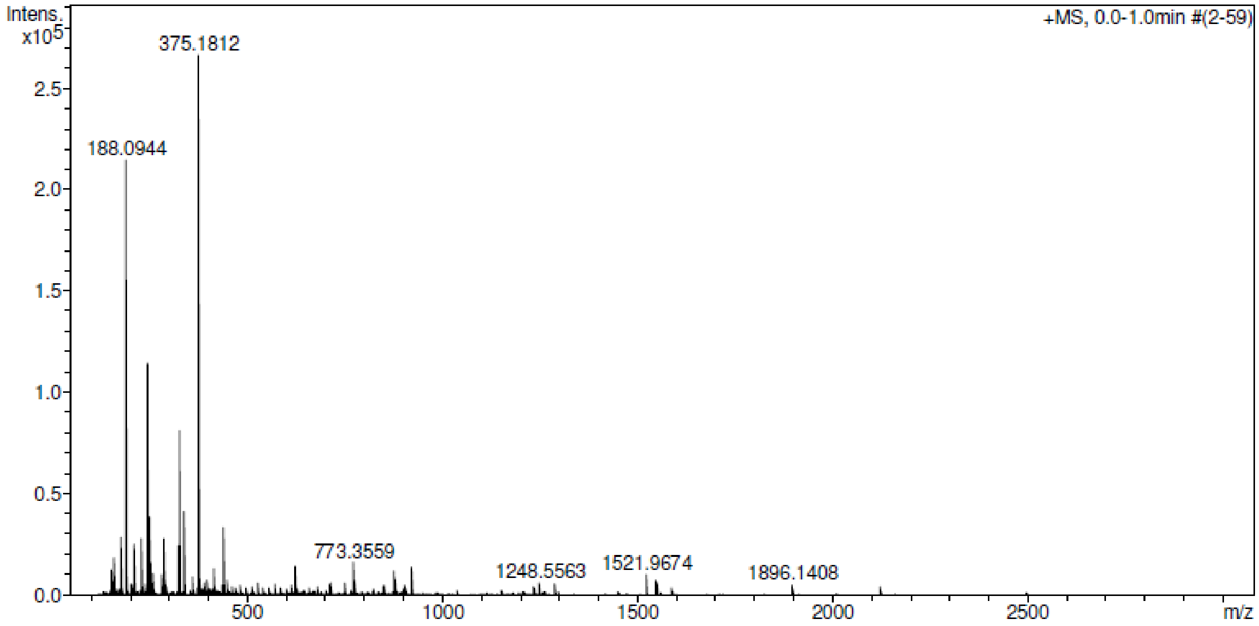

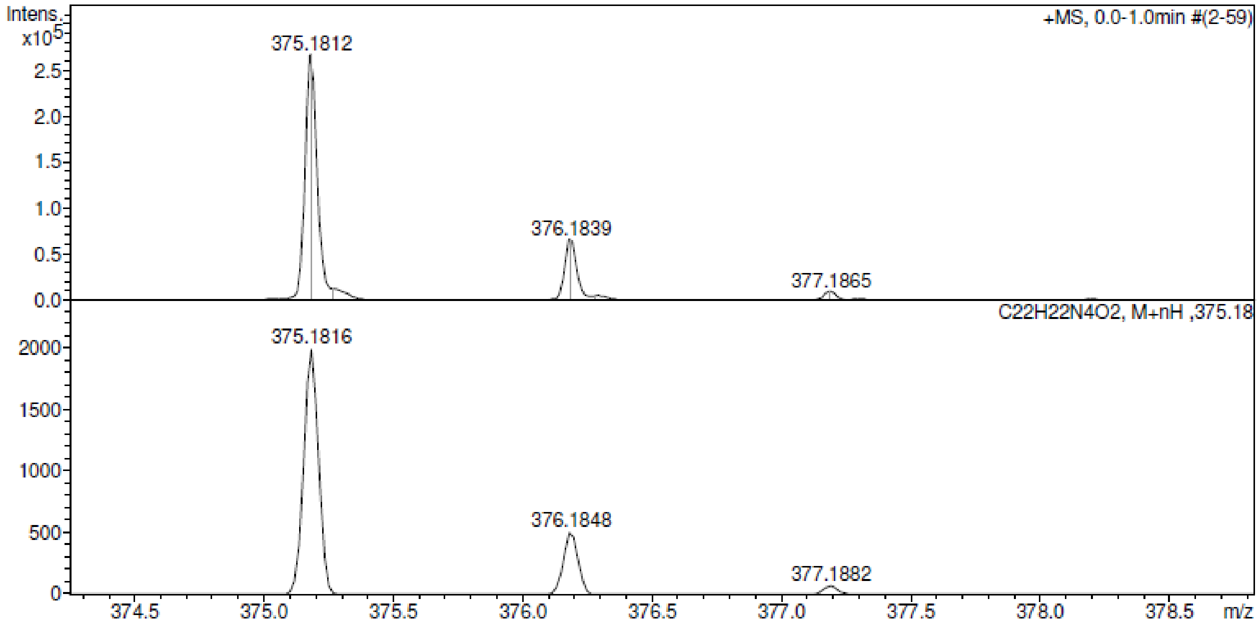

HRMS spectrum of compound **1g**

Acquisition Parameter

Source Type	ESI	Ion Polarity	Positive	Set Nebulizer	0.4 Bar
Focus	Not active			Set Dry Heater	180 °C
Scan Begin	50 m/z	Set Capillary	4500 V	Set Dry Gas	4.0 l/min
Scan End	3000 m/z	Set End Plate Offset	-500 V	Set Divert Valve	Waste

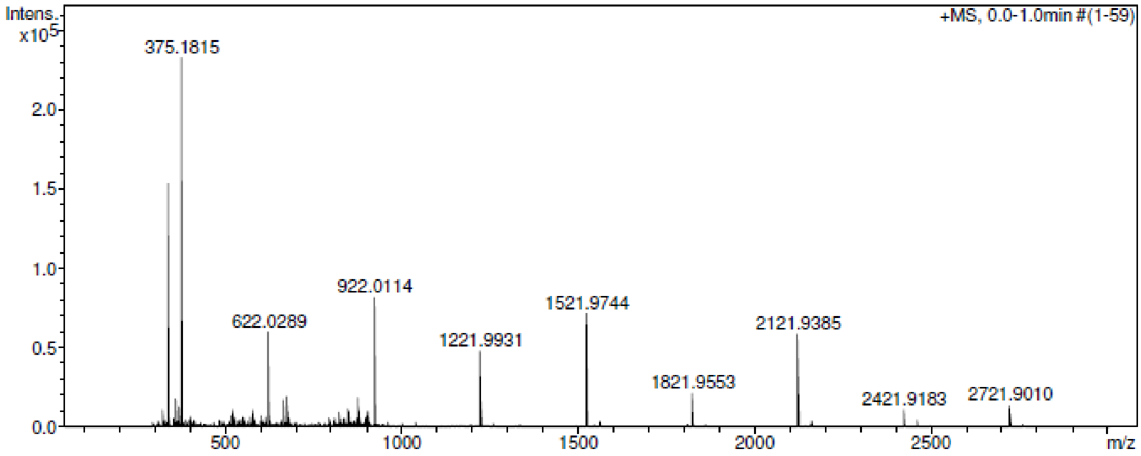

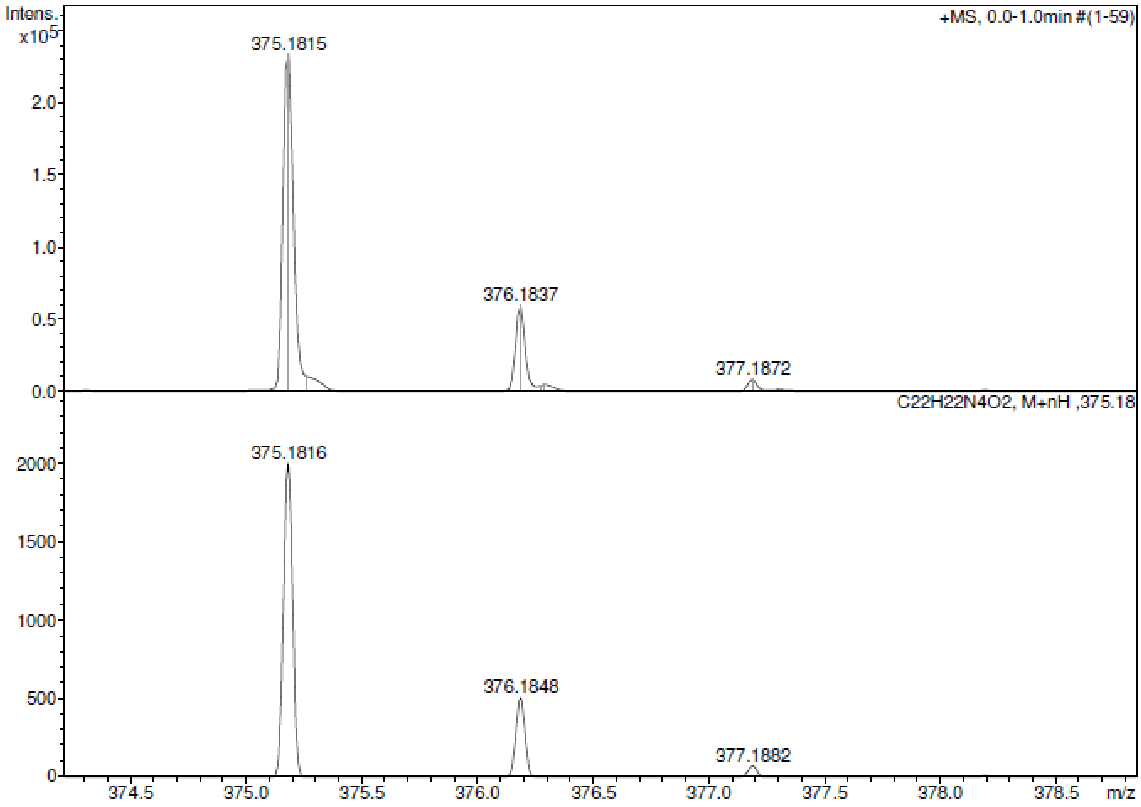

HRMS spectrum of compound **1h**

Acquisition Parameter

Source Type	ESI	Ion Polarity	Positive	Set Nebulizer	0.4 Bar
Focus	Not active			Set Dry Heater	180 °C
Scan Begin	50 m/z	Set Capillary	4500 V	Set Dry Gas	4.0 l/min
Scan End	3000 m/z	Set End Plate Offset	-500 V	Set Divert Valve	Waste

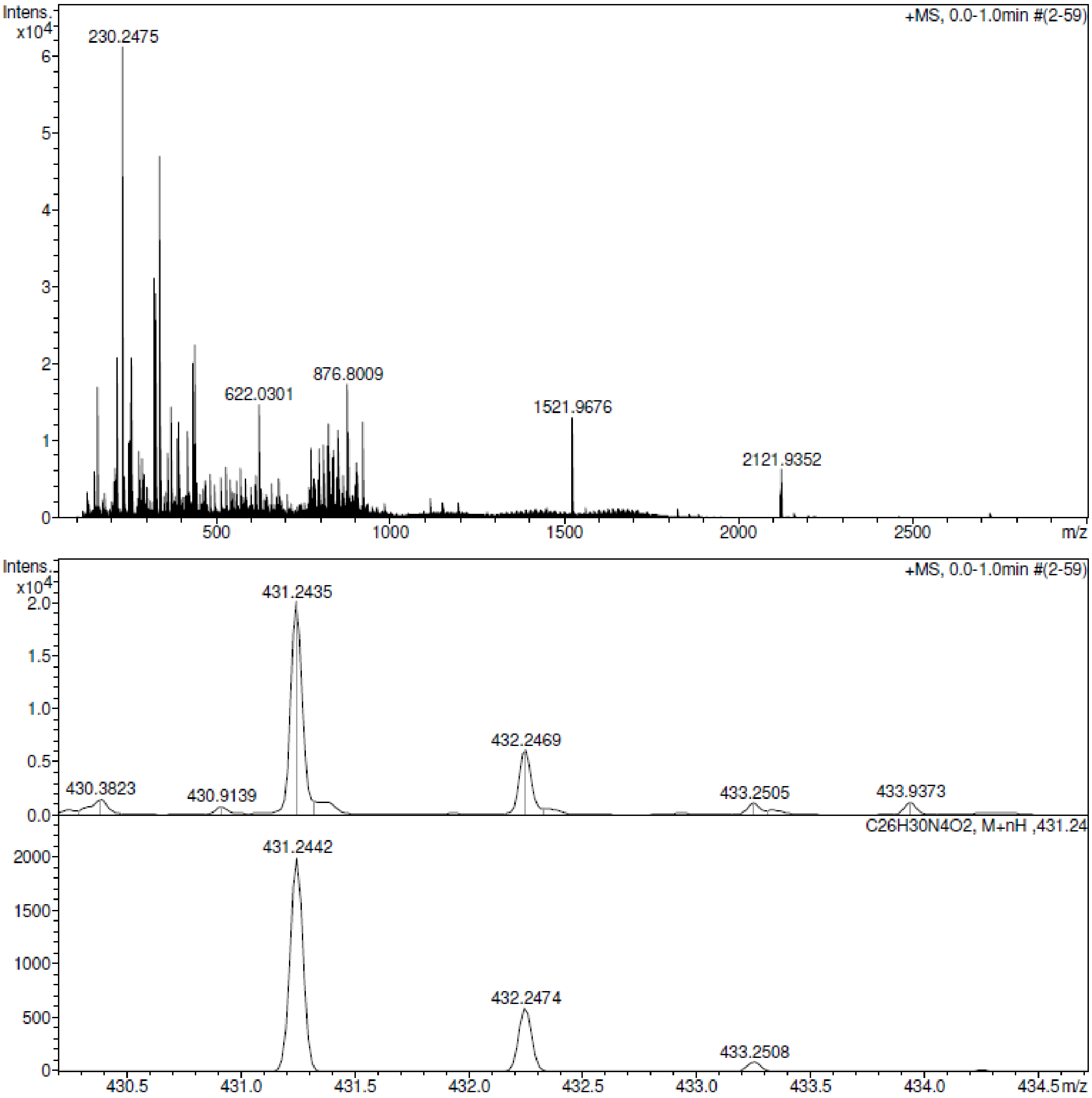

HRMS spectrum of compound **1i**

Acquisition Parameter

Source Type	ESI	Ion Polarity	Positive	Set Nebulizer	0.4 Bar
Focus	Not active			Set Dry Heater	180 °C
Scan Begin	50 m/z	Set Capillary	4500 V	Set Dry Gas	4.0 l/min
Scan End	3000 m/z	Set End Plate Offset	-500 V	Set Divert Valve	Waste

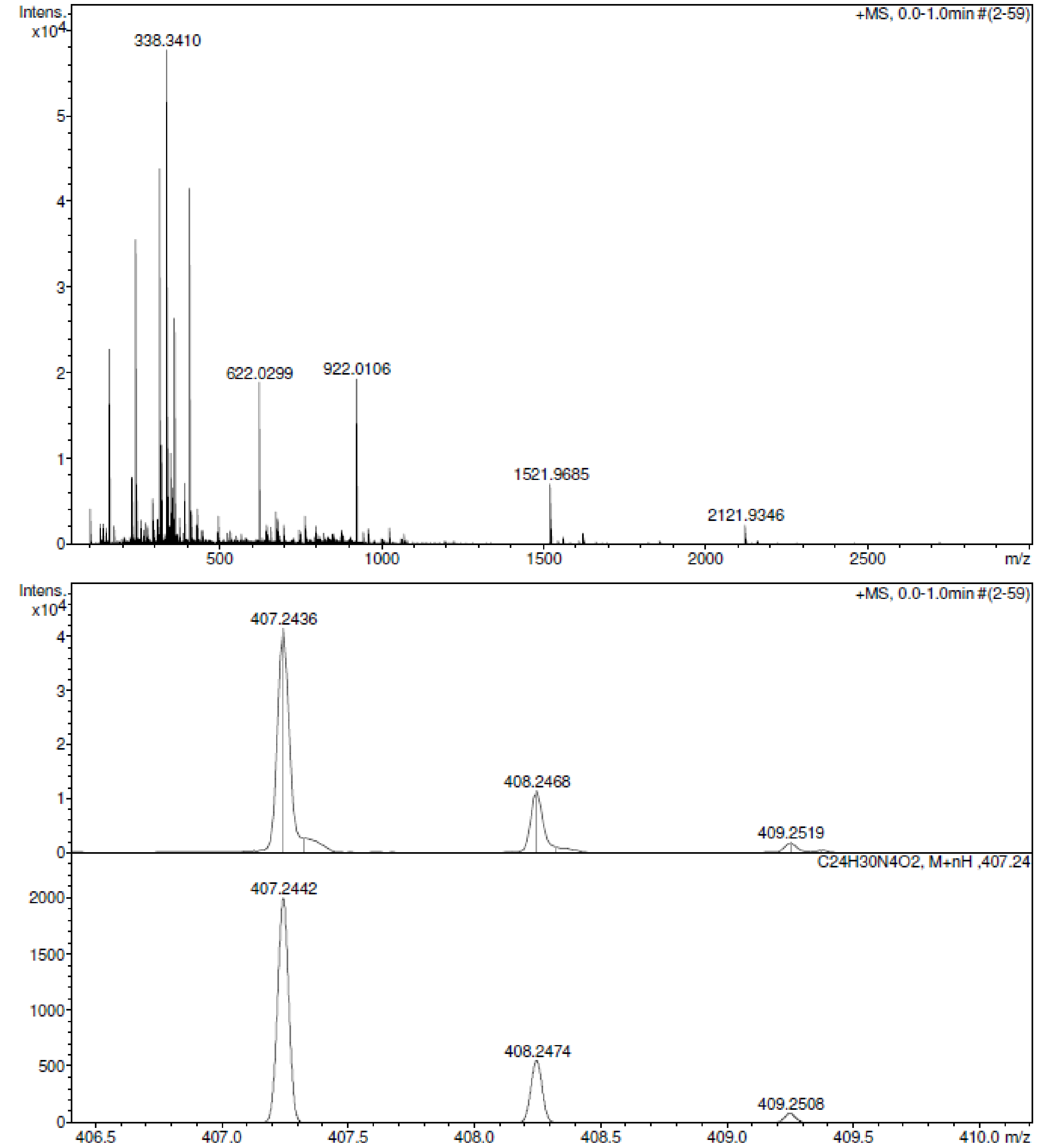

HRMS spectrum of compound **1j**

Acquisition Parameter

Source Type	ESI	Ion Polarity	Positive	Set Nebulizer	0.4 Bar
Focus	Not active			Set Dry Heater	180 °C
Scan Begin	50 m/z	Set Capillary	4500 V	Set Dry Gas	4.0 l/min
Scan End	3000 m/z	Set End Plate Offset	-500 V	Set Divert Valve	Waste

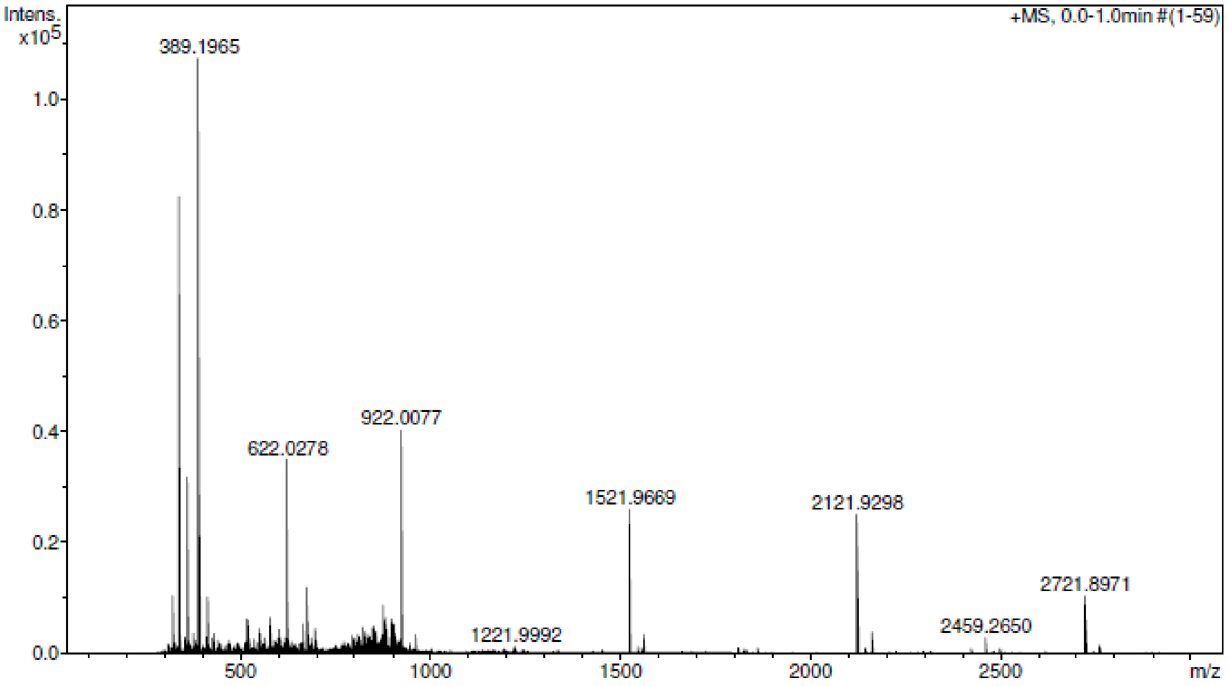

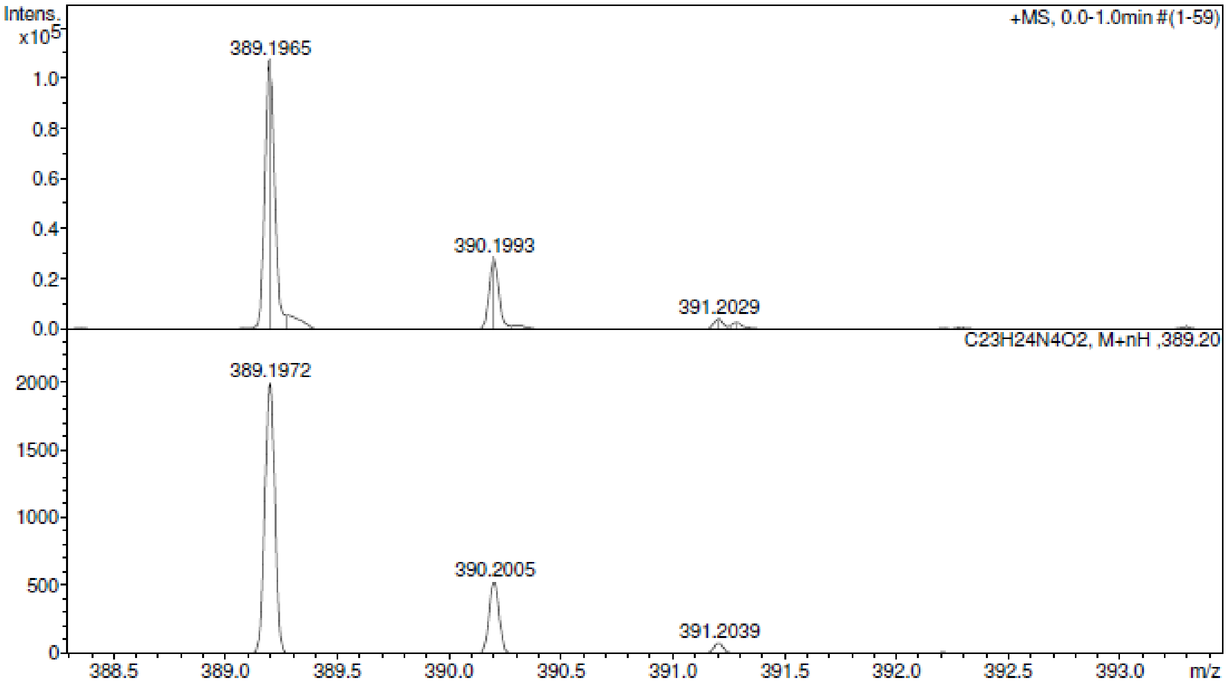

HRMS spectrum of compound **1k**

Acquisition Parameter

Source Type	ESI	Ion Polarity	Positive	Set Nebulizer	0.4 Bar
Focus	Not active			Set Dry Heater	180 °C
Scan Begin	50 m/z	Set Capillary	4500 V	Set Dry Gas	4.0 l/min
Scan End	3000 m/z	Set End Plate Offset	-500 V	Set Divert Valve	Waste

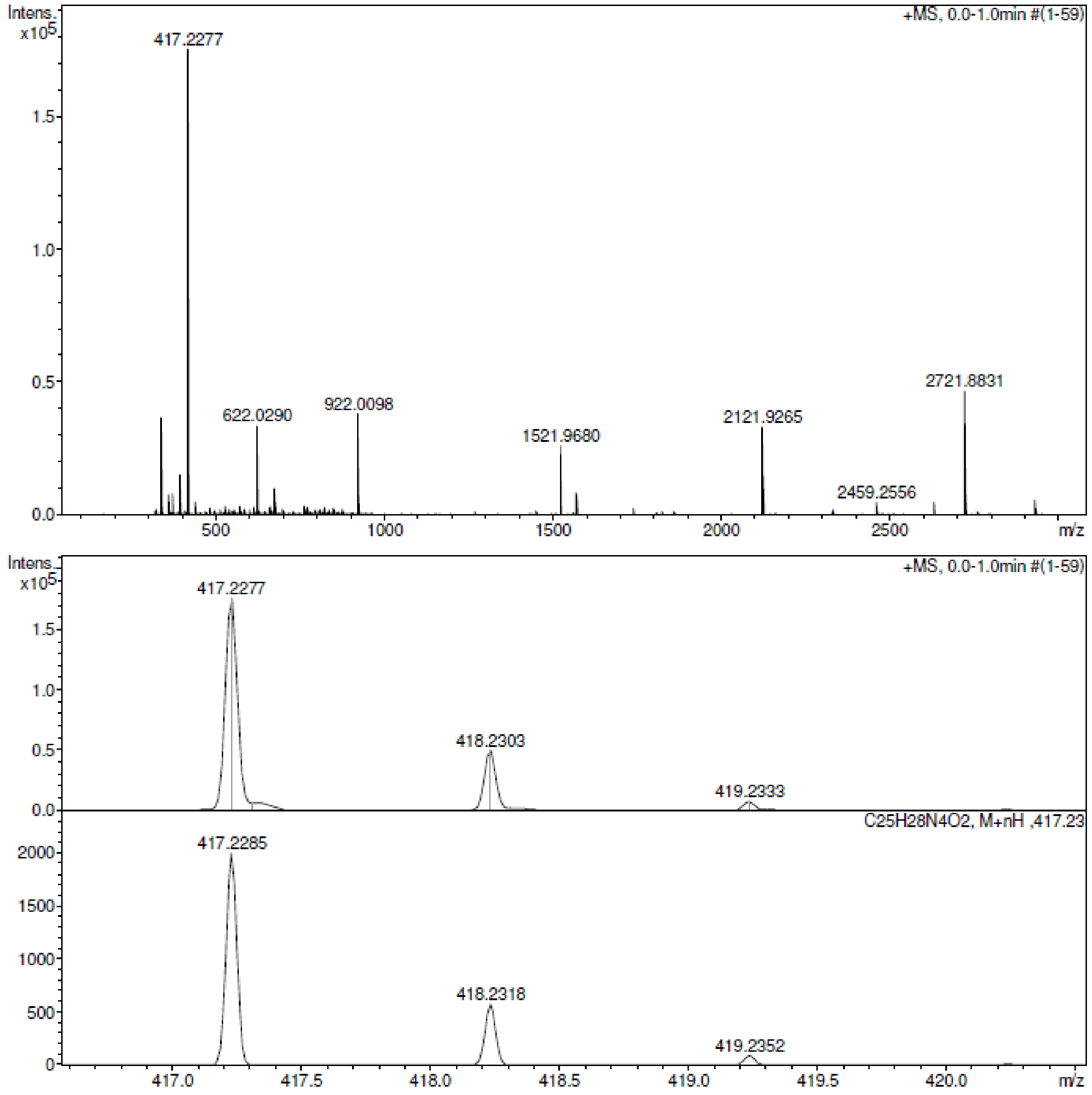

Bruker Compass DataAnalysis 4.0 printed: 27.06.2022 14:28:45 Page 1 of 1

HRMS spectrum of compound **1l**

Acquisition Parameter

Source Type	ESI	Ion Polarity	Positive	Set Nebulizer	0.4 Bar
Focus	Not active			Set Dry Heater	180 °C
Scan Begin	50 m/z	Set Capillary	4500 V	Set Dry Gas	4.0 l/min
Scan End	3000 m/z	Set End Plate Offset	-500 V	Set Divert Valve	Waste

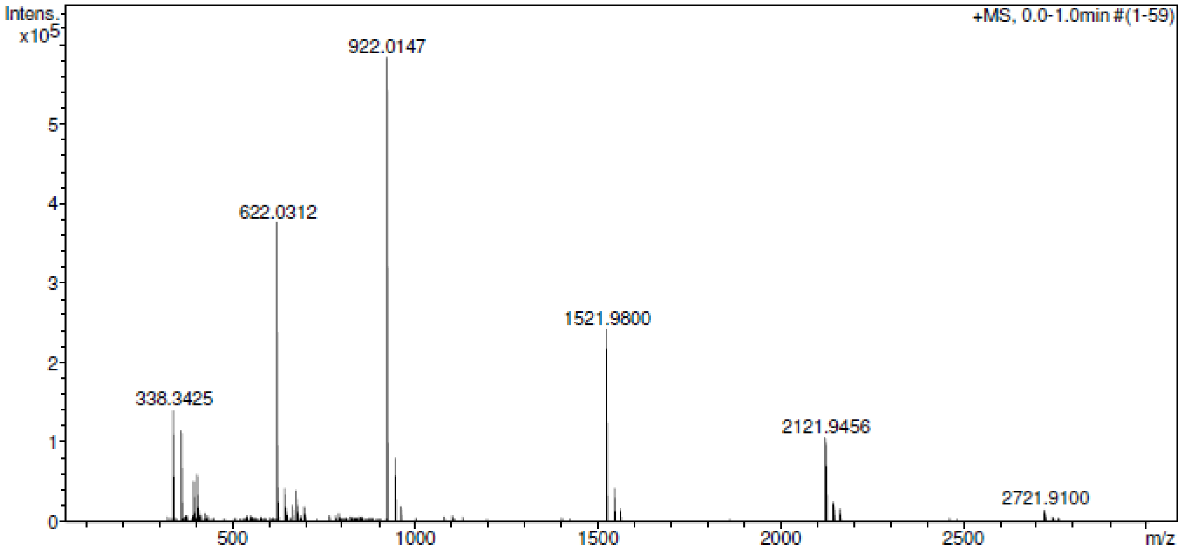

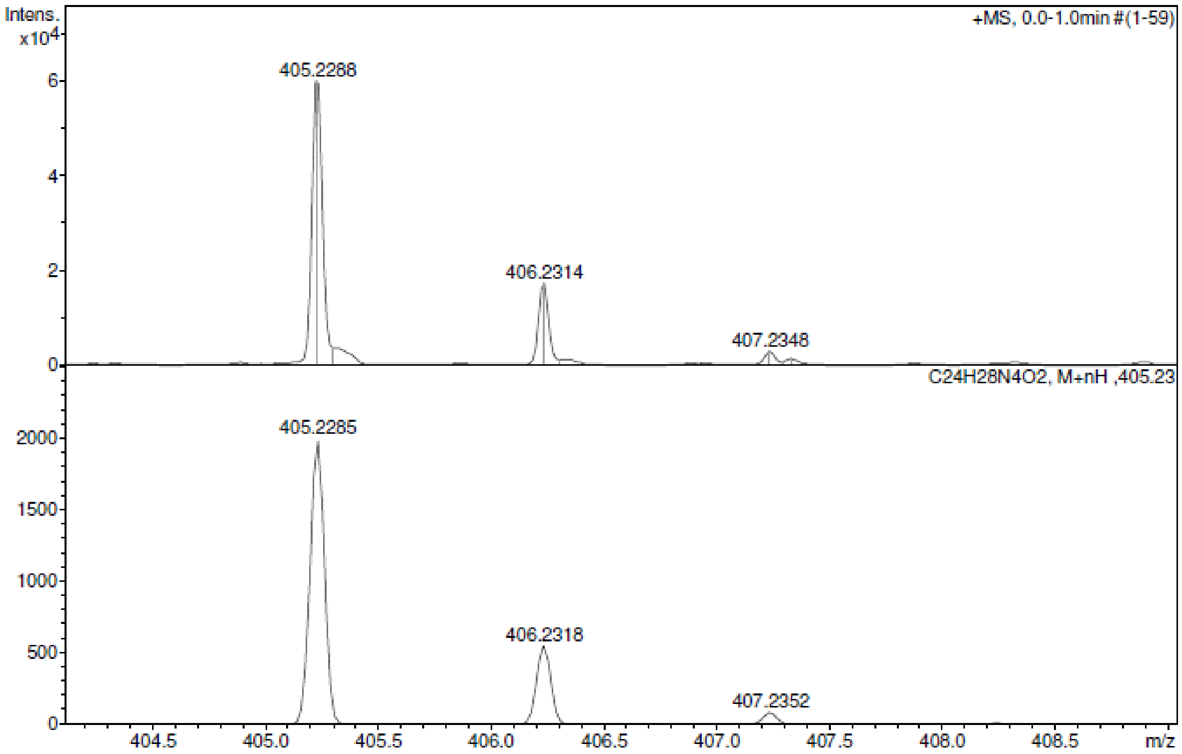

HRMS spectrum of compound **1m**

Acquisition Parameter

Source Type	ESI	Ion Polarity	Positive	Set Nebulizer	0.4 Bar
Focus	Not active			Set Dry Heater	180 °C
Scan Begin	50 m/z	Set Capillary	4500 V	Set Dry Gas	4.0 l/min
Scan End	3000 m/z	Set End Plate Offset	-500 V	Set Divert Valve	Waste

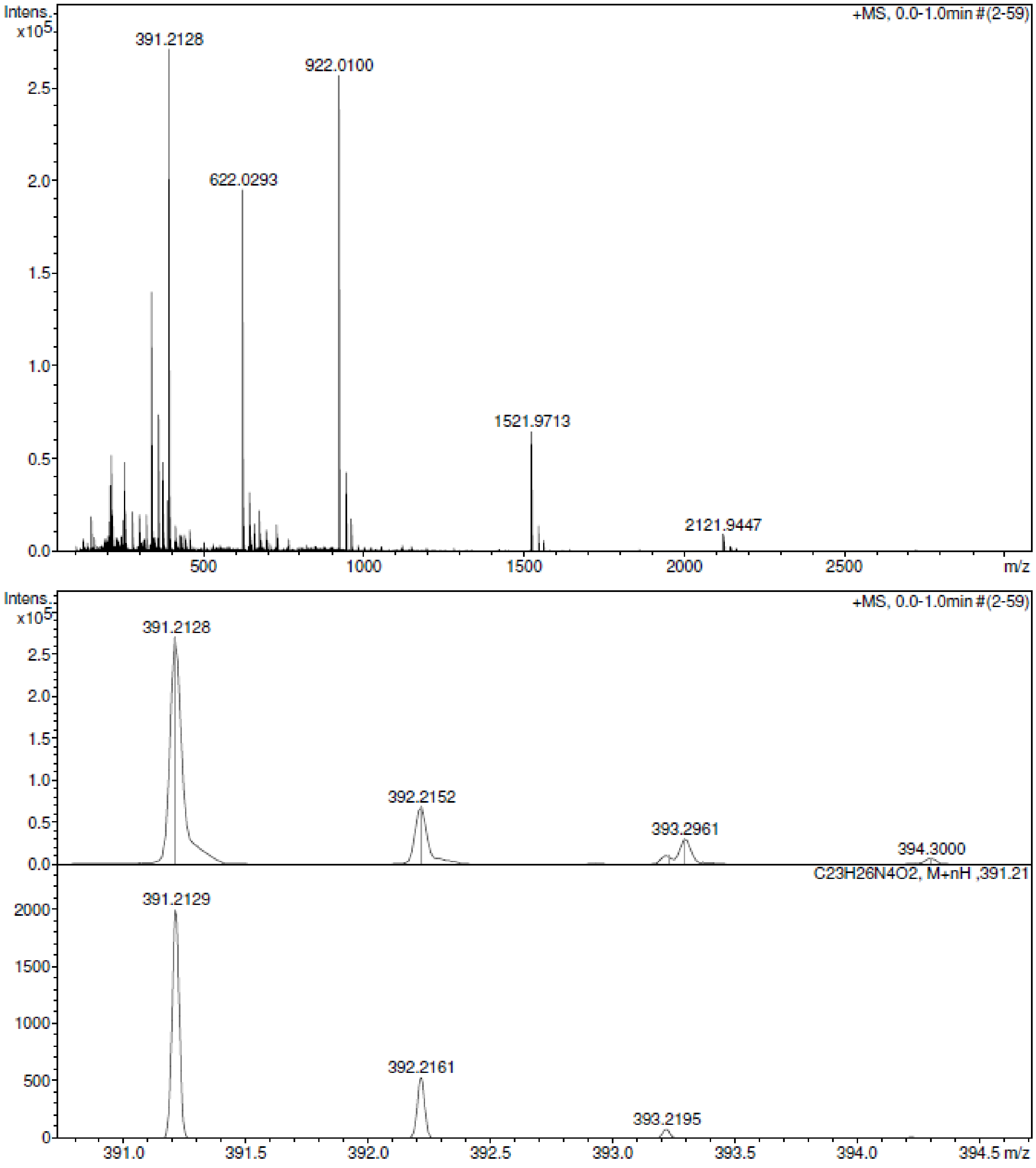

HRMS spectrum of compound **1n**

Acquisition Parameter

Source Type	ESI	Ion Polarity	Positive	Set Nebulizer	0.4 Bar
Focus	Not active			Set Dry Heater	180 °C
Scan Begin	50 m/z	Set Capillary	4500 V	Set Dry Gas	4.0 l/min
Scan End	3000 m/z	Set End Plate Offset	-500 V	Set Divert Valve	Waste

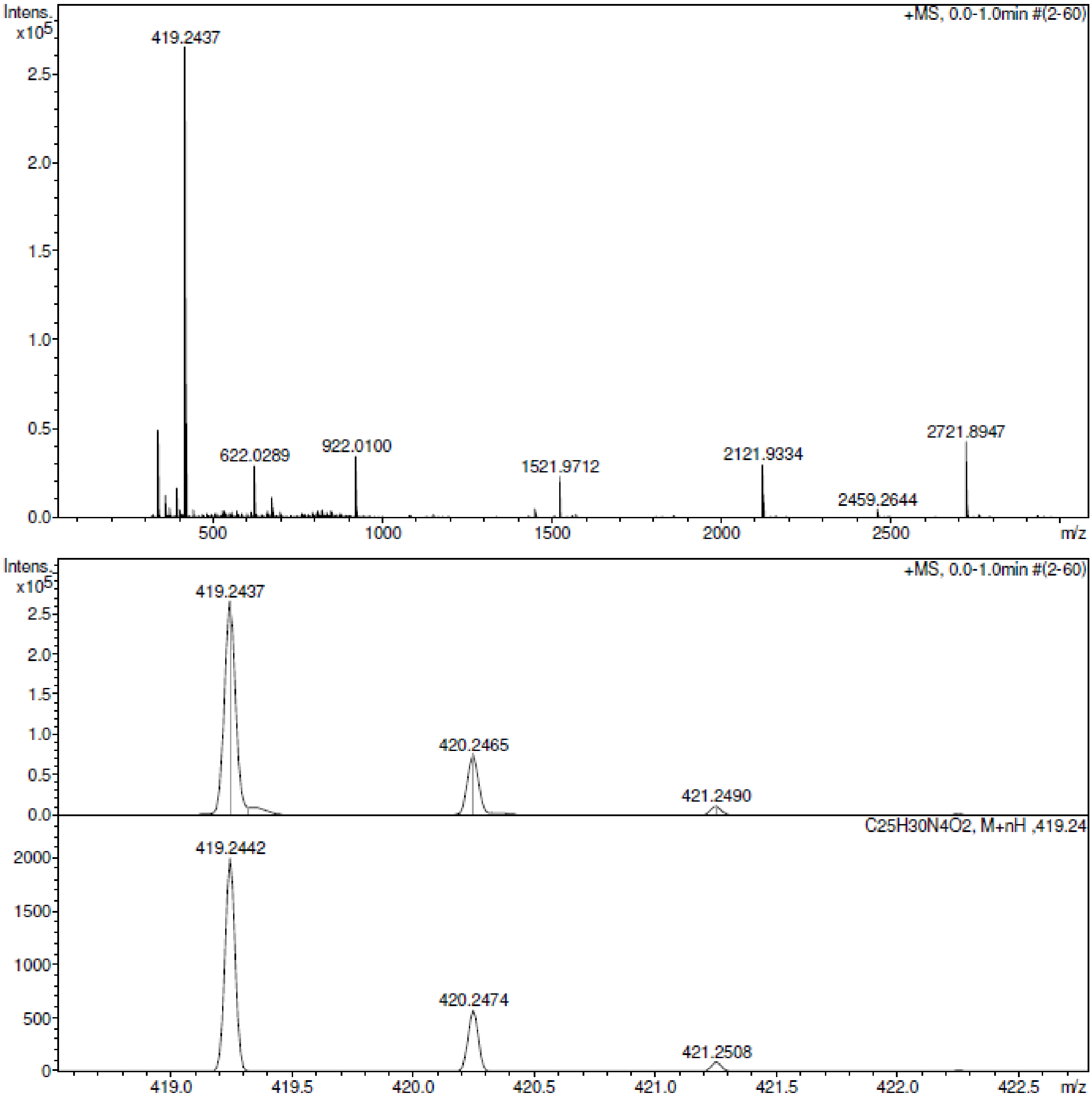

HRMS spectrum of compound **1o**

Acquisition Parameter

Source Type	ESI	Ion Polarity	Positive	Set Nebulizer	0.4 Bar
Focus	Not active			Set Dry Heater	180 °C
Scan Begin	50 m/z	Set Capillary	4500 V	Set Dry Gas	4.0 l/min
Scan End	3000 m/z	Set End Plate Offset	-500 V	Set Divert Valve	Waste

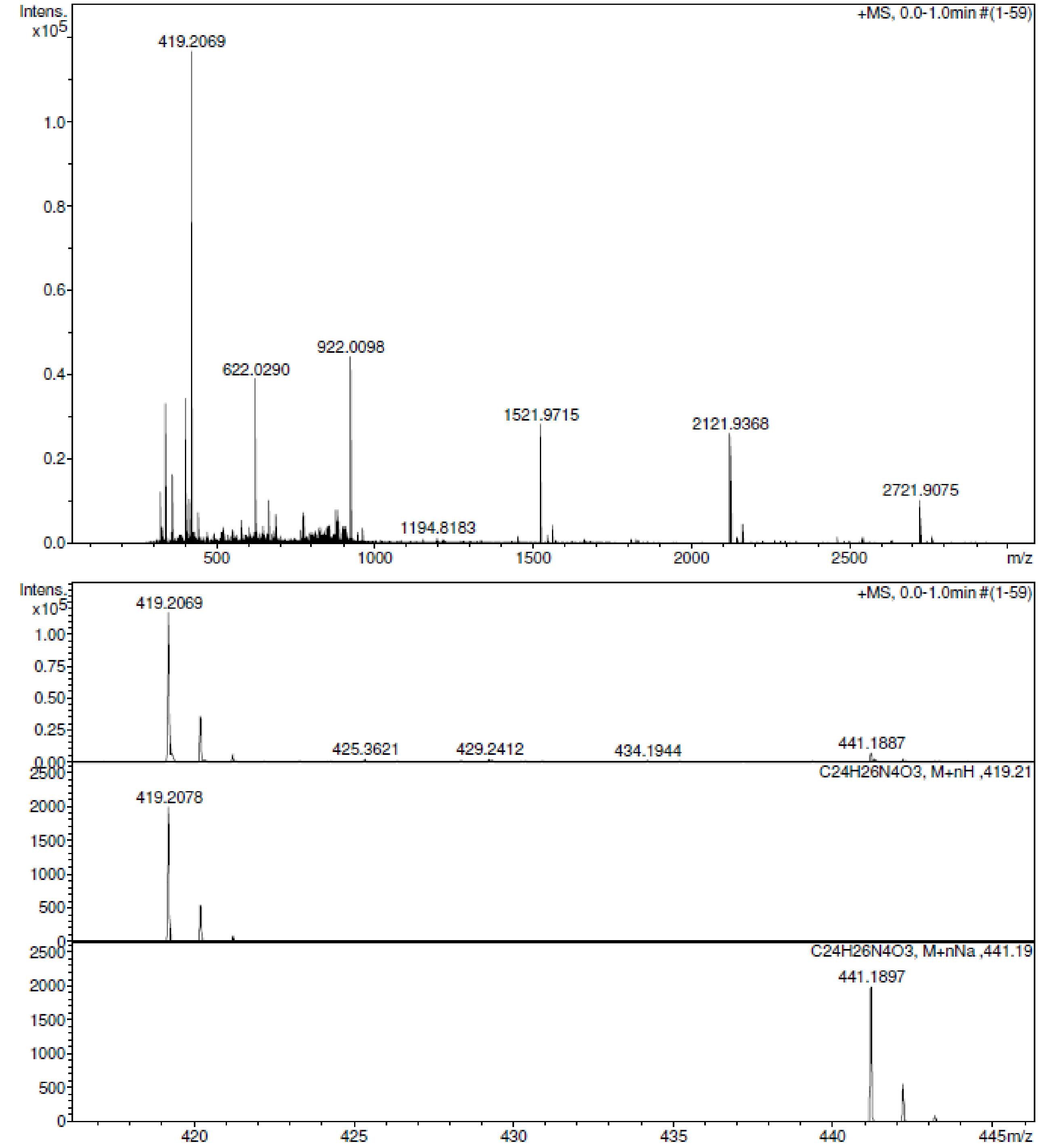

4. Kainate-induced AMPA receptor currents

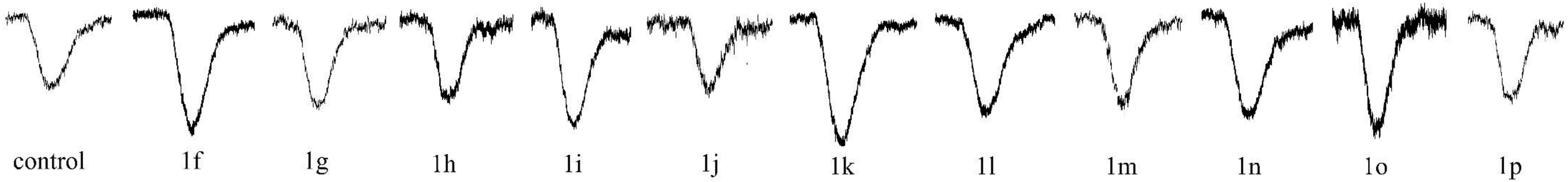

Figure S1. Kainate-induced AMPA receptor currents recorded in electrophysiological evaluation of compounds **1f–1p** at 10^{-9} M concentration (left – control).

5. Parameters of molecular dynamics simulation of the modulator-receptor complexes

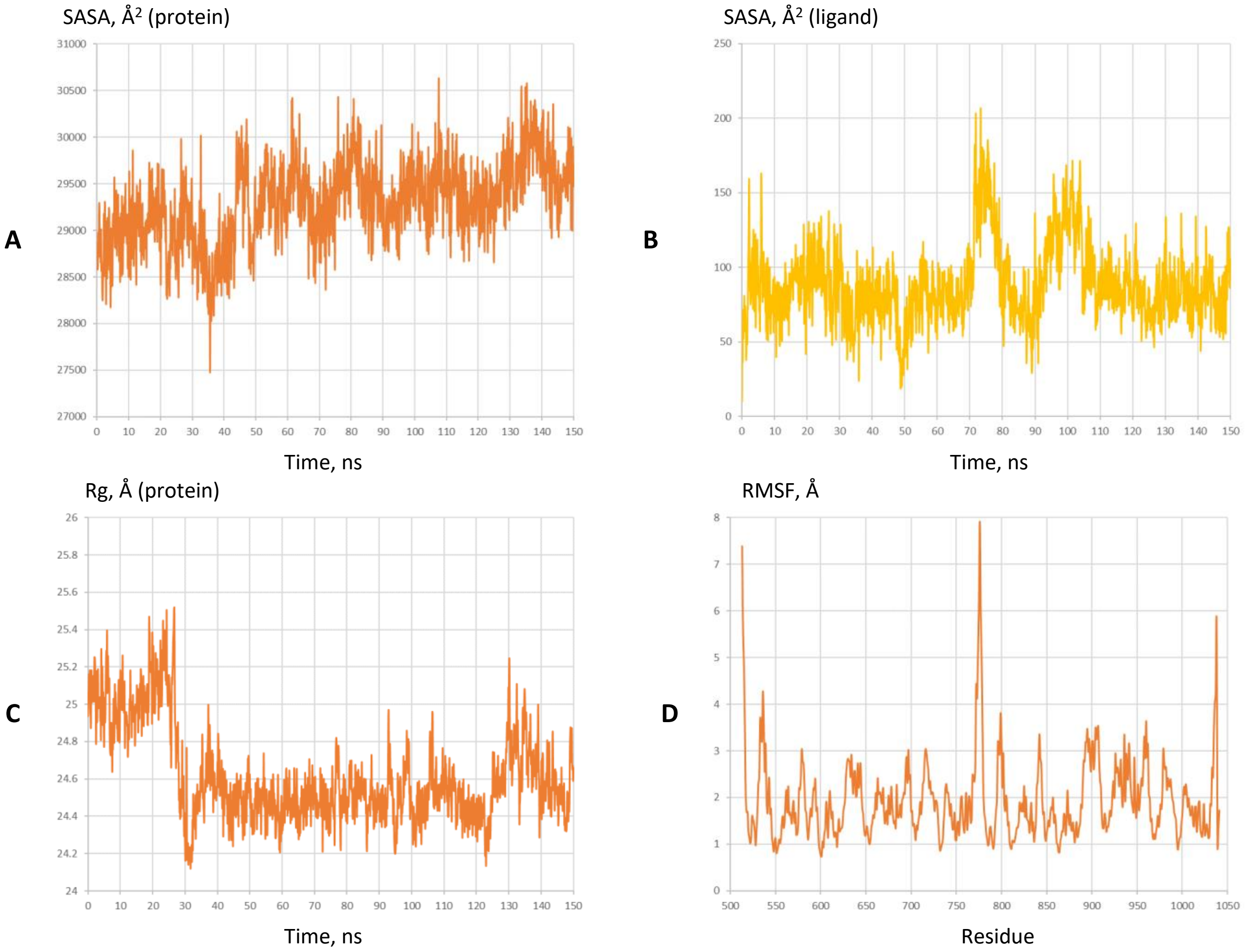

Figure S2. Parameters of molecular dynamics simulation of the modulator **1f** complex with the dimeric ligand binding domain of the GluA2 AMPA receptor. (**A**) Protein solvent-accessible surface area (SASA). (**B**) Ligand solvent-accessible surface area (SASA). (**C**) Protein mass-weighted radius of gyration. (**D**) Root mean square fluctuations by residue. Peaks correspond to the ends of the protein chains; the last three points represent the glutamate agonist molecules (1039, 1040) and the ligand (1041).

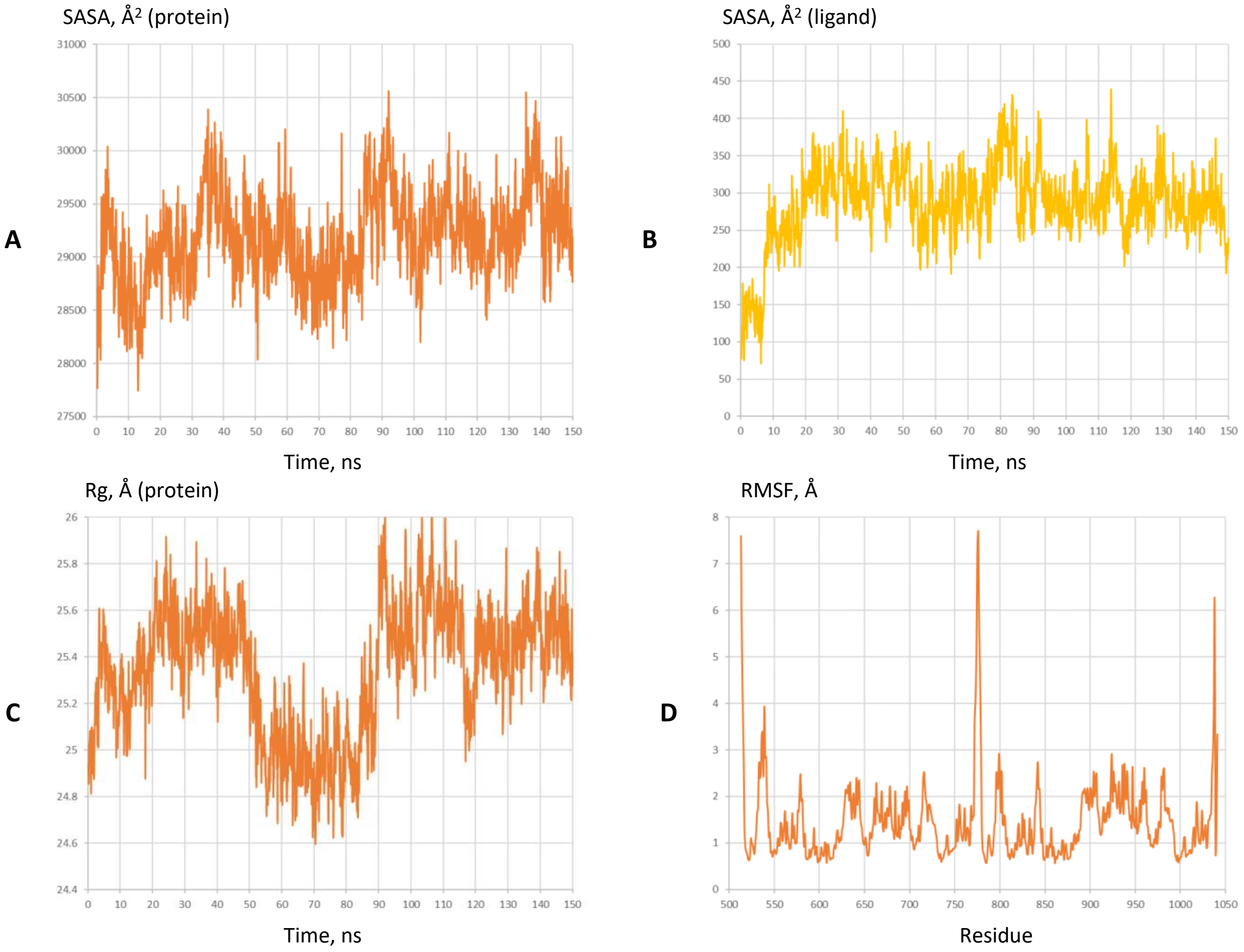

Figure S3. Parameters of molecular dynamics simulation of the modulator **1i** complex with the dimeric ligand binding domain of the GluA2 AMPA receptor. (**A**) Protein solvent-accessible surface area (SASA). (**B**) Ligand solvent-accessible surface area (SASA). (**C**) Protein mass-weighted radius of gyration. (**D**) Root mean square fluctuations by residue. Peaks correspond to the ends of the protein chains; the last three points represent the glutamate agonist molecules (1039, 1040) and the ligand (1041).

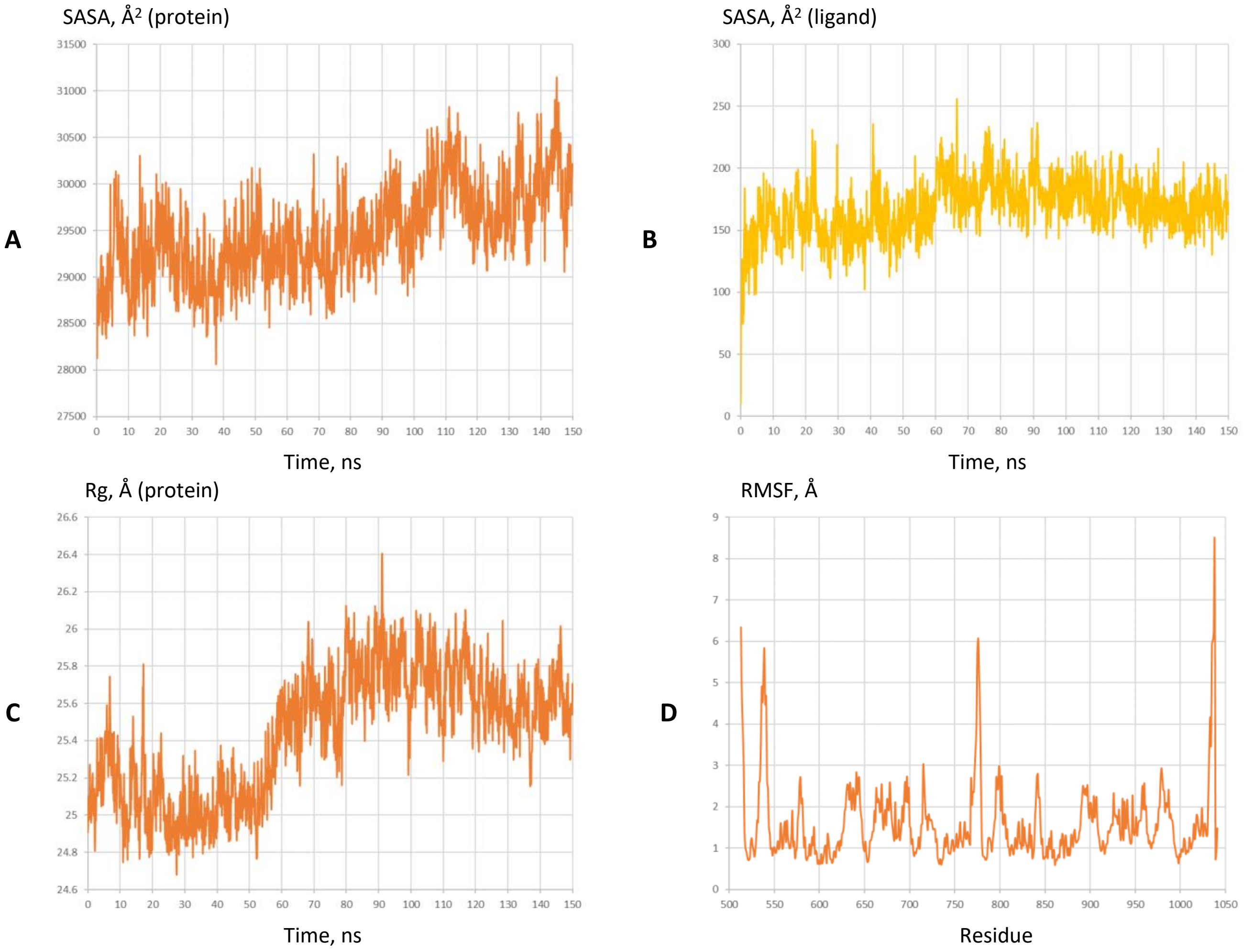

Figure S4. Parameters of molecular dynamics simulation of the modulator **1j** complex with the dimeric ligand binding domain of the GluA2 AMPA receptor. (**A**) Protein solvent-accessible surface area (SASA). (**B**) Ligand solvent-accessible surface area (SASA). (**C**) Protein mass-weighted radius of gyration. (**D**) Root mean square fluctuations by residue. Peaks correspond to the ends of the protein chains; the last three points represent the glutamate agonist molecules (1039, 1040) and the ligand (1041).
